# CH02 peptide promotes *ex vivo* expansion of umbilical cord blood-derived CD34 ^+^ hematopoietic stem/progenitor cells: CH02 peptide promotes CD34 ^+^ UCB-HSPC *ex vivo* expansion

**DOI:** 10.3724/abbs.2023047

**Published:** 2023-06-28

**Authors:** Yiqi Yang, Bihui Zhang, Junye Xie, Jingsheng Li, Jia Liu, Rongzhan Liu, Linhao Zhang, Jinting Zhang, Zijian Su, Fu Li, Leisheng Zhang, An Hong, Xiaojia Chen

**Affiliations:** 1 Institute of Biomedicine & Department of Cell Biology College of Life Science and Technology Guangdong Province Key Laboratory of Bioengineering Medicine Guangdong Provincial Biotechnology Drug & Engineering Technology Research Center; National Engineering Research Center of Genetic Medicine Ji’nan University Guangzhou 510632 China; 2 The First Affiliated Hospital Ji’nan University Guangzhou 510630 China; 3 Key Laboratory of Molecular Diagnostics and Precision Medicine for Surgical Oncology in Gansu Province & NHC Key Laboratory of Diagnosis and Therapy of Gastrointestinal Tumor Gansu Provincial Hospital Lanzhou 730000 China; 4 Key Laboratory of Radiation Technology and Biophysics Hefei Institute of Physical Science Chinese Academy of Sciences Hefei 230031 China

**Keywords:** CH02 peptide, umbilical cord blood, CD34 ^+^ hematopoietic stem/progenitor cell, Fms-like tyrosine kinase receptor 3, wound healing

## Abstract

Umbilical cord blood (UCB) is an advantageous source for hematopoietic stem/progenitor cell (HSPC) transplantation, yet the current strategies for large-scale and cost-effective UCB-HSPC preparation are still unavailable. To overcome these obstacles, we systematically evaluate the feasibility of our newly identified CH02 peptide for *ex vivo* expansion of CD34 ^+^ UCB-HSPCs. We herein report that the CH02 peptide is specifically enriched in HSPC proliferation via activating the FLT3 signaling. Notably, the CH02-based cocktails are adequate for boosting 12-fold *ex vivo* expansion of UCB-HSPCs. Meanwhile, CH02-preconditioned UCB-HSPCs manifest preferable efficacy upon wound healing in diabetic mice via bidirectional orchestration of proinflammatory and anti-inflammatory factors. Together, our data indicate the advantages of the CH02-based strategy for *ex vivo* expansion of CD34 ^+^ UCB-HSPCs, which will provide new strategies for further development of large-scale HSPC preparation for clinical purposes.

## Introduction

Hematopoietic stem/progenitor cells (HSPCs) have been acknowledged as the most undifferentiated blood cells for all kinds of functional blood cell generation and concomitant hematopoietic reconstitution during hematologic malignancies. To date, a variety of sources have been reported for HSPC preparation, such as bone marrow (BM), mobilized peripheral blood (mPB), umbilical cord blood (UCB), placental blood and even human pluripotent stem cells [ 1, 2]. Among them, “discarded” UCB is recognized as one of the richest sources for HSPC transplantation due to its properties, including easy availability, low immunogenicity and low incidence of graft versus host disease (GVHD) [ 3, 4]. However, due to the deficiency in the amounts of HSPCs from single unit UCB, the large-scale clinical application of UCB-HSPCs is largely obscure [5].

For decades, a variety of strategies have been explored for the efficient generation of UCB-HSPCs via optimizing the *ex vivo* expansion of CD34 ^+^ cells for HSPC transplantation [ 6‒ 8]. For instance, several procedures demonstrated the feasibility of *ex vivo* expansion of HSPCs by co-culturing with feeder cells ( *e*. *g*., mesenchymal stem/stromal cells) [ 7, 9, 10], bioreactors, cytokine cocktails containing stem cell factor (SCF) [ 11, 12], thrombopoietin (TPO) [13], Fms-like tyrosine kinase receptor 3 ligand (FLT3L) [ 14‒ 16], interleukin-3, interleukin-6 and interleukin-11 [ 17, 18], and even small molecules and chemicals such as tetraethylenpentamin (TEPA) [19]. Despite considerable progress in regulating HSPC maintenance and expansion, most of the current strategies for CD34 ^+^ cell expansion *in vitro* are either inefficient or costly and thus cannot fulfil the large-scale clinical application of HSPC-based cytotherapy.

In the present study, based on a newly identified CH02 peptide (sequence: GPANVET) with 7 amino acid residues and efficacy upon dorsal root crush injury [20], we further explored the cellular biofunctions and molecular mechanism of the CH02 peptide during *ex vivo* expansion of UCB-HSPCs. With the aid of target capture, we found that CH02 showed the highest binding affinity and abundance to FLT3, which is involved in regulating the growth of primitive hematopoietic progenitor cells [21]. Notably, we found that the combination of the CH02 peptide with the indicated cytokine cocktails in serum-free medium could effectively promote the proliferation of CD34 ^+^ cells and maintain their stem cell properties. Moreover, UCB-HSPCs preconditioned with the CH02 peptide significantly promoted wound healing in diabetic mice by simultaneously modulating the anti-inflammatory and proinflammatory factors. Collectively, the CH02 peptide served as a novel and cost-effective cytokine that could efficiently boost the *ex vivo* expansion of UCB-HSPCs for clinical purposes in the future.

## Materials and Methods

### Peptide synthesis

The CH02 peptide (sequence: GPANVET) was synthesized by Top-Peptide Biotechnology Co. Ltd. (Shanghai, China) with a purity over 98%, and the molecular weight was determined by mass spectrometry ( Supplementary Figure S1).

### Cell culture and cell viability assay

Human umbilical vein endothelial cells (HUVECs), human splenic fibroblasts (HSFs) and NIH/3T3 cells were obtained from the Institute of Biochemistry and Cell Biology of the Chinese Academy of Sciences (Shanghai, China). All cell lines were cultured in DMEM basal medium supplemented with 10% fetal bovine serum (FBS), 100 IU/mL penicillin and 100 μg/mL streptomycin at 37°C with 5% CO _2_.

For cell viability assay, cells were seeded in a 96-well plate at 2×10 ^3^ cells/well and cultured in the aforementioned medium for 24 h and then starved for 12 h with fresh DMEM basal medium supplemented with 1% FBS. Therewith, cells were treated with CH02 peptide at the indicated concentrations for 48 h. Finally, cell viability was measured using the Cell Counting Kit-8 (CCK-8; Dojindo Laboratories, Tokyo, Japan), and the absorbance was quantified at 450 nm with the Infinite® F50 microplate reader (Tecan, Männedorf, Switzerland).

### Phosphoproteomic and Gene Ontology (GO) analyses

Phosphoproteomic analysis data were available as previously reported [20]. Differentially expressed phosphorylated peptides (DEPPs) were screened ( *P*<0.1), as shown in Supplementary Table S1. GO biological process (GOBP) analysis of the DEPPs was performed with the Gene Ontology Resource website ( http://geneontology.org/). *P*<0.05 was regarded as statistically significant.

### Molecular docking and dynamic simulation analysis

Molecular docking was performed as previously described [20]. Briefly, the protein sequence of FLT3 (NP_004110) was generated from the National Center for Biotechnology Information (NCBI), while the template file of the extracellular segment of FLT3 was obtained from the Protein Data Bank (PDB ID: 3QS7). Homology modelling of the FLT3 extracellular segment was performed using the SWISS-MODEL online server [ 22, 23]. Molecular docking between the CH02 peptide and FLT3 was performed using the LeDock software, and the best conformation was selected based on the docking binding energy and binding pose. The results were visualized using PyMOL and Discovery Studio 2017 clients.

All-atom molecular dynamics simulations were performed on the CH02-FLT3 docking complex using the CHARMM36 force field by GROMACS 2020.6 software as previously described [ 20 , 24]. Briefly, topology and coordinate files for FTL3 and CH02 were generated by the GROMACS pdb2gmx algorithm, and the MD simulations of the CH02-FTL3 docking complex were run in a periodic boundary condition (PBC) cubic box with water (model: TIP3P) as a solvent, followed by 150 mM chloride and sodium ions to simulate the physiological environment. The CH02-FLT3 docking complex was simulated for 50 ns, the simulation step was 2 fs, and the coordinates were recorded every 0.2 ps. The average structure and dynamic trajectories of the CH02-FLT3 docking complex were visualized using PyMOL.

### Western blot analysis

Cells at the indicated time points were collected and lysed using RIPA buffer containing protease and phosphatase inhibitor cocktail (Cat. #78442; Thermo Scientific, Shanghai, China) and 1 mM phenylmethanesulfonyl fluoride (PMSF). Approximately 20 μg of protein lysate was subject to SDS-PAGE and transferred onto PVDF membranes (Millipore, Billerica, USA). After blocking, the membranes were incubated with the indicated primary antibodies. The primary antibodies used were as follows: anti-phospho-Flt3/CD135 (Cat. #ab171953; Abcam, Cambridge, UK), anti-Flt3/CD135 (Cat. #ab171953; Abcam), anti-phospho-ERK1/2 (Cat. #ab76299; Abcam), anti-ERK1/2 (Cat. #9102; CST, Danvers, USA), anti-AKT (Cat. #9272; CST), and anti-phospho-AKT (Cat. #9271; CST). Then, the membranes were incubated with HRP-conjugated secondary antibody (CST), and the protein bands were visualized using the Enhanced chemiluminescence (ECL) substrates (Thermo Scientific, Shanghai, China) and analysed with a digital gel image analysis system (Tanon, Shanghai, China).

### MACS-based enrichment of CD34 ^+^ UCB-HSPCs

The studies involving human participants were reviewed and approved by the Medical Ethics Committee of Ji’nan University (Guangzhou, China). UCB was collected from normal full-term deliveries at the First Affiliated Hospital of Jinan University with informed consent and the approval of the Ethics Committee of Ji’nan University. Briefly, mononuclear cells (MNCs) were isolated by Ficoll-Hypaque-based gradient centrifugation, and CD34 ^+^ cells were enriched by using a Miltenyi-MACS CD34 ^+^ selection kit (Cat. #130-046-702; Miltenyi, Bergisch Gladbach, Germany) according to the manufacturer’s instructions. Detailed information on UCB samples and CD34 ^+^ HSPCs after enrichment is available in Supplementary Table S2.

### *Ex vivo* expansion of CD34 ^+^ UCB-HSPCs in serum-free medium

The enriched CD34 ^+^ UCB-HSPCs were seeded at 5×10 ^4^ cells/mL in SFEM medium (#09650; Stem Cell Technologies, Vancouver, Canada) with different cytokine additions for 7 days, and half of the medium was refreshed every 48 h. The cytokine cocktails included SIT (SCF, IL-6, and TPO), SITF (SCF, IL-6, TPO, and FLT3L), and SITH (SCF, IL-6, TPO, and CH02). The concentrations of the cytokines were 100 ng/mL SCF (Cat. #300-07-5; PeproTech, Rocky Hill, USA), 20 ng/mL IL-6 (Cat. #200-06-5; PeproTech;), and 100 ng/mL TPO (Cat. #300-18-50; PeproTech), 100 ng/mL FLT3 ligand (Cat. #300-19-10; PeproTech), and CH02 peptide (5, 10, 15 or 20 ng/mL)

### Flow cytometry (FCM) analysis

FCM analysis of the enriched and *ex vivo* expanded UCB-HSPCs was performed as previously reported [25]. In brief, cells were harvested and washed with 1× PBS twice and then incubated with the indicated antibodies, including CD34-FITC and CD38-APC antibodies (BD Biosciences, Burlington, Canada) and the isotype controls, at 4°C for 30 min in the dark. The labelled cells were washed with 1× PBS and subject to FCM analysis on a FACScan flow cytometer (Beckman Coulter, Pasadena, USA). A total of 50,000 events were recorded and analysed with the FlowJo 6.0 software (Stanford University, Stanford, USA).

### Colony-forming unit (CFU) assay of UCB-HSPCs

The enriched and *ex vivo* expanded CD34 ^+^ UCB-HSPCs under different cytokine cocktail stimulations for 7 days were seeded at 1000 cells/mL in semisolid culture (MethoCult™ H4434 Classic; Stem Cell Technologies) according to the manufacturer’s instructions. After incubation at 37°C with 5% CO _2_ at 100% humidity for 14 days, the number of burst-forming unit-erythroid (BFU-E), colony-forming unit-erythroid (CFU-E), colony-forming unit-granulocyte/macrophage (CFU-GM) and colony-forming unit-granulocyte, erythroid, macrophage, megakaryocyte (CFU-GEMM) were recorded under an inverted microscope (Nikon, Shanghai, China). The numbers of CFU per 1000 CD34 ^+^ UCB-HSPCs before cultivation (day 0) and after harvest (day 7) were calculated.

### Dorsal wound mouse model for transplantation

The animal studies were conducted according to the guidelines of the Declaration of Helsinki and approved by the Ethics Committee of Animal Experiments of Ji’nan University. Six- to seven-week-old male db/db mice (BKS-Leprem2Cd479/Nju, Leprdb mut/mut) and male C57BL/6J mice (Leprdb wt/wt) were purchased from Nanjing Biomedical Research Institute (Nanjing, China) and left for 2 weeks with cages changed daily. The mouse was treated with 3% pentobarbital sodium anaesthesia (5 mg/kg), the dorsal surface was shaved, and the fur was completely removed by a depilatory cream. The donut-shaped splint was fixed to the surrounding skin by bonding adhesive and 6-0 silk sutures, and a full-thickness wound was created by a sterile disposable 6-mm biopsy punch. A total of 2×10 ^5^ cells were injected into the surface of the wounds (overlay) or around the wound edge and covered with 20 μL of fibrin in the experimental groups, while the control wounds were injected with 1× PBS.

### Measurement of the wound area

The wound area was monitored with a scale every 48 h by taking photos using a digital camera for 21 days. The silicon splints were used as a size reference in the analysis. Pictures were analyzed by drawing around the wound margins and measuring pixel area using ImageJ (NIH, Bethesda, USA).

### RNA sequencing and bioinformatics analysis

RNA samples were prepared from CD34 ^+^ UCB-HSPCs cultured in the indicated medium. Total mRNA was extracted using an RNAisoPlus kit (TaKaRa, Kusatsu, Japan) and reverse transcribed into cDNAs with random primers. Then, the cDNAs were purified with a Qiaquick PCR extraction kit (Qiagen, Hilden, Germany), end-repaired and ligated to sequencing adapters. The cDNA libraries were sequenced on the Illumina sequencing platform (Illumina, San Diego, USA) by Novogene Biotechnology [26]. Paired-end clean reads were used for mapping to the genome with HISAT 2.2.4 [27]. The mapped reads of each individual sample were assembled using StringTie v1.3.1 in a reference-based approach [28]. For each transcription region, the fragment per kilobase of transcript per million mapped reads (FPKM) value was calculated to quantify its expression abundance and variations. Differential gene expression analysis was conducted by DESeq2 software [29]. Genes with a false discovery rate (FDR)≤0.05 and absolute fold change≥2 were filtered as DEGs. Then, DEGs were mapped to GO terms in the GO database ( http://www.geneontology.org/), and gene numbers were calculated for each time. Significantly enriched GO terms in DEGs compared with the background were defined by a hypergeometric test. Target gene set pathway analysis was conducted with Ingenuity Pathway Analysis software (Qiagen) [30].

### Statistical analysis

The statistical analyses were performed using the GraphPad Prism 9.0 (GraphPad Software Inc., San Diego, USA) software. The comparison between two groups was performed by Student’s *t* test, and comparisons among various treatment groups were performed with one-way analysis of variance (ANOVA) followed by Dunnett’s multiple comparisons test. The data are shown as the mean ± standard deviation. A value of *P*< 0.05 was considered statistically significant.

## Results

### Phosphoproteomic analysis indicated the potential biofunction of the CH02 peptide in promoting HSPC proliferation

**Figure 1:**
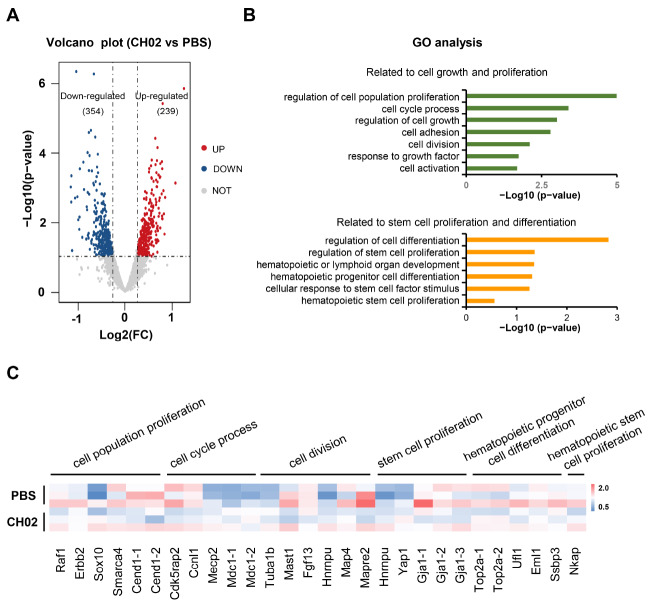
Figure 1 Analysis of the phosphorylated proteomics of DRG tissues treated with CH02 peptide (A) Volcano plot showing the phosphorylated peptides identified by LC–MS/MS. Differentially expressed phosphorylated peptides were screened using the P<0.1 criterion. (B) GO biological process analyses of proteins corresponding to differentially expressed phosphorylated peptides were performed using Gene Ontology Resource. P<0.05 was regarded as statistically significant. (C) Heatmap showing the relative expression level of the phosphorylated peptide in proteins corresponding to the indicated pathway. Each lane represents one independent biological sample.

We previously established a dorsal root injury (DRI) model in rats and found that the CH02 peptide was adequate to promote axon regeneration and sensory function recovery. In this study, we took advantage of the phosphorylated proteomics data of dorsal root ganglion (DRG) tissue after crush injury and found that 593 phosphorylated peptides of 383 proteins were differentially expressed (239 upregulated, 354 downregulated) between the control and CH02-treated groups ( Figure 1A and Supplementary Table S1). With the aid of gene ontology (GO) biological process (GOBP) analysis, we observed that proteins corresponding to the differentially expressed phosphorylated peptides were related to the regulation of cell population proliferation, cell cycle process and cell division, which collectively suggested the promoting effect of CH02 on cell proliferation in DRG tissue ( Figure 1B). Consistently, the CH02 peptide was also involved in promoting the proliferation of multiple cell lines, such as human umbilical vein endothelial cells (HUVECs), human splenic fibroblasts (HSFs) and NIH/3T3 cells ( Supplementary Figure S2A). Notably, the phosphorylated peptides in proteins were differentially expressed and showed an association with stem cell proliferation ( *e*. *g*., Hnrnpu and Gja1), hematopoietic progenitor cell differentiation ( *e*. *g*., Top2a and Eml1) and hematopoietic stem cell proliferation ( *e*. *g*., Nkap) ( Figure 1B,C), which further suggested the potential role of the CH02 peptide during HSPC proliferation. Taken together, these data suggested the modulating effect of the CH02 peptide on cell proliferation and, in particular, on HSPCs by affecting the phosphorylation levels of various proteins.

### The CH02 peptide bound to FMS-like tyrosine kinase 3 (FLT3) and activated the FLT3 signaling pathway

**Figure 2:**
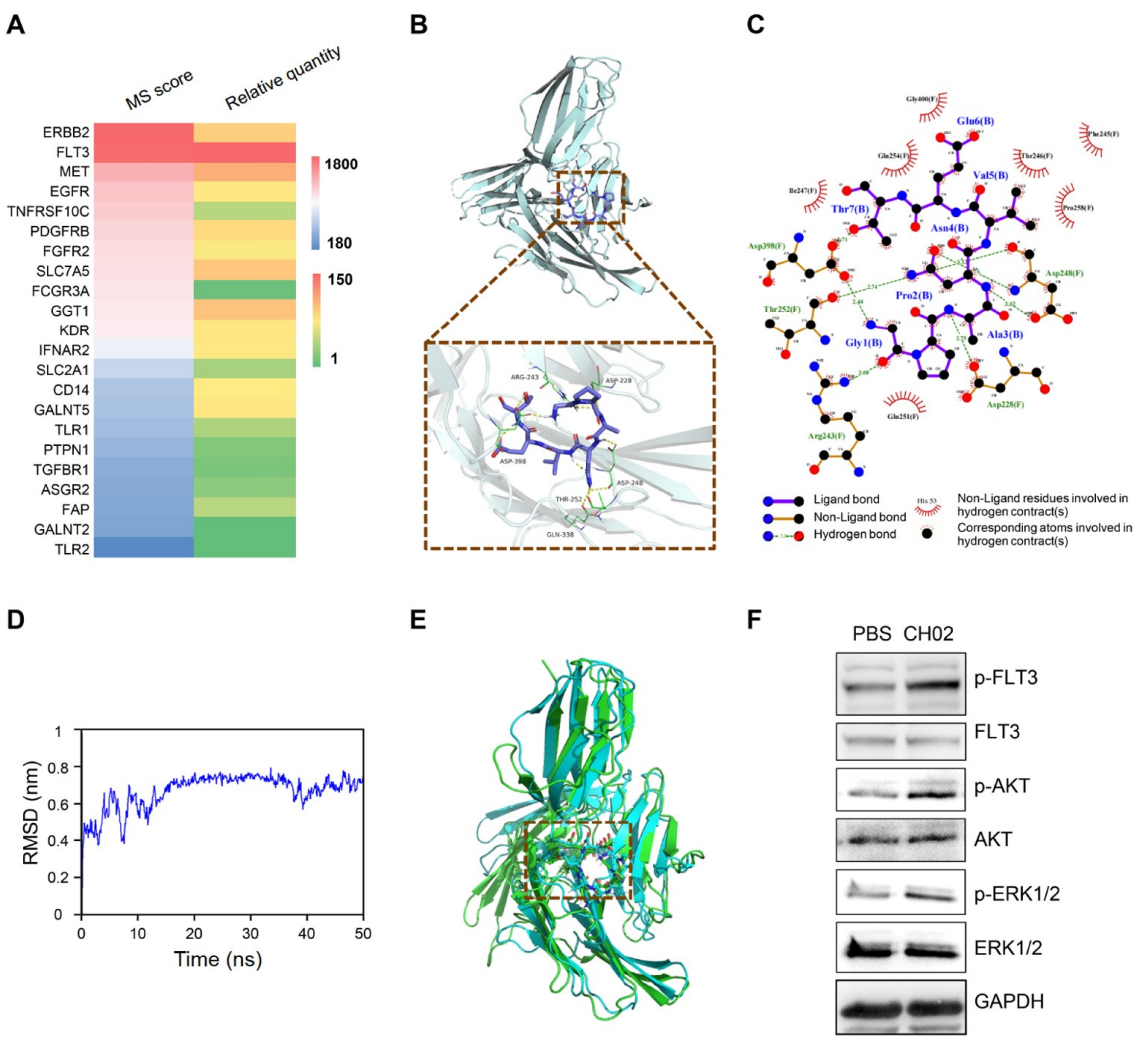
Figure 2 CH02 peptide bound to FLT3 (A) MS score and relative quantity histogram of target proteins captured by SPR and LC-MS/MS. (B,C) Molecular docking analysis of CH02 interactions with FLT3. The binding pattern between CH02 and the FLT3 extracellular segment (B) and the noncovalent interaction between CH02 and FLT3 (C) are shown. (D) Molecular dynamics simulation (MDS) of the CH02-FLT3 docking complex. (E) Overlaid plots of the MD initial state (blue) and MD final state (green). (F) Western blot analysis of phosphorylated FLT3 and its downstream PI3K/AKT expression in HUVECs treated with the CH02 peptide.

To investigate the potential target of the CH02 peptide in promoting cell proliferation, we focused on its target receptors in the cell membrane. Our previous studies showed that the CH02 peptide could bind to multiple RTK family receptors [20]. Interestingly, we found that CH02 could also bind to ERBB2, FLT3, MET and EGFR in HUVECs in addition to FGFR2 ( Figure 2A). Notably, FLT3, a receptor tyrosine kinase expressed almost exclusively in the hematopoietic compartment and involved in the cellular vitality of hematopoietic progenitor cells ( *e*. *g*., survival, proliferation, differentiation) [21], showed the highest binding affinity and abundance with CH02 ( Figure 2A). The interaction between the CH02 peptide and FLT3 suggested a potential regulatory effect on HSPC development.

We next tried to verify the binding mode between the CH02 peptide and FLT3 via molecular docking ( Figure 2B,C). According to the docking results, we found that the best CH02 binding site was located in the 3rd extracellular Ig-like domain (D3) of FLT3 with multiple β-sheet structures ( Figure 2B), which was consistent with the binding pattern of FLT3 and its natural ligand FLT3L [31] and suggested the similarities of CH02 and FLT3L in biofunction. Residues in the CH02 and FLT3 binding pocket included ASP-228, ARG-243, ASP-248, THR-252, GLN-338 and ASP-398, and they all formed conventional hydrogen bonds and attractive charges with CH02 ( Figure 2C), which further suggested that CH02 and FLT3 might form a functional complex under simulated conditions. To further investigate the conformational stability and time-dependent efficiency of CH02 in the binding pocket of FLT3, we performed all-atom molecular dynamics simulation (MDS) of the CH02-FLT3 docking complex in physiological saline solution at a temperature of 300 K for50 ns ( Figure 2D,E). On the basis of root mean square deviation (RMSD) values for heavy atoms of CH02 and FTL3, we confirmed that the overall change in the RMSD of the CH02-FLT3 complex did not exceed 1 nm during the simulation process ( Figure 2D). Meanwhile, their MD initial state and final state were strongly overlaid, which indicated that the CH02 peptide stably bound to the D3 region of the FLT3 extracellular segment under simulated conditions ( Figure 2E).

Finally, western blot analysis showed that the phosphorylation levels of FLT3 and its downstream PI3K/AKT were consistently increased in HUVECs ( Figure 2F) and NIH/3T3 ( Supplementary Figure S2B) cell lines treated with CH02, which suggested that the CH02 peptide activated the FLT3 signaling pathway. Collectively, our data indicated the promoting effect of the CH02 peptide on HSPC proliferation by activating the FLT3 signaling pathway.

### The CH02 peptide promoted *ex vivo* expansion of CD34 ^+^ UCB-HSPCs

**Figure 3:**
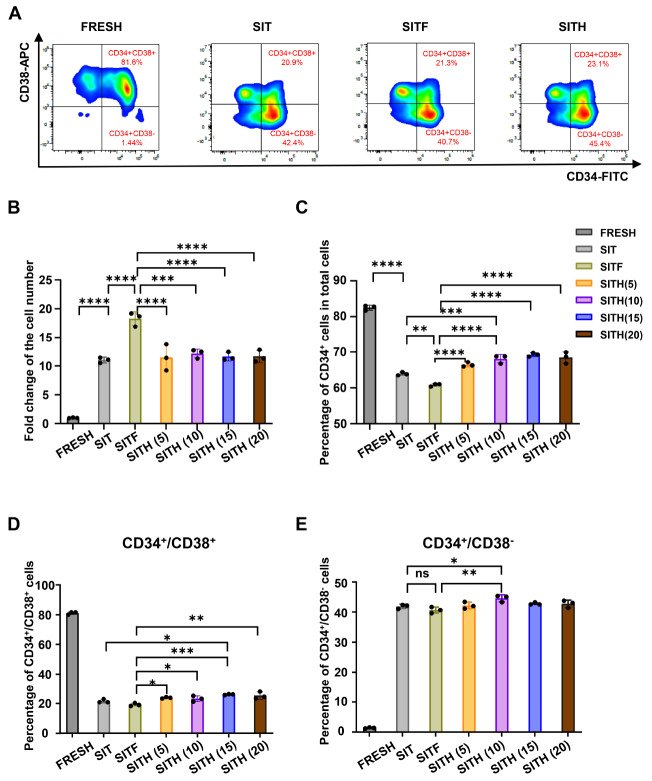
Figure 3 Expansion of UCB-CD34 ^+^ cells under different culture conditions (A) Flow cytometric analysis of CD34+/CD38+ and CD34+/CD38- cells under different culture conditions. (B) Total fold expansion of UCB-CD34 + cells under the indicated culture conditions. (C‒E) Flow cytometric analysis of the frequency of CD34+ cells (C), CD34+/CD38 + cells (D) and CD34+/CD38- cells (E) under the indicated culture conditions on days 0 and 7. FRESH represents CD34+ cells isolated from UCB on day 0, while SIT, SITF and SITH represent CD34+ cells under the indicated culture conditions on day 7 as described above, and SITH(5), SITH(10), SITH(15) and SITH(20) represent concentrations of the CH02 peptide of 5 ng/mL, 10 ng/mL, 15 ng/mL and 20 ng/mL, respectively. Data are shown as the mean±SD, n=3. * P<0.05, **P<0.01 and ***P<0.001; one-way ANOVA followed by Dunnett’s multiple comparison test.

To verify the potential impact of the CH02 peptide on UCB-HSPC proliferation, we isolated CD34 ^+^ cells from 10 UBCs with an average frequency of 65%±13%, including the CD34 ^+^/CD38 ^+^ committed progenitors (82%) and the CD34 ^+^/CD38 ^-^ multipotent counterpart (1.5%) ( Supplementary Figure S3 and Table S2). Based on a previous report [32], we took advantage of StemSpan medium supplemented with SCF, IL-6, TPO, and FLT3L to verify the potential role of the CH02-FLT3 axis in the *ex vivo* expansion of UCB-HSPCs. Since CH02 might have a similar function to FLT3L in activating the FLT3 signaling pathway, we substituted FLT3L with CH02 and performed 7-day culture in the indicated culture medium [SIT, SITF, SITH(5), SITH(10), SITH(15) and SITH(20)] ( Figure 3 ).

As shown in Figure 3B, the fold change of total UCB-HSPCs in the SITF group exhibited the highest value, which was 18- *fold* over that in the control group (denoted as FRESH). For the SITH groups with various concentrations of CH02, the fold changes in total UCB-HSPCs were moderately elevated, but the percentage of CD34 ^+^ cells was higher than that in the SITF group ( Figure 3B,C and Supplementary Figure S3B). Collectively, these results suggested that the CH02 peptide was more effective than FLT3L in promoting the proliferation and inhibiting the differentiation of CD34 ^+^ UCB-HSPCs.

In addition, we noticed that the frequencies of the CD34 ^+^/CD38 ^+^ subset consistently declined under all culture conditions ( Figure 3D and Supplementary Figure S3C‒E), whereas there was a higher ratio of CD34 ^+^/CD38 ^–^ multipotent progenitors ( Figure 3E and Supplementary Figure S3E). Interestingly, the frequencies of both CD34 ^+^/CD38 ^+^ and CD34 ^+^/CD38 ^–^ cells in the SITH (10) group were slightly higher than those in the SIT and SITF groups, respectively ( Figure 3C‒E). Taken together, these data suggested that the CH02 peptide was competent for the effective maintenance of stem cell properties as well as promoting *ex vivo* proliferation of UCB-HSPCs.

### The CH02 peptide promoted the proliferation of committed progenitors

**Figure 4:**
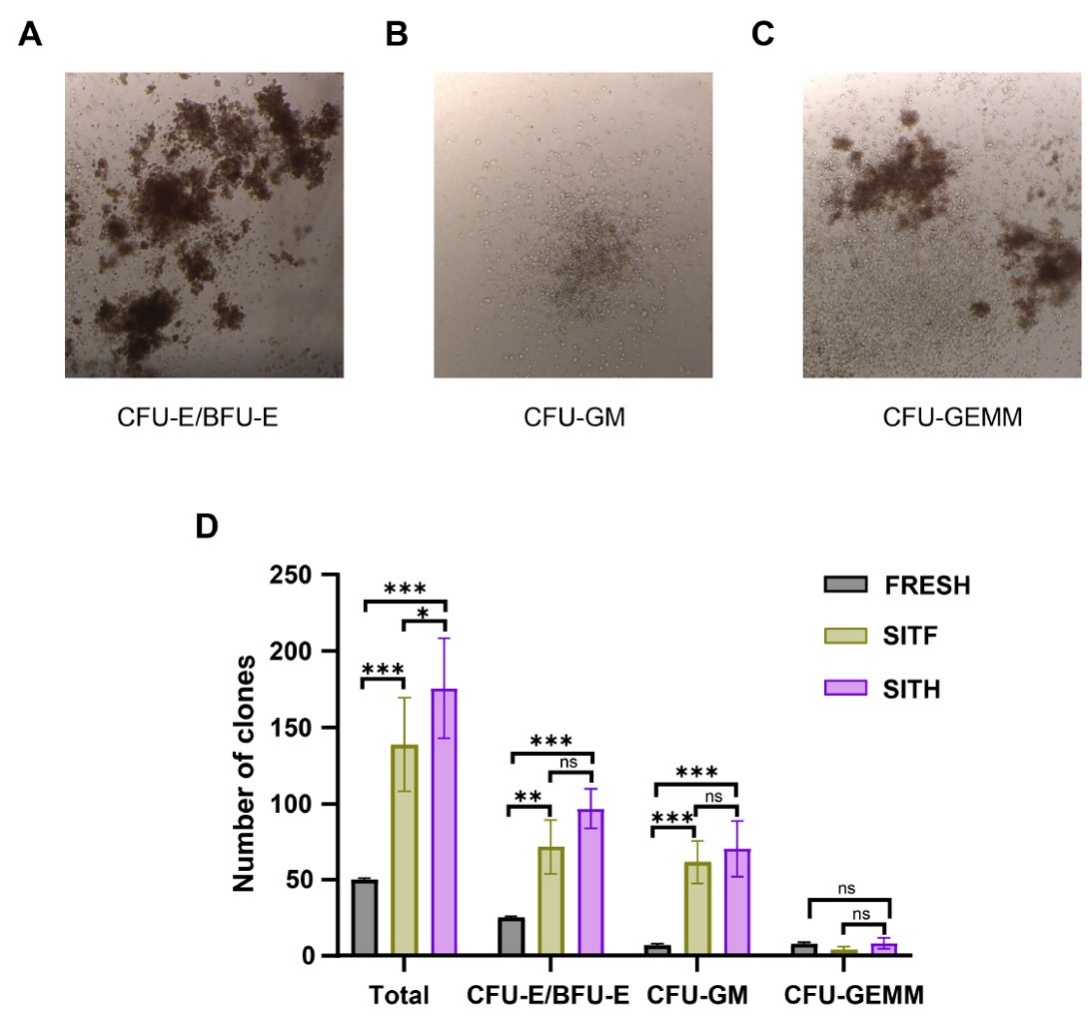
Figure 4 Expansion of committed progenitors under different culture conditions Morphology of CFU-E/BFU-E (A), CFU-GM (B) and CFU-GEMM (C) under different coculture conditions, and their expansion numbers (D) were measured under a microscope. CFU-E/BFU-E, colony-forming unit-erythroid/burst-forming unit-erythroid; CFU-GM, colony-forming unit-granulocyte/macrophage; CFU-GEMM, colony-forming unit-granulocyte/erythroid/macrophage/megakaryocyte. FRESH represents CD34+ cells isolated from UCB on day 0, while SIT, SITF and SITH represent CD34+ cells under the indicated culture conditions on day 7 as described above. Data are shown as the mean±SD, n=3. *P<0.05, ** P<0.01 and ***P<0.001; one-way ANOVA followed by Dunnett’s multiple comparison test.

HSPCs are capable of proliferation and differentiation into all blood cell types. To verify the potential influence of CH02 on the proliferation of committed progenitors, we conducted CFU assay and calculated the cloning numbers of the committed progenitors formed by the initial 1000 CD34 ^+^ cells under different culture conditions for 14 days ( Figure 4A‒C). Our results showed that the cloning numbers of CFU-GM and CFU-E/BFU-E under SITF and SITH culture conditions were significantly higher than their counterparts ( *P*< 0.01), while there was no significant difference ( *P* > 0.05) between these two groups ( Figure 4D). Taken together, these results suggested that the CH02 peptide could promote the proliferation of committed progenitors.

### The CH02 peptide facilitated the anti-inflammatory and growth-promoting properties of CD34 ^+^ UCB-HSPCs

**Figure 5:**
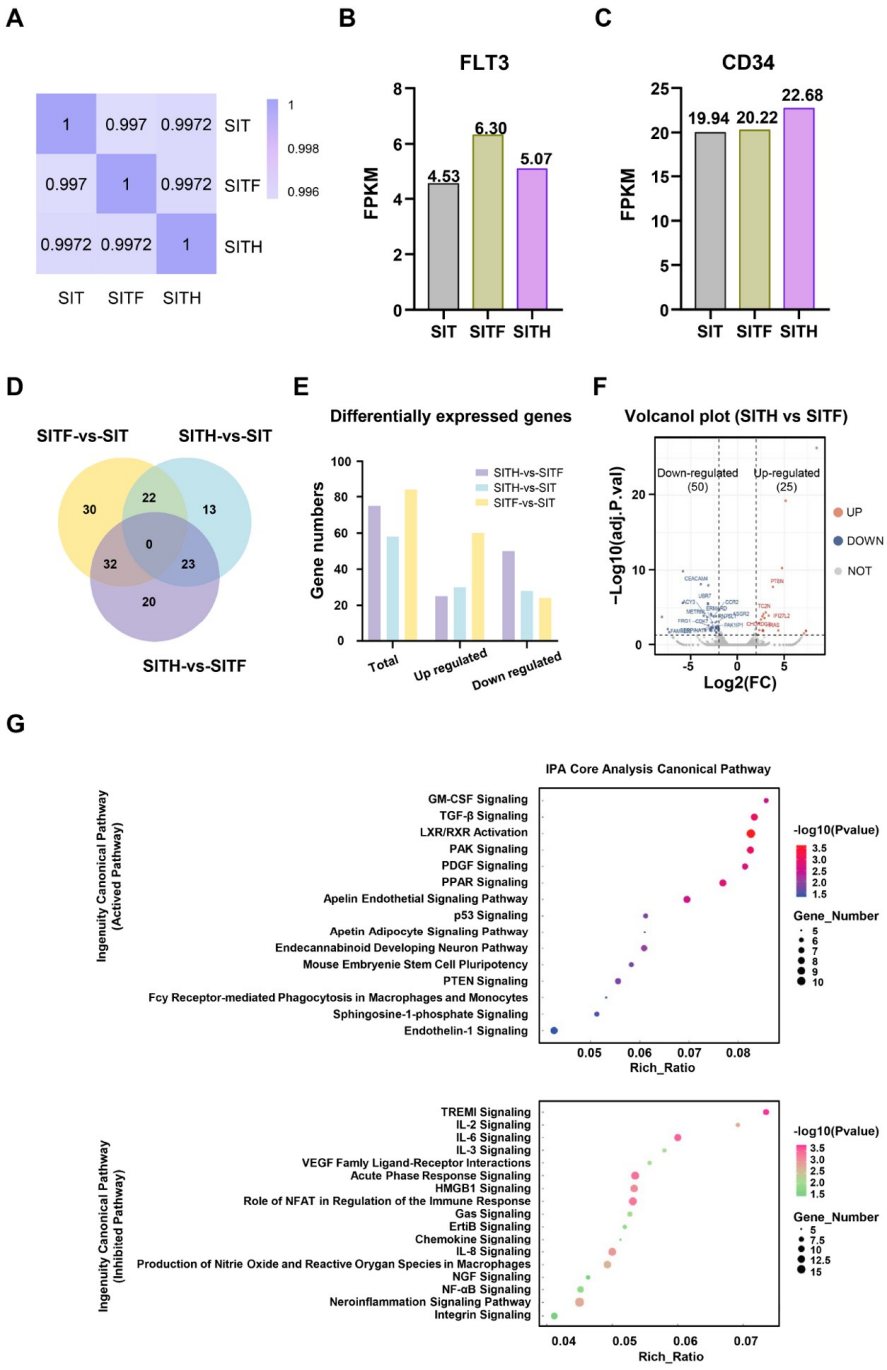
Figure 5 RNA-Seq analysis of UCB-CD34 ^+^ cells under different culture conditions (A) Pearson correlation coefficient of gene expression profiles of CD34+ cells under different culture conditions. SIT, SITF and SITH represent CD34+ cells under the indicated culture conditions on day 7 as described above. (B,C) RNA-Seq analysis of the expression of the FLT3 (B) and CD34 (C) genes in CD34 + cells under different culture conditions. The abscissa represents the indicated culture conditions, while the ordinate represents FPKM. (D,E) Venn diagram (D) and histogram (E) showing the numbers of differentially expressed genes between each pair of culture conditions. (F) Volcanol plot showing the differentially expressed genes between SITH- and SITF-cultured conditions. (G) Bubble chart of the differentially expressed genes between the SITF and SITH groups through IPA-KEGG signaling pathway analysis. The upper and lower charts show the predicted activation pathway and inhibitory pathway, respectively.

Having illuminated the impact of the CH02 peptide on the *ex vivo* expansion and maintenance of the multipotency of CD34 ^+^ cells, we further performed RNA-seq analysis to explore the potential similarities and differences of the aforementioned CD34 ^+^ UCB-HSPCs at the transcriptomic level. As shown by the Pearson correlation coefficient of gene expression profiles, we could intuitively notice the close association among UCB-HSPCs in the SIT, SITF and SITH groups ( Figure 5A). Furthermore, we analyzed the FPKM-based expression pattern of differentially expressed genes (DEGs) among the indicated groups and found that the SITF group and the SITH group showed the highest levels of FLT3 and CD34 expression, respectively ( Figure 5B,C). The relative expressions of HSPC markers ( *e*. *g*., CD49f and ESAM) in the SITH group was significantly higher than that in the SITF group, which suggested the impact of the CH02 peptide in maintaining the multipotent properties of CD34 ^+^ cells over FLT3L ( Supplementary Figure S4C,D).

In addition, based on the 72 DEGs between the SITH group and the SITF group ( Figure 5D‒F), we conducted IPA (Ingenuity Pathway Analysis) to verify the KEGG enrichment analysis and found that the upregulated DEGs in the SITH group were mainly enriched in anti-inflammatory and cell growth signaling pathways ( *e*. *g*., TGF-β signaling, PAK signaling, PDGF signaling and GM-CSF signaling) ( Figure 5G), which were mainly involved in wound healing. Instead, the downregulated DEGs were mainly enriched in inflammatory response-related signaling pathways such as IL-6 signaling, IL-8 signaling, and NF-κB signaling ( Figure 5G). Taken together, the transcriptomic data indicated that CD34 ^+^ UCB-HSPCs cultured under SITH condition (with CH02 addition) had better anti-inflammatory and growth-promoting properties than those cultured under SITF condition (without CH02 addition).

### The CH02 peptide increased the wound healing capacity of CD34 ^+^ UCB-HSPCs

**Figure 6:**
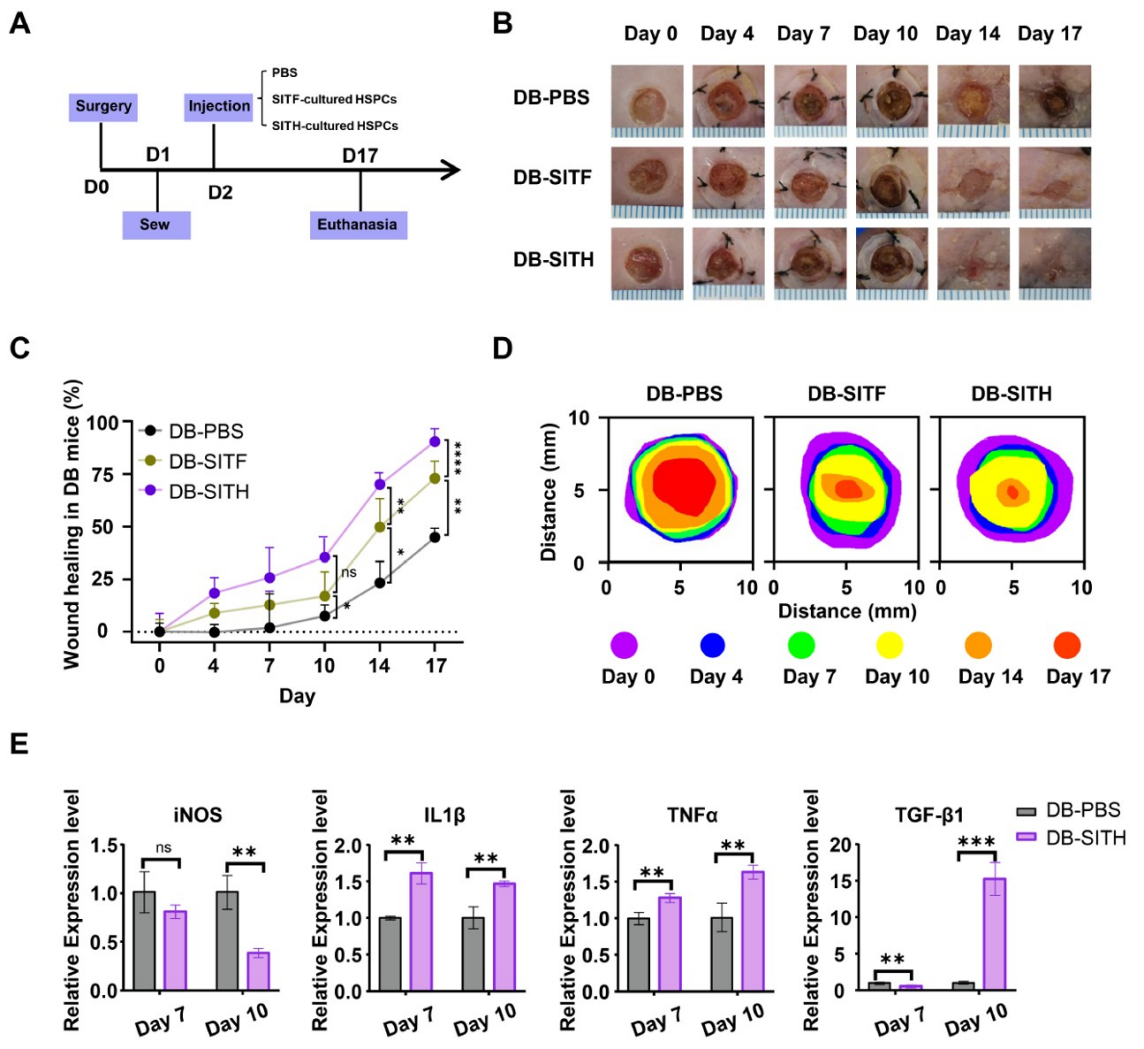
Figure 6 SITH-cultured CD34 ^+^ cells accelerated diabetic mouse wound healing (A) Scheme for transplantation of expanded CD34+ cells in a mouse skin ulcer model. (B) Injured mice received PBS or expanded CD34+ cells applied topically to the wounds. SITF or SITH represents CD34+ cells under the indicated culture conditions on day 7 as described above. DB, diabetic mice. (C) Topical application of expanded CD34+ cells in the SITH group ( n=5) resulted in faster wound closure than in the control group ( n=5) and SITF group (n=5). (D) Traces of wound-bed closure of the injured mice receiving PBS or expanded CD34+ cell transplantation. (E) Expression levels of inflammatory factors and anti-inflammatory factors in wounds on day 7 and day 10. Data are shown as the mean±SD, n=3. *P<0.05, **P<0.01, ***P<0.001 and ****P<0.001; one-way ANOVA followed by Dunnett’s multiple comparison test.

Previous studies have indicated that expanded CD34 ^+^ UCB-HSPCs are adequate to accelerate wound healing in diabetic mice [25]; thus, we took advantage of the diabetic skin ulcer db/db mouse model to compare the prorepair capacity of CD34 ^+^ cells after expansion under SITF and SITH culture conditions ( Figure 6A). As shown by the dynamic variations in skin ulcers, both wild-type (denoted as WT) and diabetic mice (denoted as DB) showed inflammation after the establishment of dorsal trauma in mice, while the inflammation in DB mice exhibited persistence until day 14, but WT mice showed alleviation after day 4 ( Supplementary Figure S5).

Compared with those mice with PBS administration (denoted as DB-PBS), DB mice with SITF- or SITH-preconditioned CD34 ^+^ UCB-HSPCs (denoted as DB-SITF and DB-SITH, respectively) were locally injected, the skin ulcers were efficiently alleviated, and the wound healing was largely accelerated ( Figure 6B‒D). Of note, mice in the DB-SITH group manifested preferable outcomes over those in the DB-SITF group, which indicated that CD34 ^+^ UCB-HSPCs preconditioned with CH02 peptide rather than FLT3 showed better efficacy in promoting wound healing in DB mice ( Figure 6B‒D). Consistently, the expression level of proinflammatory factors ( *e*. *g*., iNOS) was significantly reduced on day 10 after injection with the SITH-cultured UCB-CD34 ^+^ cells, while the expression levels of anti-inflammatory factors (IL-1β, TNF-α, and TGF-β1) were consistently increased compared to the DB-PBS group ( Figure 6E), which indicated the therapeutic effect of CH02 peptide-preconditioned CD34 ^+^ UCB-HSPCs on wound healing in diabetic mice after topical transplantation.

## Discussion

The proliferation and differentiation of HSPCs are the fundamental basis of hematopoiesis as well as the foundation of transplantation in patients with hematological malignancies. For decades, multiple approaches have been employed for *ex vivo* expansion of UCB-HSPCs by combination of specific media, cytokines, small molecules, bioreactors and even hematopoietic-supporting feeder cells ( *e*. *g*., mesenchymal stem/stromal cells). For instance, Ueda *et al* [33] reported that the combination of cytokines (SCF, FLT3L, TPO, IL-3, IL-6 and s-IL-6R) in 20% FBS-containing medium could expand UCB-CD34 ^+^ cells by 4.2-fold. Instead, Li *et al*. [7] took advantage of the SCF, FLT3L and TPO cocktails and efficiently expanded and maintained UCB-CD34 ^+^ cells by coculturing with the WJ-MSCs or UVECs feeder layer. In this study, we found that CH02 peptide and specific cocktail stimulation in serum-free medium was adequate to increase *ex vivo* expansion of CD34 ^+^ UCB-HSPCs by12- *fold* without impairing the multipotent property. Furthermore, with the aid of multifaceted transcriptomics analysis and *in vivo* transplantation assessment, we confirmed the superiority of CH02 peptide-preconditioned CD34 ^+^ UCB-HSPCs over FLT3-preconditioned cells in HSPC proliferation and wound healing management in diabetic mice.

FLT3 is a receptor tyrosine kinase (RTK) that is mainly expressed in the hematopoietic compartment [21]. FLT3 commonly interacts with FLT3L and activates the downstream PI3K and RAS pathways to orchestrate the growth of progenitor cells in the bone marrow microenvironment and peripheral blood [21]. In this study, based on the target prediction of CH02, we suspected that the CH02 peptide might have a similar function as FLT3L to promote the expansion of HSPCs by binding to FLT3 and activating the PI3K signaling cascades. Consistently, CD34 ^+^ cells under STIF and STIH conditions revealed highly similar patterns in the expression profiles. Meanwhile, we also noticed that FLT3 was more conducive for CD34 ^+^ UCB-HSPC expansion, whereas the CH02 peptide was more suitable for multipotency maintenance and *ex vivo* proliferation. Therefore, the CH02 peptide has the potential to serve as a novel substitute for FLT3L for cost-effective *ex vivo* expansion of CD34 ^+^ UCB-HSPCs in the future.

People with type 2 diabetes often suffer from chronic and nonhealing wounds. With the aid of the diabetic skin ulcer db/db mouse model, Whiteley *et al* [25] reported the wound healing and full thickness skin regeneration by *ex vivo* expanded CD34 ^+^ cells from frozen UCB. Herein, we found that CD34 ^+^ UCB-HSPCs under SITH rather than SITF stimulation for 7 days had preferable wound healing and skin regeneration abilities in db/db mice, which indicated the superiority of the CH02 peptide over FLT3L for *ex vivo* expansion of CD34 ^+^ cells. In addition, it has been shown that UCB-CD34 ^+^ cells can also serve as potential sources for endothelial precursor cell (EPC) and circulating angiogenic cell (CAC) preparation [34]. Due to the incompatibility between db/db mouse diabetic wounds and human donor cells, we speculated that the CH02-preconditioned CD34 ^+^ UCB-HSPCs might promote wound healing in *db*/ *db* mice via chemokine and cytokine secretion, which was confirmed by RNA-Seq data showing that SITH-preconditioned CD34 ^+^ cells were more effective for alleviating inflammation and benefiting cell growth than their SITF-preconditioned counterparts ( Figure 5G). Furthermore, we also confirmed the significant decrease in proinflammatory factor expression but increase in anti-inflammatory factor expression in wounds by UCB-CD34 ^+^ cell implantation, which further confirmed the aforementioned speculation. Interestingly, these observations were consistent with Whiteley’s report that UCB-CD34 ^+^ cells could promote tissue repair by secreting anti-inflammatory, pro-vascular and anti-apoptotic factors [6]. Of note, although the data in this study have demonstrated that HSPCs expanded by CH02-based cocktails in the replacement of FLT3L could benefit wound healing, we also realized the effect of these expanded cells on hematopoietic reconstruction after the first and second transplantation, which is the key for HSC expansion *ex vivo* rather than the core concerns in wound healing, and the administration should be further explored with systematic and detailed dissection in a follow-up study [ 35‒ 37]. Additionally, state-of-the-art renewal has also highlighted the feasibility of decoding specific subpopulations of HSPCs with unique functional tendentiousness and immune attributes, and thus, it will be of great interest to verify bipotential subsets and single-cell trajectories by combining them with surface marker candidates ( *e* . *g*., CD45RA, CD45RO, CD90 and CD49f) for better wound healing or rejection reaction administration during hematopoietic reconstitution or relative applications in the future [ 38‒ 40] .

In summary, our findings highlight the promising potential of the CH02 peptide in promoting UCB-HSPC *ex vivo* expansion by binding to FLT3 and activating the FLT3 signaling cascades. Moreover, topical transplantation of HSPCs accelerated the wound healing of diabetic mice, which indicated the preferable efficacy of CH02 peptide-preconditioned CD34 ^+^ UCB-HSPCs in clinical applications.

## Supplementary Data

Supplementary data is available at *Acta Biochimica et Biophysica Sinica* online.

## Supporting information

23018Supplementary_Table_S1

23018Supplementary_Table_S2

23018Supplementary_Table_S1-2

23018Supplementary_Figures
