# Supplementary material for: CH02 peptide promotes ex vivo expansion of umbilical cord blood-derived CD34 + hematopoietic stem/progenitor cells : CH02 peptide promotes CD34 + UCB-HSPC ex vivo expansion
Source: Acta Biochim Biophys Sin (Shanghai). 2023 Jun 28;55(10):1630–9. doi: 10.3724/abbs.2023047 (PMC10577473; doi:10.3724/abbs.2023047)
Supplement: 23018Supplementary_Table_S1 [file 23018Supplementary_Table_S1.pdf]

Protein	Protein.Name	Gene.Nam	Mol.weigh	Sequence.	Proteins	Positions.v	Fasta.heac
Q9JKS6	Protein piccolo	Pclo	552.71	5085	Q9JKS6	2685	PCLO_RAT
B5DF41	Syntaphilin	Snph	54.493	504	B5DF41	200	SNPH_RAT
Q9JI66	Electrogenic sodium bica	Slc4a4	121.34	1079	Q9JI66	1069	S4A4_RAT
P09760	Tyrosine-protein kinase F	Fer	94.313	823	P09760	45	FER_RAT T
P09760	Tyrosine-protein kinase F	Fer	94.313	823	P09760	44	FER_RAT T
P09760	Tyrosine-protein kinase F	Fer	94.313	823	P09760	42	FER_RAT T
Q9JI66	Electrogenic sodium bica	Slc4a4	121.34	1079	Q9JI66;Q6	223;226	S4A4_RAT
Q5XFX0	Transgelin-2	Tagln2	22.393	199	Q5XFX0	163	TAGL2_RA
O88923	Adhesion G protein-coup	Adgrl2	166.73	1487	O88923	1458	AGRL2_RA
Q66HA4	Tax1-binding protein 1 h	Tax1bp1	93.196	813	Q66HA4	494	TAXB1_RA
Q0V8T4	Contactin-associated pro	Cntnap5c	145.72	1307	Q0V8T4	921	CTP5C_RA
Q4L0E8	Regulator of G-protein si	Rgs18	27.644	235	Q4L0E8	220	RGS18_RA
Q4L0E8	Regulator of G-protein si	Rgs18	27.644	235	Q4L0E8	221	RGS18_RA
Q08013	Translocon-associated pr	Ssr3	21.064	185	Q08013	105	SSRG_RAT
P11884	Aldehyde dehydrogenase	Aldh2	56.488	519	P11884	8	ALDH2_RA
Q5BK81	Prostaglandin reductase 2	Ptgr2	38.136	351	Q5BK81	350	PTGR2_RA
Q3B8Q0	Microtubule-associated p	Mapre2	36.988	326	Q3B8Q0	231	MARE2_RA
Q6AY91	Nicotinamide riboside kir	Nmrk1	22.321	195	Q6AY91	116	NRK1_RAT
Q6AY91	Nicotinamide riboside kir	Nmrk1	22.321	195	Q6AY91	110	NRK1_RAT
Q6AY91	Nicotinamide riboside kir	Nmrk1	22.321	195	Q6AY91	119	NRK1_RAT
Q6AY91	Nicotinamide riboside kir	Nmrk1	22.321	195	Q6AY91	115	NRK1_RAT
P62859	40S ribosomal protein S2	Rps28	7.8409	69	P62859	23	RS28_RAT
G3V928	Prolow-density lipoprotei	Lrp1	504.88	4545	G3V928	4521	LRP1_RAT
Q62901	Arginine-glutamic acid di	Rere	171.81	1559	Q62901	655	RERE_RAT
P60203	Myelin proteolipid protei	Plp1	30.077	277	P60203	118	MYPR_RAT
P59649	FXYD domain-containing	Fxyd7	8.4867	80	P59649	60	FXYD7_RA
Q4V888	Type 2 phosphatidylinosi	Pip4p2	28.024	257	Q4V888	89	PP4P2_RA
P68403	Protein kinase C beta typ	Prkcb	76.75	671	P68403;P6	500;514	KPCB_RAT
O09032	ELAV-like protein 4	Elavl4	42.368	385	O09032	42	ELAV4_RA
Q3ZB98	Breast carcinoma-amplifi	Bcas1	62.381	589	Q3ZB98	351	BCAS1_RA
Q4V8J4	Glycerol-3-phosphate ac	Gpat3	51.011	457	Q4V8J4	65	GPAT3_RA
A0A0G2K2	Tight junction protein ZO	Tjp1	197.15	1765	A0A0G2K2	212	ZO1_RAT
P30009	Myristoylated alanine-ricl	Marcks	29.794	309	P30009	26	MARCS_RA
D3ZGB1	Nuclear factor of activate	Nfat5	167.21	1548	D3ZGB1	1020	NFAT5_RA
D3ZGB1	Nuclear factor of activate	Nfat5	167.21	1548	D3ZGB1	1040	NFAT5_RA
P29995	Inositol 1,4,5-trisphospha	Itpr2	307.05	2701	P29995	1855	ITPR2_RAT
P63059	Thyroid hormone recepto	Thra	55.071	492	P63059	273	THA_RAT
P63059	Thyroid hormone recepto	Thra	55.071	492	P63059	275	THA_RAT
P63059	Thyroid hormone recepto	Thra	55.071	492	P63059	267	THA_RAT
Q8VGC3	Voltage-dependent L-typ	Cacnb2	73.225	655	Q8VGC3	501	CACB2_RA
F1LP64	E3 ubiquitin-protein ligas	Trip12	223.93	2025	F1LP64	238	TRIPC_RAT
P60669	Pleckstrin homology dom	Plekha4	85.641	779	P60669	164	PKHA4_RA
P06907	Myelin protein P0	Mpz	27.57	248	P06907	206	MYP0_RAT
Q5RKH1	Serine/threonine-protein	Prpf4b	117.01	1007	Q5RKH1	427	PRP4B_RA
O09032	ELAV-like protein 4	Elavl4	42.368	385	O09032	38	ELAV4_RA
Q75PQ8	Origin recognition compl	Orc2	65.849	576	Q75PQ8	3	ORC2_RAT
Q62737	Cytochrome b-245 light c	Cyba	20.75	192	Q62737	147	CY24A_RA
P42930	Heat shock protein beta-	Hspb1	22.892	206	P42930	90	HSPB1_RA
Q5XII9	Mitochondrial fission regu	Mtfr1l	31.73	289	Q5XII9	38	MFR1L_RA
O35828	Coronin-7	Coro7	100.78	922	O35828	459	CORO7_RA
Q5M7V8	Thyroid hormone recepto	Thrap3	108.25	951	Q5M7V8	238	TR150_RA
Q9EPA0	Dystrophin-related prote	Drp2	108.04	957	Q9EPA0	104	DRP2_RAT
Q9JKU0	Taste receptor type 2 mei	Tas2r16	34.641	299	Q9JKU0	214	T2R16_RA
P84903	Stromal interaction molec	Stim1	77.448	685	P84903	257	STIM1_RA
Q03555	Gephyrin	Gphn	83.265	768	Q03555	327	GEPH_RAT
Q03555	Gephyrin	Gphn	83.265	768	Q03555	326	GEPH_RAT
O35430	Amyloid-beta A4 precurs	Apba1	92.653	839	O35430	570	APBA1_RA

Q99P82	Claudin-11	Cldn11	22.046	207	Q99P82	197	CLD11_RA
Q9R1N3	Sodium bicarbonate cotra	Slc4a7	135.95	1218	Q9R1N3;Q	238;238	S4A7_RAT
Q499S9	Inactive rhomboid protei	Rhbdf1	97.261	856	Q499S9	49	RHDF1_RA
O08875	Serine/threonine-protein	Dclk1	47.68	433	O08875	45	DCLK1_RA
Q4G008	Uncharacterized protein KIAA0930 h		45.958	404	Q4G008	270	K0930_RAT
O35052	Phosphatidate cytidylyltra	Cds1	52.969	461	O35052	18	CDS1_RAT
Q9Z1Z1	Eukaryotic translation init	Eif2ak3	124.77	1108	Q9Z1Z1	547	E2AK3_RA
Q6AYA6	Cytochrome b-245 chape	Cybc1	20.894	187	Q6AYA6	168	CYBC1_RA
P63149	Ubiquitin-conjugating en	Ube2b	17.312	152	P63149	3	UBE2B_RA
Q9QZ86	Nucleolar protein 58	Nop58	60.07	534	Q9QZ86	507	NOP58_RA
Q9JHY1	Junctional adhesion mole	F11r	32.369	300	Q9JHY1	285	JAM1_RAT
O35814	Stress-induced-phospho	Stip1	62.569	543	O35814	481	STIP1_RAT
O35814	Stress-induced-phospho	Stip1	62.569	543	O35814	476	STIP1_RAT
Q5FVJ6	Seipin	Bscl2	42.409	377	Q5FVJ6	280	BSCL2_RA
Q63135	Complement component	Cr1l	61.68	559	Q63135	554	CR1L_RAT
Q5RKH0	Putative oxidoreductase (	Glyr1	60.421	552	Q5RKH0	166	GLYR1_RA
P06907	Myelin protein P0	Mpz	27.57	248	P06907	205	MYP0_RAT
Q9QZM5	Abl interactor 1	Abi1	51.704	476	Q9QZM5	224	ABI1_RAT
P52796	Ephrin-B1	Efnb1	37.951	345	P52796	282	EFNB1_RA
Q63269	Inositol 1,4,5-trisphospha	Itpr3	304.28	2670	Q63269	2669	ITPR3_RAT
P61314	60S ribosomal protein L1	Rpl15	24.146	204	P61314	34	RL15_RAT
Q9JLH5	CDK5 regulatory subunit-	Cdk5rap2	215.48	1903	Q9JLH5	1896	CK5P2_RA
Q9R1N3	Sodium bicarbonate cotra	Slc4a7	135.95	1218	Q9R1N3	1180	S4A7_RAT
P08050	Gap junction alpha-1 pro	Gja1	43.031	382	P08050	306	CXA1_RAT
P34900	Syndecan-2	Sdc2	22.149	201	P34900	187	SDC2_RAT
Q5M7V8	Thyroid hormone recepto	Thrap3	108.25	951	Q5M7V8	532	TR150_RA
Q99P82	Claudin-11	Cldn11	22.046	207	Q99P82	196	CLD11_RA
Q9Z327	Synaptopodin	Synpo	99.985	931	Q9Z327	134	SYNPO_RA
Q9JK71	Membrane-associated gu	Magi3	160.56	1470	Q9JK71	954	MAGI3_RA
Q3ZB98	Breast carcinoma-amplifi	Bcas1	62.381	589	Q3ZB98	547	BCAS1_RA
Q5FVI4	Cell cycle exit and neuror	Cend1	15.043	149	Q5FVI4	9	CEND_RAT
Q9EPH2	MARCKS-related protein	Marcksl1	19.847	199	Q9EPH2	41	MRP_RAT
Q923J6	Dynein heavy chain 12, a	Dnah12	357.24	3092	Q923J6	2307	DYH12_RA
Q923J6	Dynein heavy chain 12, a	Dnah12	357.24	3092	Q923J6	2305	DYH12_RA
Q62901	Arginine-glutamic acid di	Rere	171.81	1559	Q62901	612	RERE_RAT
P52796	Ephrin-B1	Efnb1	37.951	345	P52796	280	EFNB1_RA
P16443	D site-binding protein	Dbp	34.436	325	P16443	164	DBP_RAT
Q5FVI4	Cell cycle exit and neuror	Cend1	15.043	149	Q5FVI4	15	CEND_RAT
P06765	Platelet factor 4	Pf4	11.286	105	P06765	34	PLF4_RAT
P31596	Excitatory amino acid trar	Slc1a2	62.106	573	P31596	520	EAA2_RAT
P18266	Glycogen synthase kinase	Gsk3a	46.742	420	P18265;P1	279;216	GSK3A_RA
P29975	Aquaporin-1	Aqp1	28.856	269	P29975	247	AQP1_RAT
Q8K4S7	E3 ubiquitin-protein ligas	Cblb	104.65	938	Q8K4S7	527	CBLB_RAT
Q9WVC0	Septin-7	Septin7	50.507	436	Q9WVC0	425	SEPT7_RA
Q9Z2Q7	Syntaxin-8	Stx8	26.91	236	Q9Z2Q7	102	STX8_RAT
A6YP92	Homeobox protein ARX	Arx	58.646	566	A6YP92	22	ARX_RAT
P60203	Myelin proteolipid protei	Plp1	30.077	277	P60203	114	MYPR_RAT
Q9JI66	Electrogenic sodium bica	Slc4a4	121.34	1079	Q9JI66	257	S4A4_RAT
P41123	60S ribosomal protein L1	Rpl13	24.309	211	P41123	139	RL13_RAT
P52591	Nuclear envelope pore m	Pom121	120.78	1199	P52591	421	PO121_RA
Q498U0	Uncharacterized protein C4orf3 hon		7.3703	65	Q498U0	26	CD003_RA
P06907	Myelin protein P0	Mpz	27.57	248	P06907	195	MYP0_RAT
Q5EXX3	Zinc finger and BTB dom	Zbtb38	135.3	1203	Q5EXX3	130	ZBT38_RA
Q810W7	Microtubule-associated s	Mast1	171.03	1570	Q810W7	1268	MAST1_RA
Q0KL00	Piezo-type mechanosens	Piezo1	290.31	2535	Q0KL00	1633	PIEZ1_RAT
Q5EXX3	Zinc finger and BTB dom	Zbtb38	135.3	1203	Q5EXX3	939	ZBT38_RA
Q64649	Phosphorylase b kinase re	Phka1	139.15	1242	Q64649	879	KPB1_RAT
Q64649	Phosphorylase b kinase re	Phka1	139.15	1242	Q64649	875	KPB1_RAT

Q64649	Phosphorylase b kinase re	Phka1	139.15	1242	Q64649	870	KPB1_RAT
Q63796	Mitogen-activated protei	Map3k12	96.306	888	Q63796	569	M3K12_RA
P15146	Microtubule-associated p	Map2	202.41	1861	P15146	1541	MTAP2_RA
P59649	FXYD domain-containing	Fxyd7	8.4867	80	P59649	56	FXYD7_RA
P59649	FXYD domain-containing	Fxyd7	8.4867	80	P59649	58	FXYD7_RA
P15146	Microtubule-associated p	Map2	202.41	1861	P15146	1539	MTAP2_RA
Q2THW7	Palmitoyltransferase ZDH	Zdhhc5	77.429	715	Q2THW7	380	ZDHC5_RA
P06907	Myelin protein P0	Mpz	27.57	248	P06907	229	MYP0_RAT
O08678	Serine/threonine-protein	Mark1	88.234	793	O08678;O	215;208;2	MARK1_RA
Q6AYJ1	ATP-dependent DNA hel	Recql	69.641	621	Q6AYJ1	602	RECQ1_RA
Q02294	Voltage-dependent N-ty	Cacna1b	262.25	2336	Q02294	921	CAC1B_RA
Q63488	Sodium-dependent phos	Slc20a2	70.747	656	Q63488	259	S20A2_RA
Q62649	Neuronatin	Nnat	9.2197	81	Q62649	56	NNAT_RAT
P00564	Creatine kinase M-type	Ckm	43.044	381	P00564	372	KCRM_RAT
Q5JCS6	Signal-induced proliferat	Sipa1l2	189.65	1722	Q5JCS6	1353	SI1L2_RAT
Q5BJP5	Transmembrane protein 2	Tmem230	13.204	120	Q5BJP5	24	TM230_RA
O35413	Sorbin and SH3 domain-	Sorbs2	134.06	1196	O35413	1113	SRBS2_RA
P15865	Histone H1.5	H1-5	21.987	219	D3ZBN0;P	103;104;1	H15_RAT I
Q9WVC0	Septin-7	Septin7	50.507	436	Q9WVC0	333	SEPT7_RAT
P26684	Endothelin-1 receptor	Ednra	48.244	426	P26684	424	EDNRA_RA
Q6P767	Pituitary tumor-transform	Pttg1ip	19.9	174	Q6P767	171	PTTG_RAT
P70615	Lamin-B1	Lmnb1	66.606	587	P70615	576	LMNB1_RA
Q9R066	Coxsackievirus and adeno	Cxadr	39.948	365	Q9R066	323	CXAR_RAT
P06907	Myelin protein P0	Mpz	27.57	248	P06907	228	MYP0_RAT
Q6AXY7	Pre-mRNA-splicing facto	Prpf38b	63.931	542	Q6AXY7	525	PR38B_RA
Q62671	E3 ubiquitin-protein ligas	Ubr5	308.02	2788	Q62671	970	UBR5_RAT
Q6URK4	Heterogeneous nuclear ri	Hnrnpa3	39.652	379	Q6URK4	359	ROA3_RAT
P60203	Myelin proteolipid protei	Plp1	30.077	277	P60203	116	MYPR_RAT
Q63312	Pleckstrin homology-like	Phldb1	93.54	831	Q63312	14	PHLB1_RA
Q62636	Ras-related protein Rap-	Rap1b	20.798	184	Q62636;P	39;39	RAP1B_RA
D3ZFB6	Proline-rich transmembra	Prrt2	35.82	344	D3ZFB6	242	PRRT2_RA
Q8R4S8	Cytokine receptor-like fa	Crlf2	38.445	360	Q8R4S8	278	CRLF2_RA
Q5RKH0	Putative oxidoreductase (	Glyr1	60.421	552	Q5RKH0	130	GLYR1_RA
Q9JI66	Electrogenic sodium bica	Slc4a4	121.34	1079	Q9JI66	256	S4A4_RAT
P31211	Corticosteroid-binding gl	Serpina6	44.67	396	P31211	292	CBG_RAT
P31211	Corticosteroid-binding gl	Serpina6	44.67	396	P31211	285	CBG_RAT
Q62812	Myosin-9	Myh9	226.34	1961	Q62812	1940	MYH9_RAT
P19814	Trans-Golgi network inte	Ttgn1	38.304	357	P19814	234	TGON3_RA
D3ZHA0	Filamin-C	Flnc	290.98	2726	D3ZHA0	2625	FLNC_RAT
D3ZXD8	Transmembrane protein 2	Tmem245	97.281	876	D3ZXD8	12	TM245_RA
Q3T1J9	MOB kinase activator 1A	Mob1a	25.079	216	Q3T1J9	35	MOB1A_R
P19527	Neurofilament light polyp	Nefl	61.335	542	P19527	44	NFL_RAT N
P19332	Microtubule-associated p	Mapt	78.563	752	P19332	574	TAU_RAT
P85125	Caveolae-associated prot	Cavin1	43.908	392	P85125	173	CAVN1_RA
P85125	Caveolae-associated prot	Cavin1	43.908	392	P85125	177	CAVN1_RA
Q63269	Inositol 1,4,5-trisphospha	Itpr3	304.28	2670	Q63269	1842	ITPR3_RAT
Q569C0	Transmembrane protein 1	Tmem100	14.352	134	Q569C0	121	TM100_RA
P02688	Myelin basic protein	Mbp	21.502	195	P02688	94	MBP_RAT
O55035	Peptidyl-prolyl cis-trans i	Ppig	88.378	752	O55035	395	PPIG_RAT
F1LP64	E3 ubiquitin-protein ligas	Trip12	223.93	2025	F1LP64	241	TRIPC_RAT
Q62639	GTP-binding protein Rhe	Rheb	20.479	184	Q62639	175	RHEB_RAT
Q63356	Unconventional myosin-I	Myo1e	126.83	1107	Q63356	1001	MYO1E_RA
P61150	Fibroblast growth factor 1	Fgf12	27.399	243	P61150	212	FGF12_RA
P48679	Prelamin-A/C	Lmna	74.323	665	P48679	403	LMNA_RA
O08961	Zinc finger protein 423	Znf423	147.23	1311	O08961	1186	ZN423_RA
P06907	Myelin protein P0	Mpz	27.57	248	P06907	209	MYP0_RAT
P06907	Myelin protein P0	Mpz	27.57	248	P06907	120	MYP0_RAT
P06907	Myelin protein P0	Mpz	27.57	248	P06907	124	MYP0_RAT

P06907	Myelin protein P0	Mpz	27.57	248	P06907		126	MYP0_RAT
Q5RKH1	Serine/threonine-protein	Prpf4b	117.01	1007	Q5RKH1		258	PRP4B_RA
P97573	Phosphatidylinositol 3,4,5	Inpp5d	133.59	1190	P97573		245	SHIP1_RAT
P32738	Choline O-acetyltransfera	Chat	71.863	640	P32738		365	CLAT_RAT
F1M3G7	A-kinase anchor protein 1	Akap13	301.4	2760	F1M3G7		765	AKP13_RA
O54921	Exocyst complex compon	Exoc2	104.03	924	O54921		432	EXOC2_RA
Q5RJQ4	NAD-dependent protein	Sirt2	39.319	350	Q5RJQ4		330	SIR2_RAT
Q9EPH2	MARCKS-related protein	Marcksl1	19.847	199	Q9EPH2		22	MRP_RAT
Q65Z14	Oncostatin-M-specific re	Osmr	108.62	962	Q65Z14		782	OSMR_RA
Q8VHK2	Caskin-1	Caskin1	150.35	1430	Q8VHK2		1035	CSKI1_RAT
P63326	40S ribosomal protein S1	Rps10	18.916	165	P63326		146	RS10_RAT
P06907	Myelin protein P0	Mpz	27.57	248	P06907		210	MYP0_RAT
Q63358	Unconventional myosin-I	Myo9b	225.03	1980	Q63358		1222	MYO9B_R
Q9WU74	Lipolysis-stimulated lipop	Lsr	65.776	593	Q9WU74		325	LSR_RAT L
Q9R1Q2	Cyclin-L1	Ccnl1	59.79	527	Q9R1Q2		353	CCNL1_RA
Q8R4T5	General receptor for phos	Grasp	42.32	394	Q8R4T5		93	GRASP_RA
Q6AYS6	Sorting nexin-17	Snx17	52.882	470	Q6AYS6		409	SNX17_RA
O89000	Dihydropyrimidine dehyd	Dpyd	111.47	1025	O89000		905	DPYD_RAT
Q499S9	Inactive rhomboid protein	Rhbdf1	97.261	856	Q499S9		391	RHDF1_RA
Q62733	Lamina-associated polyp	Tmpo	50.277	452	Q62733		157	LAP2_RAT
O08984	Delta(14)-sterol reductase	Lbr	70.723	620	O08984		70	LBR_RAT D
Q8K1P7	Transcription activator BR	Smarca4	181.43	1613	Q8K1P7		1349	SMCA4_RA
P15865	Histone H1.4	H1-4	21.987	219	P15865		146	H14_RAT H
P02688	Myelin basic protein	Mbp	21.502	195	P02688		72	MBP_RAT
G3V6S8	Serine/arginine-rich splic	Srsf6	39.025	339	G3V6S8		316	SRSF6_RAT
Q9WU74	Lipolysis-stimulated lipop	Lsr	65.776	593	Q9WU74		454	LSR_RAT L
Q63624	Splicing factor, arginine/s	Scaf1	133.85	1258	Q63624		678	SFR19_RAT
Q9WUD2	Transient receptor potent	Trpv2	86.705	761	Q9WUD2		46	TRPV2_RA
F1LP90	Misshapen-like kinase 1	Mink1	150.38	1336	F1LP90		602	MINK1_RA
Q91ZQ0	Vacuole membrane prote	Vmp1	45.9	406	Q91ZQ0		23	VMP1_RAT
P34900	Syndecan-2	Sdc2	22.149	201	P34900		188	SDC2_RAT
P26434	Sodium/hydrogen exchar	Slc9a4	81.522	717	P26434;P4	545;555		SL9A4_RAT
Q3KR59	Ubiquitin carboxyl-termin	Usp10	87.31	794	Q3KR59		361	UBP10_RA
O55164	Multiple PDZ domain pro	Mpdz	218.59	2054	O55164		798	MPDZ_RA
P60669	Pleckstrin homology dom	Plekha4	85.641	779	P60669		627	PKHA4_RA
P50878	60S ribosomal protein L4	Rpl4	47.256	421	P50878		295	RL4_RAT 6
Q63425	Periaxin	Prx	146.4	1383	Q63425		1312	PRAX_RAT
Q5BJT0	Arginine and glutamate-r	Arglu1	32.887	271	Q5BJT0		75	ARGL1_RA
O08589	Phospholemman	Fxyd1	10.365	92	O08589		79	PLM_RAT
P22909	Alpha-2A adrenergic rece	Adra2a	48.939	450	P22909		345	ADA2A_RA
P84092	AP-2 complex subunit mu	Ap2m1	49.654	435	P84092		236	AP2M1_RA
P08050	Gap junction alpha-1 pro	Gja1	43.031	382	P08050		372	CXA1_RAT
Q9JJS5	SH3 domain-binding pro	Sh3bp4	107.51	961	Q9JJS5		246	SH3B4_RA
D4A631	Brefeldin A-inhibited gua	Arfgef1	207.89	1846	D4A631		1076	BIG1_RAT
D3ZQL6	MICAL-like protein 1	Micall1	92.643	855	D3ZQL6		30	MILK1_RAT
P60669	Pleckstrin homology dom	Plekha4	85.641	779	P60669		577	PKHA4_RA
O55035	Peptidyl-prolyl cis-trans i	Ppig	88.378	752	O55035		356	PPIG_RAT
D3ZXD8	Transmembrane protein 2	Tmem245	97.281	876	D3ZXD8		16	TM245_RA
P97546	Neuroplastin	Nptn	43.931	393	P97546		379	NPTN_RAT
P0C5E3	Palladin (Fragment)	Palld	66.704	603	P0C5E3		170	PALLD_RA
Q4KM98	Mitochondrial fission fact	Mff	24.97	218	Q4KM98		131	MFF_RAT
Q5RKH1	Serine/threonine-protein	Prpf4b	117.01	1007	Q5RKH1		387	PRP4B_RA
P35570	Insulin receptor substrate	Irs1	131.18	1235	P35570		789	IRS1_RAT
P05197	Elongation factor 2	Eef2	95.283	858	P05197		57	EF2_RAT E
O70511	Ankyrin-3	Ank3	284.44	2622	O70511		2216	ANK3_RAT
P02688	Myelin basic protein	Mbp	21.502	195	P02688		41	MBP_RAT
P61980	Heterogeneous nuclear ri	Hnrnpk	50.976	463	P61980		379	HNRPK_RA
P26453	Basigin	Bsg	42.435	388	P26453		371	BASI_RAT

P48679	Prelamin-A/C	Lmna	74.323	665	P48679	404	LMNA_RA
Q812D1	PC4 and SFRS1-interactir	Psip1	59.638	528	Q812D1	207	PSIP1_RAT
Q642G4	Peroxisomal membrane p	Pex14	40.936	376	Q642G4	282	PEX14_RA
D3ZAR1	Low density lipoprotein r	Ldlrap1	33.785	306	D3ZAR1	197	ARH_RAT
O35786	Chemokine-like receptor	Cmklr1	41.722	371	O35786	356	CML1_RAT
Q9R050	Single-stranded DNA-bir	Ssbp3	37.714	361	Q9R050	320	SSBP3_RA
P08050	Gap junction alpha-1 pro	Gja1	43.031	382	P08050	328	CXA1_RAT
Q921A3	Ubiquitin D	Ubd	17.983	161	Q921A3	107	UBD_RAT
P48679	Prelamin-A/C	Lmna	74.323	665	P48679	414	LMNA_RA
Q9ERW3	Fibroblast growth factor 1	Fgf13	27.587	245	Q9ERW3	222	FGF13_RA
F1LP64	E3 ubiquitin-protein ligas	Trip12	223.93	2025	F1LP64	77	TRIPC_RAT
D3ZXD8	Transmembrane protein 2	Tmem245	97.281	876	D3ZXD8	329	TM245_RA
O35786	Chemokine-like receptor	Cmklr1	41.722	371	O35786	355	CML1_RAT
E9PTG8	Serine/threonine-protein	Stk10	111.88	967	E9PTG8	514	STK10_RAT
Q9JKS6	Protein piccolo	Pclo	552.71	5085	Q9JKS6	617	PCLO_RAT
P19332	Microtubule-associated p	Mapt	78.563	752	P19332	573	TAU_RAT
Q62952	Dihydropyrimidinase-rela	Dpysl3	61.967	570	Q62952	514	DPYL3_RA
Q62733	Lamina-associated polyp	Tmpo	50.277	452	Q62733	384	LAP2_RAT
P97528	Contactin-6	Cntn6	114.06	1028	P97528	87	CNTN6_RA
Q5M7V8	Thyroid hormone recepto	Thrap3	108.25	951	Q5M7V8	320	TR150_RAT
P48679	Prelamin-A/C	Lmna	74.323	665	P48679	573	LMNA_RA
P11497	Acetyl-CoA carboxylase 1	Acaca	265.19	2345	P11497	79	ACACA_RA
P11345	RAF proto-oncogene seri	Raf1	72.927	648	P11345;P1	621;580	RAF1_RAT
Q63433	Serine/threonine-protein	Pkn1	104.47	946	Q63433	377	PKN1_RAT
P97710	Tyrosine-protein phosph	Sirpa	55.69	509	P97710	409	SHPS1_RA
B3DMA0	Tumor protein p53-induc	Tp53i11	20.939	189	B3DMA0	14	P5I11_RAT
Q99P82	Claudin-11	Cldn11	22.046	207	Q99P82	194	CLD11_RA
Q9WUD2	Transient receptor potent	Trpv2	86.705	761	Q9WUD2	47	TRPV2_RA
Q9QZ48	Zinc finger and BTB doma	Zbtb7a	60.543	569	Q9QZ48	331	ZBT7A_RA
P62243	40S ribosomal protein S8	Rps8	24.205	208	P62243	130	RS8_RAT 4
P49793	Nuclear pore complex pro	Nup98	197.28	1816	P49793	888	NUP98_RA
Q5JCS6	Signal-induced proliferat	Sipa1l2	189.65	1722	Q5JCS6	1461	SI1L2_RAT
P13596	Neural cell adhesion mol	Ncam1	94.657	858	P13596	798	NCAM1_R
P02688	Myelin basic protein	Mbp	21.502	195	P02688	68	MBP_RAT
Q5XIS8	Mitochondrial dynamics p	Mief1	51.309	463	Q5XIS8	59	MID51_RA
Q9JHY1	Junctional adhesion mole	F11r	32.369	300	Q9JHY1	288	JAM1_RAT
P62718	60S ribosomal protein L1	Rpl18a	20.732	176	P62718	24	RL18A_RA
P49816	Tuberin	Tsc2	201.28	1809	P49816	937	TSC2_RAT
Q7TQ84	UAP56-interacting factor	Fyttd1	35.642	317	Q7TQ84	23	UIF_RAT U
Q6P773	TATA box-binding protei	Taf1c	92.37	842	Q6P773	146	TAF1C_RA
P02688	Myelin basic protein	Mbp	21.502	195	P02688	190	MBP_RAT
P52481	Adenylyl cyclase-associat	Cap2	52.912	477	P52481	309	CAP2_RAT
Q1AAU6	Arf-GAP with SH3 domai	Asap1	127.09	1144	Q1AAU6	1042	ASAP1_RA
Q62599	Metastasis-associated pro	Mta1	79.411	703	Q62599	449	MTA1_RAT
P35280	Ras-related protein Rab-	Rab8a	23.668	207	P35280	185	RAB8A_RA
O08678	Serine/threonine-protein	Mark1	88.234	793	O08678	414	MARK1_RA
P49655	ATP-sensitive inward rect	Kcnj10	42.48	379	P49655	338	KCJ10_RAT
O35413	Sorbin and SH3 domain-	Sorbs2	134.06	1196	O35413	938	SRBS2_RA
Q5XI74	Endonuclease/exonucleas	Eepd1	62.868	569	Q5XI74	110	EEPD1_RA
Q4V8C3	Echinoderm microtubule-	Eml1	89.801	814	Q4V8C3	146	EMAL1_RA
P62909	40S ribosomal protein S3	Rps3	26.674	243	P62909	221	RS3_RAT 4
Q9Z327	Synaptopodin	Synpo	99.985	931	Q9Z327	537	SYNPO_RA
Q8CGU4	Arf-GAP with GTPase, AN	Agap2	124.44	1186	Q8CGU4	517	AGAP2_RA
O70377	Synaptosomal-associatec	Snap23	23.235	210	O70377	109	SNP23_RA
Q5RJM0	MKI67 FHA domain-inter	Nifk	31.351	271	Q5RJM0	244	MK67I_RA
Q5M7W5	Microtubule-associated p	Map4	110.3	1057	Q5M7W5	799	MAP4_RAT
P97526	Neurofibromin	Nf1	317.08	2820	P97526	2798	NF1_RAT I
Q6PDU1	Serine/arginine-rich splic	Srsf2	25.476	221	Q6PDU1	25	SRSF2_RAT

Q9JIT3	Transducin-like enhancer	Tle3	82.643	764	Q9JIT3	203	TLE3_RAT	
O35431	Amyloid-beta A4 precurs	Apba2	82.849	750	O35431	305	APBA2_RA	
O35431	Amyloid-beta A4 precurs	Apba2	82.849	750	O35431	307	APBA2_RA	
Q99JE6	1-phosphatidylinositol 4,!	Plcb3	139.45	1234	Q99JE6	1105	PLCB3_RA	
Q5BJQ2	Ubiquitin carboxyl-termir	Mindy1	52.728	482	Q5BJQ2	455	MINY1_RA	
Q00729	Histone H2B type 1		14.224	127	Q00715;Q	56;57	H2B1_RAT	
Q63625	PHD and RING finger dor	Phrf1	184.21	1685	Q63625	1205	PHRF1_RA	
B2GV22	Phosphatidylserine synth	Ptdss2	54.722	471	B2GV22	14	PTSS2_RA	
P10888	Cytochrome c oxidase su	Cox4i1	19.514	169	P10888	58	COX41_RA	
P61765	Syntaxin-binding protein	Stxbp1	67.568	594	P61765	594	STXB1_RA	
D4A1F2	[F-actin]-monooxygenas	Mical2	110.09	961	D4A1F2	515	MICA2_RA	
O88664	Serine/threonine-protein	Taok1	115.95	1001	O88664	965	TAOK1_RA	
Q5XIB5	Coiled-coil domain-cont	Ccdc86	38.584	341	Q5XIB5	126	CCD86_RA	
O08662	Phosphatidylinositol 4-ki	Pi4ka	236.92	2096	O08662	259	PI4KA_RA	
D4AEC2	Calmodulin-regulated sp	Camsap2	165.61	1470	D4AEC2	450	CAMP2_R	
P41516	DNA topoisomerase 2-al	Top2a	173.22	1526	P41516	1371	TOP2A_RA	
P15865	Histone H1.4	H1-4	21.987	219	P15865	41	H14_RAT	
P62997	Transformer-2 protein hc	Tra2b	33.665	288	P62997	26	TRA2B_RA	
Q5U2W6	Prostate tumor-overexpr	Ptov1	46.848	416	Q5U2W6	53	PTVO1_RA	
P02688	Myelin basic protein	Mbp	21.502	195	P02688	20	MBP_RAT	
Q6P730	Disabled homolog 2-inte	Dab2ip	110	996	Q6P730	785	DAB2P_RA	
P11960	2-oxoisovalerate dehydr	Bckdha	50.164	441	P11960	333	ODBA_RA	
P62856	40S ribosomal protein S2	Rps26	13.015	115	P62856	24	RS26_RAT	
Q9QYF3	Unconventional myosin-\	Myo5a	211.76	1828	Q9QYF3	602	MYO5A_R	
B2GV24	E3 UFM1-protein ligase 1	Ufl1	89.584	793	B2GV24	458	UFL1_RAT	
P84889	Vang-like protein 2	Vangl2	59.77	521	P84889	520	VANG2_R	
P41516	DNA topoisomerase 2-al	Top2a	173.22	1526	P41516	1370	TOP2A_RA	
Q6AYK1	RNA-binding protein wit	Rnps1	34.238	305	Q6AYK1	155	RNPS1_RA	
Q6AYK1	RNA-binding protein wit	Rnps1	34.238	305	Q6AYK1	157	RNPS1_RA	
P42930	Heat shock protein beta-	Hspb1	22.892	206	P42930	15	HSPB1_RA	
Q64632	Integrin beta-4	Itgb4	200.59	1807	Q64632	1436	ITB4_RAT	
Q63358	Unconventional myosin-I	Myo9b	225.03	1980	Q63358	1220	MYO9B_R	
D3ZXD8	Transmembrane protein	Tmem245	97.281	876	D3ZXD8	327	TM245_RA	
P02688	Myelin basic protein	Mbp	21.502	195	P02688	122	MBP_RAT	
O55035	Peptidyl-prolyl cis-trans i	Ppig	88.378	752	O55035	712	PPIG_RAT	
Q9WUD2	Transient receptor potent	Trpv2	86.705	761	Q9WUD2	82	TRPV2_RA	
Q4V7C9	NF-kappa-B-activating p	Nkap	47.266	415	Q4V7C9	147	NKAP_RA	
Q64632	Integrin beta-4	Itgb4	200.59	1807	Q64632	1425	ITB4_RAT	
P62755	40S ribosomal protein S6	Rps6	28.68	249	P62755	247	RS6_RAT	
Q62896	BET1 homolog	Bet1	13.23	118	Q62896	50	BET1_RAT	
Q68FR9	Elongation factor 1-delta	Eef1d	31.33	281	Q68FR9	129	EF1D_RAT	
Q498D5	Regulator of microtubule	Rmdn2	47.217	412	Q498D5	121	RMD2_RA	
Q62747	Synaptotagmin-7	Syt7	45.482	403	Q62747	58	SYT7_RAT	
Q8VH46	Actin filament-associated	Afap1	80.751	731	Q8VH46	549	AFAP1_RA	
P54645	5'-AMP-activated proteir	Prkaa1	63.973	559	P54645	498	AAPK1_RA	
O08961	Zinc finger protein 423	Znf423	147.23	1311	O08961	1187	ZN423_RA	
Q7TSU1	Brefeldin A-inhibited gua	Arfgef2	201.97	1791	Q7TSU1	356	BIG2_RAT	
Q5M7V8	Thyroid hormone recepto	Thrap3	108.25	951	Q5M7V8	248	TR150_RA	
P47752	Sphingosine 1-phosphat	S1pr2	38.734	352	P47752	332	S1PR2_RA	
Q9Z340	Partitioning defective 3 h	Pard3	149.45	1337	Q9Z340	378	PARD3_RA	
P06687	Sodium/potassium-trans	Atp1a3	111.69	1013	P06687	10	AT1A3_RA	
O88777	Presenilin-2	Psen2	50.051	448	O88777	25	PSN2_RAT	
O70531	Sulfate transporter	Slc26a2	82.027	739	O70531	35	S26A2_RA	
Q8CG07	ATPase WRNIP1	Wrnip1	71.933	660	Q8CG07	153	WRIP1_RA	
Q6LED0	Histone H3.1		15.404	136	Q6LED0;P	81;81	H31_RAT	
Q5M7W5	Microtubule-associated	Map4	110.3	1057	Q5M7W5	786	MAP4_RA	
Q4KM77	Etoposide-induced prote	Ei24	38.892	340	Q4KM77	56	EI24_RAT	
Q9WTR8	PH domain leucine-rich r	Phlpp1	183.36	1696	Q9WTR8	374	PHLP1_RA	

P11167	Solute carrier family 2, fac	Slc2a1	53.962	492	P11167	475	GTR1_RAT
P06686	Sodium/potassium-transp	Atp1a2	112.22	1020	P06686	439	AT1A2_RA
Q5M7V8	Thyroid hormone recepto	Thrap3	108.25	951	Q5M7V8	243	TR150_RA
O35821	Myb-binding protein 1A	Mybbp1a	152.28	1344	O35821	14	MBB1A_R/
P06494	Receptor tyrosine-protein	Erbb2	138.83	1257	P06494	703	ERBB2_RA
P70615	Lamin-B1	Lmnb1	66.606	587	P70615	302	LMNB1_R/
P48679	Prelamin-A/C	Lmna	74.323	665	P48679	429	LMNA_RA
Q63638	Striated muscle-specific s	Speg	354.16	3259	Q63638	1177	SPEG_RAT
P34926	Microtubule-associated p	Map1a	299.53	2774	P34926	1307	MAP1A_R/
P21396	Amine oxidase [flavin-con	Maoa	59.507	526	P21396	383	AOFA_RAT
Q5M7W5	Microtubule-associated p	Map4	110.3	1057	Q5M7W5	618	MAP4_RAT
Q4V882	Epsin-3	Epn3	65.047	608	Q4V882	359	EPN3_RAT
Q4V7E8	Leucine-rich repeat flight	Lrrfip2	49.772	437	Q4V7E8	133	LRRF2_RAT
P34926	Microtubule-associated p	Map1a	299.53	2774	P34926	1763	MAP1A_R/
Q5M7W5	Microtubule-associated p	Map4	110.3	1057	Q5M7W5	506	MAP4_RAT
P34064	Proteasome subunit alpha	Psma5	26.391	241	P34064	56	PSA5_RAT
P50137	Transketolase	Tkt	67.643	623	P50137	287	TKT_RAT T
Q2EJA0	Transcriptional coactivato	Yap1	50.501	469	Q2EJA0	95	YAP1_RAT
Q1AAU6	Arf-GAP with SH3 domain	Asap1	127.09	1144	Q1AAU6	1056	ASAP1_RA
Q05764	Beta-adducin	Add2	80.592	725	Q05764	612	ADDB_RAT
P15146	Microtubule-associated p	Map2	202.41	1861	P15146	1359	MTAP2_R/
Q63425	Periaxin	Prx	146.4	1383	Q63425	131	PRAX_RAT
Q6P9V9	Tubulin alpha-1B chain	Tuba1b	50.151	451	Q6P9V9;P	334;334;33	TBA1B_RA
P16884	Neurofilament heavy poly	Nefh	115.38	1072	P16884	654	NFH_RAT
Q5QD51	A-kinase anchor protein 1	Akap12	181.11	1687	Q5QD51	585	AKA12_RA
Q4G091	MICAL C-terminal-like pr	Micalcl	76.256	687	Q4G091	377	MICLK_RA
Q4G091	MICAL C-terminal-like pr	Micalcl	76.256	687	Q4G091	376	MICLK_RA
Q00566	Methyl-CpG-binding pro	Mecp2	53.047	492	Q00566	421	MECP2_R/
Q66HG9	Mitochondrial antiviral-si	Mavs	53.804	507	Q66HG9	238	MAVS_RAT
Q924C3	Ectonucleotide pyrophosp	Enpp1	102.94	906	Q924C3	25	ENPP1_RA
P34926	Microtubule-associated p	Map1a	299.53	2774	P34926	1784	MAP1A_R/
Q62835	Rab GTPase-binding effe	Rabep2	61.972	554	Q62835	180	RABE2_RA
P15205	Microtubule-associated p	Map1b	269.64	2461	P15205	1138	MAP1B_R/
P19332	Microtubule-associated p	Mapt	78.563	752	P19332	191	TAU_RAT
Q4V8H8	EH domain-containing pr	Ehd2	61.237	543	Q4V8H8	468	EHD2_RAT
Q5XII5	Telomerase Cajal body pr	Wrap53	58.195	532	Q5XII5	476	TCAB1_RA
P34926	Microtubule-associated p	Map1a	299.53	2774	P34926	526	MAP1A_R/
P23348	Anion exchange protein 3	Slc4a3	135.41	1227	P23348	170	B3A3_RAT
P15205	Microtubule-associated p	Map1b	269.64	2461	P15205	824	MAP1B_R/
Q4V893	GPALPP motifs-containin	Gpalpp1	39.174	348	Q4V893	106	GPAM1_R/
P15146	Microtubule-associated p	Map2	202.41	1861	P15146	884	MTAP2_R/
Q9JK11	Reticulon-4	Rtn4	126.39	1163	Q9JK11	766	RTN4_RAT
P26431	Sodium/hydrogen exchar	Slc9a1	91.646	820	P26431	707	SL9A1_RA
Q641Y8	ATP-dependent RNA heli	Ddx1	82.496	740	Q641Y8	475	DDX1_RAT
Q925N3	PEX5-related protein	Pex5l	66.871	602	Q925N3	141	PEX5R_RA
O35346	Focal adhesion kinase 1	Ptk2	119.72	1055	O35346	913	FAK1_RAT
Q5QD51	A-kinase anchor protein 1	Akap12	181.11	1687	Q5QD51	350	AKA12_RA
Q9QYM0	Multidrug resistance-asso	Abcc5	160.85	1436	Q9QYM0	43	MRP5_RAT
B2DD29	Serine/threonine-protein	Brsk1	85.182	778	B2DD29	586	BRSK1_RA
Q5M7W5	Microtubule-associated p	Map4	110.3	1057	Q5M7W5	509	MAP4_RAT
Q91Z79	Liprin-alpha-3	Ppfia3	133.43	1192	Q91Z79	142	LIPA3_RAT
P19945	60S acidic ribosomal prot	Rplp0	34.215	317	P19945	307	RLA0_RAT
Q4V8H8	EH domain-containing pr	Ehd2	61.237	543	Q4V8H8	470	EHD2_RAT
Q9ERE6	Myosin phosphatase Rho	Mprip	117.11	1029	Q9ERE6	981	MPRIP_RA
P70501	RNA-binding protein 10	Rbm10	94.387	852	P70501	660	RBM10_R/
Q3SWT4	Protein IWS1 homolog	Iws1	85.447	764	Q3SWT4	183	IWS1_RAT
P34926	Microtubule-associated p	Map1a	299.53	2774	P34926	1767	MAP1A_R/
Q63488	Sodium-dependent phos	Slc20a2	70.747	656	Q63488	423	S20A2_RA

P12839	Neurofilament medium p	Nefm	95.79	846	P12839	751	NFM_RAT
Q9Z1T4	Connector enhancer of ki	Cnksr2	117.39	1032	Q9Z1T4	906	CNKR2_R/
Q9JHB5	Translin-associated prote	Tsnax	33.005	290	Q9JHB5	33	TSNAX_R/
P16884	Neurofilament heavy poly	Nefh	115.38	1072	P16884	508	NFH_RAT
Q5XIT1	Microtubule-associated p	Mapre3	31.966	281	Q5XIT1	162	MARE3_R/
Q63425	Periaxin	Prx	146.4	1383	Q63425	133	PRAX_RAT
Q9QYM0	Multidrug resistance-asso	Abcc5	160.85	1436	Q9QYM0	41	MRP5_RAT
P26431	Sodium/hydrogen exchar	Slc9a1	91.646	820	P26431	801	SL9A1_RA
P16884	Neurofilament heavy poly	Nefh	115.38	1072	P16884	684	NFH_RAT
P19944	60S acidic ribosomal prot	Rplp1	11.498	114	P19944	104	RLA1_RAT
P70580	Membrane-associated pr	Pgrmc1	21.598	195	P70580	181	PGRC1_R/
P11530	Dystrophin	Dmd	425.82	3677	P11530	3616	DMD_RAT
P16884	Neurofilament heavy poly	Nefh	115.38	1072	P16884	762	NFH_RAT
P16884	Neurofilament heavy poly	Nefh	115.38	1072	P16884	690	NFH_RAT
Q5U2M8	Mediator of DNA damage	Mdc1	136.95	1279	Q5U2M8	168	MDC1_RA
P16884	Neurofilament heavy poly	Nefh	115.38	1072	P16884	666	NFH_RAT
Q62632	Follistatin-related protein	Fstl1	34.622	306	Q62632	163	FSTL1_RAT
P31016	Disks large homolog 4	Dlg4	80.464	724	P31016	295	DLG4_RAT
Q9ESN0	Protein Niban 1	Niban1	103.46	937	Q9ESN0	581	NIBA1_RA
Q8VBU2	Protein NDRG2	Ndrg2	40.779	371	Q8VBU2	330	NDRG2_R/
P0DJJ3	SH3-containing GRB2-lik	Sgip1	88.584	827	P0DJJ3	265	SGIP1_RAT
P16884	Neurofilament heavy poly	Nefh	115.38	1072	P16884	528	NFH_RAT
P16884	Neurofilament heavy poly	Nefh	115.38	1072	P16884	534	NFH_RAT
Q4V882	Epsin-3	Epn3	65.047	608	Q4V882	419	EPN3_RAT
Q4V882	Epsin-3	Epn3	65.047	608	Q4V882	417	EPN3_RAT
Q6P9T8	Tubulin beta-4B chain	Tubb4b	49.8	445	Q6P9T8	55	TBB4B_RA
P34926	Microtubule-associated p	Map1a	299.53	2774	P34926	1742	MAP1A_R/
Q5QD51	A-kinase anchor protein 1	Akap12	181.11	1687	Q5QD51	370	AKA12_RA
P04466	Myosin regulatory light cl	Mylpf	18.969	169	P04466	15	MLRS_RAT
P16884	Neurofilament heavy poly	Nefh	115.38	1072	P16884	768	NFH_RAT
P15205	Microtubule-associated p	Map1b	269.64	2461	P15205	1056	MAP1B_R/
Q6AYH5	Dynactin subunit 2	Dctn2	44.147	402	Q6AYH5	83	DCTN2_R/
P10362	Secretogranin-2	Scg2	71.03	619	P10362	558	SCG2_RAT
P10362	Secretogranin-2	Scg2	71.03	619	P10362	557	SCG2_RAT
P11497	Acetyl-CoA carboxylase 1	Acaca	265.19	2345	P11497	25	ACACA_R/
P15205	Microtubule-associated p	Map1b	269.64	2461	P15205	1070	MAP1B_R/
P16884	Neurofilament heavy poly	Nefh	115.38	1072	P16884	714	NFH_RAT
P0DJJ3	SH3-containing GRB2-lik	Sgip1	88.584	827	P0DJJ3	371	SGIP1_RAT
P16884	Neurofilament heavy poly	Nefh	115.38	1072	P16884	414	NFH_RAT
Q6MG08	ATP-binding cassette sub	Abcf1	95.251	839	Q6MG08	109	ABCF1_RA
Q925Q9	SH3 domain-containing k	Sh3kbp1	78.085	709	Q925Q9	156	SH3K1_RA
P37285	Kinesin light chain 1	Klc1	63.744	560	P37285	524	KLC1_RAT
P16884	Neurofilament heavy poly	Nefh	115.38	1072	P16884	672	NFH_RAT
P19527	Neurofilament light polyp	Nefl	61.335	542	P19527	67	NFL_RAT N
P16884	Neurofilament heavy poly	Nefh	115.38	1072	P16884	630	NFH_RAT
Q2TL32	E3 ubiquitin-protein ligas	Ubr4	573.79	5194	Q2TL32	457	UBR4_RAT
Q63560	Microtubule-associated p	Map6	100.48	952	Q63560	681	MAP6_RAT
Q5QD51	A-kinase anchor protein 1	Akap12	181.11	1687	Q5QD51	274	AKA12_RA
O88588	Phosphofurin acidic clust	Pacs1	104.7	961	O88588	428	PACS1_RA
P15205	Microtubule-associated p	Map1b	269.64	2461	P15205	1332	MAP1B_R/
Q80X08	WASH complex subunit 2	Washc2	145.15	1328	Q80X08	387	WASC2_R/
Q5QD51	A-kinase anchor protein 1	Akap12	181.11	1687	Q5QD51	1354	AKA12_RA
P16884	Neurofilament heavy poly	Nefh	115.38	1072	P16884	702	NFH_RAT
P34926	Microtubule-associated p	Map1a	299.53	2774	P34926	894	MAP1A_R/
P16884	Neurofilament heavy poly	Nefh	115.38	1072	P16884	708	NFH_RAT
P23565	Alpha-internexin	Ina	56.115	505	P23565	474	AINX_RAT
Q5XIR9	Ubiquitin-associated don	Ubac1	45.546	409	Q5XIR9	98	UBAC1_R/
O55164	Multiple PDZ domain pro	Mpdz	218.59	2054	O55164	1270	MPDZ_RA

P16884	Neurofilament heavy poly	Nefh	115.38	1072	P16884	846	NFH_RAT
P16884	Neurofilament heavy poly	Nefh	115.38	1072	P16884	588	NFH_RAT
Q80X08	WASH complex subunit 2	Washc2	145.15	1328	Q80X08	531	WASC2_R
P57097	Tyrosine-protein kinase M	Mertk	109.42	994	P57097	561	MERTK_R
P57097	Tyrosine-protein kinase M	Mertk	109.42	994	P57097	558	MERTK_R
P57097	Tyrosine-protein kinase M	Mertk	109.42	994	P57097	565	MERTK_R
O88658	Kinesin-like protein KIF1E	Kif1b	204.17	1816	O88658	1057	KIF1B_RAT
O70511	Ankyrin-3	Ank3	284.44	2622	O70511	855	ANK3_RAT
Q5FVH4	AKT-interacting protein	Aktip	32.991	292	Q5FVH4	30	AKTIP_RA
P16884	Neurofilament heavy poly	Nefh	115.38	1072	P16884	600	NFH_RAT
P16884	Neurofilament heavy poly	Nefh	115.38	1072	P16884	902	NFH_RAT
Q63425	Periaxin	Prx	146.4	1383	Q63425	7	PRAX_RAT
P37285	Kinesin light chain 1	Klc1	63.744	560	P37285	462	KLC1_RAT
Q7TNK6	tRNA (guanine(10)-N2)-r	Trmt11	53.102	463	Q7TNK6	93	TRM11_R
P97839	Disks large-associated pr	Dlgap4	108.03	992	P97839	973	DLGP4_RA
P16884	Neurofilament heavy poly	Nefh	115.38	1072	P16884	651	NFH_RAT
P34926	Microtubule-associated p	Map1a	299.53	2774	P34926	598	MAP1A_R
P97603	Neogenin (Fragment)	Neo1	150.64	1377	P97603	1163	NEO1_RA
Q6IMY8	Heterogeneous nuclear ri	Hnrnpu	87.731	798	Q6IMY8	58	HNRPU_R
P0DJJ3	SH3-containing GRB2-lik	Sgip1	88.584	827	P0DJJ3	335	SGIP1_RA
P16884	Neurofilament heavy poly	Nefh	115.38	1072	P16884	756	NFH_RAT
Q6JE36	Protein NDRG1	Ndrg1	42.954	394	Q6JE36	362	NDRG1_R
P26284	Pyruvate dehydrogenase	Pdha1	43.226	390	P26284	232	ODPA_RA
P16884	Neurofilament heavy poly	Nefh	115.38	1072	P16884	827	NFH_RAT
Q9JK11	Reticulon-4	Rtn4	126.39	1163	Q9JK11	169	RTN4_RAT
P16884	Neurofilament heavy poly	Nefh	115.38	1072	P16884	678	NFH_RAT
Q6RJR6	Reticulon-3	Rtn3	101.52	940	Q6RJR6	111	RTN3_RAT
P15205	Microtubule-associated p	Map1b	269.64	2461	P15205	1205	MAP1B_R
P16884	Neurofilament heavy poly	Nefh	115.38	1072	P16884	660	NFH_RAT
Q5QD51	A-kinase anchor protein	Akap12	181.11	1687	Q5QD51	614	AKA12_RA
Q9JK11	Reticulon-4	Rtn4	126.39	1163	Q9JK11	107	RTN4_RAT
Q2KJ09	Ubiquitin carboxyl-termir	Usp16	93.761	826	Q2KJ09	433	UBP16_RA
Q2KJ09	Ubiquitin carboxyl-termir	Usp16	93.761	826	Q2KJ09	431	UBP16_RA
Q2KJ09	Ubiquitin carboxyl-termir	Usp16	93.761	826	Q2KJ09	434	UBP16_RA
Q63430	Cold shock domain-conta	Csdc2	16.877	154	Q63430	59	CSDC2_RA
P16884	Neurofilament heavy poly	Nefh	115.38	1072	P16884	516	NFH_RAT
P16884	Neurofilament heavy poly	Nefh	115.38	1072	P16884	594	NFH_RAT
P16884	Neurofilament heavy poly	Nefh	115.38	1072	P16884	852	NFH_RAT
P55260	Annexin A4	Anxa4	35.848	319	P55260	12	ANXA4_R
P16884	Neurofilament heavy poly	Nefh	115.38	1072	P16884	782	NFH_RAT
P16884	Neurofilament heavy poly	Nefh	115.38	1072	P16884	816	NFH_RAT
P29067	Beta-arrestin-2	Arrb2	46.34	410	P29067	164	ARRB2_RA
Q5U318	Astrocytic phosphoprotei	Pea15	15.04	130	Q5U318	116	PEA15_RA
P16884	Neurofilament heavy poly	Nefh	115.38	1072	P16884	808	NFH_RAT
A0A096M	Muscular LMNA-interacti	Mlip	86.185	807	A0A096M	85	MLIP_RAT
P15146	Microtubule-associated p	Map2	202.41	1861	P15146	610	MTAP2_R
P16086	Spectrin alpha chain, non	Sptan1	284.63	2472	P16086	1031	SPTN1_RA
Q5U2M8	Mediator of DNA damage	Mdc1	136.95	1279	Q5U2M8	801	MDC1_RA
P0C1X8	AP2-associated protein k	Aak1	103.76	962	P0C1X8	625	AAK1_RAT
P34926	Microtubule-associated p	Map1a	299.53	2774	P34926	1306	MAP1A_R
P16884	Neurofilament heavy poly	Nefh	115.38	1072	P16884	860	NFH_RAT
P16884	Neurofilament heavy poly	Nefh	115.38	1072	P16884	696	NFH_RAT
P61808	Stannin	Snn	9.501	88	P61808	49	SNN_RAT
P15205	Microtubule-associated p	Map1b	269.64	2461	P15205	1436	MAP1B_R
P97876	Basic leucine zipper trans	Batf3	15.129	133	P97876	85	BATF3_RA
Q9QUL6	Vesicle-fusing ATPase	Nsf	82.652	744	Q9QUL6	739	NSF_RAT
P16884	Neurofilament heavy poly	Nefh	115.38	1072	P16884	546	NFH_RAT
P37285	Kinesin light chain 1	Klc1	63.744	560	P37285	460	KLC1_RAT

P34926	Microtubule-associated p	Map1a	299.53	2774	P34926	1254	MAP1A_R
P15146	Microtubule-associated p	Map2	202.41	1861	P15146	521	MTAP2_R
O55170	Transcription factor SOX-	Sox10	50.039	466	O55170	26	SOX10_RA
P16884	Neurofilament heavy poly	Nefh	115.38	1072	P16884	624	NFH_RAT
P16884	Neurofilament heavy poly	Nefh	115.38	1072	P16884	832	NFH_RAT
P16884	Neurofilament heavy poly	Nefh	115.38	1072	P16884	692	NFH_RAT
B2GV05	RNA-binding protein 5	Rbm5	92.351	815	B2GV05	624	RBM5_RAT
Q9JI66	Electrogenic sodium bica	Slc4a4	121.34	1079	Q9JI66	245	S4A4_RAT
P16884	Neurofilament heavy poly	Nefh	115.38	1072	P16884	540	NFH_RAT
P15205	Microtubule-associated p	Map1b	269.64	2461	P15205	609	MAP1B_R
Q9EPJ0	Nuclear ubiquitous casein	Nucks1	27.14	243	Q9EPJ0	223	NUCKS_R
P31000	Vimentin	Vim	53.732	466	P31000	459	VIME_RAT
P31000	Vimentin	Vim	53.732	466	P31000	458	VIME_RAT
P34926	Microtubule-associated p	Map1a	299.53	2774	P34926	872	MAP1A_R
P13668	Stathmin	Stmn1	17.288	149	P13668	25	STMN1_R
D3ZML2	Serine/threonine-protein	Brsk2	81.454	735	D3ZML2	394	BRSK2_RA
P16884	Neurofilament heavy poly	Nefh	115.38	1072	P16884	669	NFH_RAT
Q5QD51	A-kinase anchor protein	Akap12	181.11	1687	Q5QD51	685	AKA12_RA
P02401	60S acidic ribosomal prot	Rplp2	11.692	115	P02401	102	RLA2_RAT
Q4V8B0	Oxidation resistance prot	Oxr1	92.809	839	Q4V8B0	197	OXR1_RAT
P16884	Neurofilament heavy poly	Nefh	115.38	1072	P16884	618	NFH_RAT
Q9JKC9	Synergin gamma	Synrg	141.36	1329	Q9JKC9	565	SYNRG_R
P34926	Microtubule-associated p	Map1a	299.53	2774	P34926	1897	MAP1A_R
Q9WU70	Syntaxin-binding protein	Stxbp5	127.66	1152	Q9WU70	693	STXB5_RA
P04466	Myosin regulatory light cl	Mylpf	18.969	169	P04466	16	MLRS_RAT
P19527	Neurofilament light polyp	Nefl	61.335	542	P19527	473	NFL_RAT N
P13383	Nucleolin	Ncl	77.146	713	P13383	566	NUCL_RAT
Q9ESM0	Inositol hexakisphosphate	Ip6k1	49.365	433	Q9ESM0	139	IP6K1_RAT
Q9WTQ1	Serine/threonine-protein	Prkd1	102.04	918	Q9WTQ1	203	KPCD1_RA
Q5M7W5	Microtubule-associated p	Map4	110.3	1057	Q5M7W5	546	MAP4_RAT
Q64548	Reticulon-1	Rtn1	83.001	777	Q64548	348	RTN1_RAT
P35281	Ras-related protein Rab-	Rab10	22.858	200	P35281	73	RAB10_RA
P12839	Neurofilament medium p	Nefm	95.79	846	P12839	545	NFM_RAT
P15205	Microtubule-associated p	Map1b	269.64	2461	P15205	2065	MAP1B_R
P08721	Osteopontin	Spp1	34.963	317	P08721	270	OSTP_RAT
Q5PQM2	Kinesin light chain 4	Klc4	68.963	619	Q5PQM2	590	KLC4_RAT
P16884	Neurofilament heavy poly	Nefh	115.38	1072	P16884	417	NFH_RAT
Q9ESB5	N-terminal EF-hand calci	Necab1	40.903	352	Q9ESB5	15	NECA1_R
Q5M7W5	Microtubule-associated p	Map4	110.3	1057	Q5M7W5	520	MAP4_RAT
P12839	Neurofilament medium p	Nefm	95.79	846	P12839	507	NFM_RAT
P34926	Microtubule-associated p	Map1a	299.53	2774	P34926	1574	MAP1A_R
Q63544	Gamma-synuclein	Sncg	12.976	123	Q63544	120	SYUG_RAT
Q5M7W5	Microtubule-associated p	Map4	110.3	1057	Q5M7W5	978	MAP4_RAT
B5DFC8	Eukaryotic translation init	Eif3c	105.43	911	B5DFC8	45	EIF3C_RAT
Q64548	Reticulon-1	Rtn1	83.001	777	Q64548	210	RTN1_RAT
P15205	Microtubule-associated p	Map1b	269.64	2461	P15205	1788	MAP1B_R
P02091	Hemoglobin subunit beta	Hbb	15.979	147	P02091;P1	140;140	HBB1_RAT
Q6AXU6	Jupiter microtubule assoc	JPT1	15.575	149	Q6AXU6	74	JUPI1_RAT
P16884	Neurofilament heavy poly	Nefh	115.38	1072	P16884	732	NFH_RAT
P16884	Neurofilament heavy poly	Nefh	115.38	1072	P16884	606	NFH_RAT
P15205	Microtubule-associated p	Map1b	269.64	2461	P15205	1781	MAP1B_R
P15205	Microtubule-associated p	Map1b	269.64	2461	P15205	884	MAP1B_R
P37285	Kinesin light chain 1	Klc1	63.744	560	P37285	521	KLC1_RAT
P15205	Microtubule-associated p	Map1b	269.64	2461	P15205	1317	MAP1B_R
P16884	Neurofilament heavy poly	Nefh	115.38	1072	P16884	750	NFH_RAT
Q56B11	Proline-, glutamic acid- a	Pelp1	119.14	1130	Q56B11	757	PELP1_RAT
Q80X08	WASH complex subunit 2	Washc2	145.15	1328	Q80X08	720	WASC2_R
P02091	Hemoglobin subunit beta	Hbb	15.979	147	P02091	51	HBB1_RAT

P15205	Microtubule-associated p	Map1b	269.64	2461	P15205	2027	MAP1B_RA
P34926	Microtubule-associated p	Map1a	299.53	2774	P34926	1134	MAP1A_RA
Q66HA8	Heat shock protein 105 kl	Hsph1	96.417	858	Q66HA8	810	HS105_RA
Q9QYU1	Peroxisomal biogenesis fa	Pex19	32.497	299	Q9QYU1	54	PEX19_RA
P15146	Microtubule-associated p	Map2	202.41	1861	P15146	1051	MTAP2_RA
P35465	Serine/threonine-protein	Pak1	60.577	544	P35465	222	PAK1_RAT
Q4KLH6	Centrosomal protein of 1	Cep162	161	1403	Q4KLH6	487	CE162_RA
P15205	Microtubule-associated p	Map1b	269.64	2461	P15205	1618	MAP1B_RA
Q9ERE6	Myosin phosphatase Rho	Mprip	117.11	1029	Q9ERE6	1020	MPRIP_RA
Q9Z340	Partitioning defective 3 h	Pard3	149.45	1337	Q9Z340	668	PARD3_RA
Q62785	28 kDa heat- and acid-st	Pdap1	20.605	181	Q62785	60	HAP28_RA
P16884	Neurofilament heavy poly	Nefh	115.38	1072	P16884	582	NFH_RAT
P08733	Myosin regulatory light cl	Myl2	18.88	166	P08733	15	MLRV_RAT
P15205	Microtubule-associated p	Map1b	269.64	2461	P15205	1315	MAP1B_RA
Q62785	28 kDa heat- and acid-st	Pdap1	20.605	181	Q62785	63	HAP28_RA
P16884	Neurofilament heavy poly	Nefh	115.38	1072	P16884	591	NFH_RAT
P16884	Neurofilament heavy poly	Nefh	115.38	1072	P16884	612	NFH_RAT
O35430	Amyloid-beta A4 precurs	Apba1	92.653	839	O35430	264	APBA1_RA
P15205	Microtubule-associated p	Map1b	269.64	2461	P15205	1009	MAP1B_RA
P15146	Microtubule-associated p	Map2	202.41	1861	P15146	939	MTAP2_RA
P15205	Microtubule-associated p	Map1b	269.64	2461	P15205	1321	MAP1B_RA
P15205	Microtubule-associated p	Map1b	269.64	2461	P15205	1874	MAP1B_RA
P15205	Microtubule-associated p	Map1b	269.64	2461	P15205	1257	MAP1B_RA
P16884	Neurofilament heavy poly	Nefh	115.38	1072	P16884	687	NFH_RAT
P27321	Calpastatin	Cast	77.312	713	P27321	175	ICAL_RAT
P15205	Microtubule-associated p	Map1b	269.64	2461	P15205	1626	MAP1B_RA
P62521	Coiled-coil domain-conta	Ccdc8	69.588	643	P62521	200	CCDC8_RA
P12839	Neurofilament medium p	Nefm	95.79	846	P12839	793	NFM_RAT
B5DF41	Syntaphilin	Snph	54.493	504	B5DF41	219	SNPH_RAT
P34926	Microtubule-associated p	Map1a	299.53	2774	P34926	1022	MAP1A_RA
P12839	Neurofilament medium p	Nefm	95.79	846	P12839	721	NFM_RAT
P12839	Neurofilament medium p	Nefm	95.79	846	P12839	761	NFM_RAT
Q5XIL2	Phosphatidylinositol 4-ki	Pi4k2b	54.464	477	Q5XIL2	189	P4K2B_RA
Q5XIL2	Phosphatidylinositol 4-ki	Pi4k2b	54.464	477	Q5XIL2	182	P4K2B_RA
P16884	Neurofilament heavy poly	Nefh	115.38	1072	P16884	880	NFH_RAT
Q68FR9	Elongation factor 1-delta	Eef1d	31.33	281	Q68FR9	119	EF1D_RAT
P34926	Microtubule-associated p	Map1a	299.53	2774	P34926	1148	MAP1A_RA
P15205	Microtubule-associated p	Map1b	269.64	2461	P15205	1905	MAP1B_RA
P16884	Neurofilament heavy poly	Nefh	115.38	1072	P16884	744	NFH_RAT
P15146	Microtubule-associated p	Map2	202.41	1861	P15146	1024	MTAP2_RA
P16884	Neurofilament heavy poly	Nefh	115.38	1072	P16884	720	NFH_RAT
Q66H20	Polypyrimidine tract-binc	Ptbp2	57.488	531	Q66H20	308	PTBP2_RA
O35923	Breast cancer type 2 susc	Brca2	372.21	3343	O35923	3322	BRCA2_RA
P34926	Microtubule-associated p	Map1a	299.53	2774	P34926	1643	MAP1A_RA
P15205	Microtubule-associated p	Map1b	269.64	2461	P15205	1148	MAP1B_RA
P02401	60S acidic ribosomal prot	Rplp2	11.692	115	P02401	79	RLA2_RAT
P15205	Microtubule-associated p	Map1b	269.64	2461	P15205	1496	MAP1B_RA
P15205	Microtubule-associated p	Map1b	269.64	2461	P15205	1393	MAP1B_RA
P19332	Microtubule-associated p	Mapt	78.563	752	P19332	711	TAU_RAT
Q5EB94	Myocardial zonula adhere	Myzap	53.955	466	Q5EB94	247	MYZAP_RA
Q5EB94	Myocardial zonula adhere	Myzap	53.955	466	Q5EB94	249	MYZAP_RA
Q5EB94	Myocardial zonula adhere	Myzap	53.955	466	Q5EB94	246	MYZAP_RA
O35314	Secretogranin-1	Chgb	77.535	675	O35314	155	SCG1_RAT
P19332	Microtubule-associated p	Mapt	78.563	752	P19332	714	TAU_RAT
P34926	Microtubule-associated p	Map1a	299.53	2774	P34926	1460	MAP1A_RA
P15205	Microtubule-associated p	Map1b	269.64	2461	P15205	1382	MAP1B_RA
Q8CGZ2	Afadin- and alpha-actinir	Ssx2ip	70.678	613	Q8CGZ2	313	ADIP_RAT
P15205	Microtubule-associated p	Map1b	269.64	2461	P15205	1765	MAP1B_RA

P15205	Microtubule-associated p	Map1b	269.64	2461	P15205	1494	MAP1B_RA
Q64548	Reticulon-1	Rtn1	83.001	777	Q64548	350	RTN1_RAT
P70483	Striatin	Strn	86.225	780	P70483	245	STRN_RAT
P15205	Microtubule-associated p	Map1b	269.64	2461	P15205	1371	MAP1B_RA
P34980	Prostaglandin E2 recepto	Ptger3	39.942	365	P34980	293	PE2R3_RA
Q6P7P5	Basic leucine zipper and \	Bzw1	48.043	419	Q6P7P5	413	BZW1_RAT
P35465	Serine/threonine-protein	Pak1	60.577	544	P35465	224	PAK1_RAT
P15205	Microtubule-associated p	Map1b	269.64	2461	P15205	1150	MAP1B_RA
P15205	Microtubule-associated p	Map1b	269.64	2461	P15205	1925	MAP1B_RA
Q66H76	Paxillin	Pxn	64.018	586	Q66H76	132	PAXI_RAT
Q5QD51	A-kinase anchor protein 1	Akap12	181.11	1687	Q5QD51	1352	AKA12_RA
P12839	Neurofilament medium p	Nefm	95.79	846	P12839	609	NFM_RAT
P15205	Microtubule-associated p	Map1b	269.64	2461	P15205	1908	MAP1B_RA
P15205	Microtubule-associated p	Map1b	269.64	2461	P15205	1248	MAP1B_RA
P15205	Microtubule-associated p	Map1b	269.64	2461	P15205	1274	MAP1B_RA
Q5U1Z0	Rab3 GTPase-activating p	Rab3gap2	154.43	1386	Q5U1Z0	976	RBGPR_RA
Q5QD51	A-kinase anchor protein 1	Akap12	181.11	1687	Q5QD51	1571	AKA12_RA
P15205	Microtubule-associated p	Map1b	269.64	2461	P15205	1624	MAP1B_RA
P12839	Neurofilament medium p	Nefm	95.79	846	P12839	713	NFM_RAT
P12839	Neurofilament medium p	Nefm	95.79	846	P12839	604	NFM_RAT
P15205	Microtubule-associated p	Map1b	269.64	2461	P15205	1790	MAP1B_RA
O35314	Secretogranin-1	Chgb	77.535	675	O35314	100	SCG1_RAT
P70580	Membrane-associated pr	Pgrmc1	21.598	195	P70580	178	PGRC1_RA
Q5XIS7	Ubiquitin-associated prot	Ubap1	55.139	502	Q5XIS7	146	UBAP1_RA
Q9JIR0	Peripheral-type benzodia	Tspoap1	200.2	1847	Q9JIR0	149	RIMB1_RA
Q9JIR0	Peripheral-type benzodia	Tspoap1	200.2	1847	Q9JIR0	148	RIMB1_RA
Q9JIR0	Peripheral-type benzodia	Tspoap1	200.2	1847	Q9JIR0	153	RIMB1_RA
P12839	Neurofilament medium p	Nefm	95.79	846	P12839	767	NFM_RAT
P12839	Neurofilament medium p	Nefm	95.79	846	P12839	551	NFM_RAT
P12839	Neurofilament medium p	Nefm	95.79	846	P12839	550	NFM_RAT
P15205	Microtubule-associated p	Map1b	269.64	2461	P15205	1942	MAP1B_RA
P15205	Microtubule-associated p	Map1b	269.64	2461	P15205	1789	MAP1B_RA
Q80X08	WASH complex subunit 2	Washc2	145.15	1328	Q80X08	744	WASC2_RA
P15205	Microtubule-associated p	Map1b	269.64	2461	P15205	1786	MAP1B_RA
P15205	Microtubule-associated p	Map1b	269.64	2461	P15205	1380	MAP1B_RA
Q9JJ19	Na(+)/H(+) exchange reg	Slc9a3r1	38.83	356	Q9JJ19	275	NHRF1_RA
Q6P3V7	Tetratricopeptide repeat p	Ttc41	150.28	1309	Q6P3V7	1180	TTC41_RA
Q6P3V7	Tetratricopeptide repeat p	Ttc41	150.28	1309	Q6P3V7	1178	TTC41_RA
P34926	Microtubule-associated p	Map1a	299.53	2774	P34926	980	MAP1A_RA
Q9JK11	Reticulon-4	Rtn4	126.39	1163	Q9JK11	295	RTN4_RAT
B5DF21	Protein Smaug homolog	Samd4a	67.15	610	B5DF21	472	SMAG1_RA
Q5M876	N-acyl-aromatic-L-amin	Acy3	35.419	319	Q5M876	193	ACY3_RAT
P15205	Microtubule-associated p	Map1b	269.64	2461	P15205	1201	MAP1B_RA
P34926	Microtubule-associated p	Map1a	299.53	2774	P34926	1375	MAP1A_RA
P34926	Microtubule-associated p	Map1a	299.53	2774	P34926	1160	MAP1A_RA
P34926	Microtubule-associated p	Map1a	299.53	2774	P34926	1447	MAP1A_RA
P34926	Microtubule-associated p	Map1a	299.53	2774	P34926	984	MAP1A_RA
P12839	Neurofilament medium p	Nefm	95.79	846	P12839	667	NFM_RAT
P15205	Microtubule-associated p	Map1b	269.64	2461	P15205	1778	MAP1B_RA
P15205	Microtubule-associated p	Map1b	269.64	2461	P15205	1772	MAP1B_RA
P12839	Neurofilament medium p	Nefm	95.79	846	P12839	643	NFM_RAT
Q9JK11	Reticulon-4	Rtn4	126.39	1163	Q9JK11	487	RTN4_RAT
P15146	Microtubule-associated p	Map2	202.41	1861	P15146	449	MTAP2_RA
Q5BK82	E3 ubiquitin-protein ligas	Trim69	57.214	499	Q5BK82	91	TRI69_RAT
Q5BK82	E3 ubiquitin-protein ligas	Trim69	57.214	499	Q5BK82	87	TRI69_RAT
P16884	Neurofilament heavy poly	Nefh	115.38	1072	P16884	436	NFH_RAT

Leading.pı	Positions.v	Position	Amino.aci	Sequence.	Score	Score.for.l	Phospho..:	Phospho..:
Q9JKS6	2685	2685	T	DAIDLRTIF	11.65	11.65	S(1)EVKVT	S(11.65)EV
B5DF41	200	200	S	EVAQNGV	37.613	37.613	EEGT(0.00	EEGT(-23.
Q9JI66	1069	1069	S	QQPFLSDI	60.892	60.892	S(0.697)S(	S(4.51)S(-
P09760	45	45	T	KFMALRIK	17.402	17.402	EY(1)AY(1)	EY(17.4)AY
P09760	44	44	Y	KKFMALRI	17.402	17.402	EY(1)AY(1)	EY(17.4)AY
P09760	42	42	Y	TVKKFMA	17.402	17.402	EY(1)AY(1)	EY(17.4)AY
Q9JI66	223	223	S	LLRKHRH(	75.378	75.378	S(1)LADIG	S(75.38)LA
Q5XFX0	163	163	S	PNWFPKK	83.087	46.158	NFS(1)DN	NFS(46.16
O88923	1458	1458	S	AGRQLQN	61.641	61.641	GNS(0.997	GNS(24.79
Q66HA4	494	494	T	KRMGSQC	18.731	18.731	VNDAS(1)	VNDAS(18
Q0V8T4	921	921	T	GHFQLQL	15.422	15.422	GHFQLQL	GHFQLQL
Q4L0E8	220	220	T	RPQRPTN	16.254	16.254	RS(1)RS(1)	RS(16.25)F
Q4L0E8	221	221	Y	PQRPTNLI	16.254	16.254	RS(1)RS(1)	RS(16.25)F
Q08013	105	105	S	QKREDAV	71.555	32.498	KLS(1)EAD	KLS(32.5)E
P11884	8	8	T	________MI	9.7809	9.7809	MLRAALS(	MLRAALS(
Q5BK81	350	350	S	GGNIGKQ	86.472	86.472	ISEDS(0.01	IS(-46.83)I
Q3B8Q0	231	231	S	TPSRPSSA	92.239	92.239	ASS(0.021	AS(-40.07
Q6AY91	116	116	Y	LLFNYKPL	22.435	22.435	PLDT(0.93	PLDT(7.97
Q6AY91	110	110	T	LIIEGFLLFI	22.435	22.435	PLDT(0.93	PLDT(7.97
Q6AY91	119	119	T	NYKPLDTI	22.435	22.435	PLDT(0.93	PLDT(7.97
Q6AY91	115	115	S	FLLFNYKP	22.435	22.435	PLDT(0.93	PLDT(7.97
P62859	23	23	S	PIKLARVTI	73.877	73.877	T(0.171)GS	T(-6.86)GS
G3V928	4521	4521	S	PVYATLYN	43.68	43.68	HS(0.987)I	HS(19.43)I
Q62901	655	655	S	ASPLKNTK	49.632	49.632	VAS(0.873	VAS(9.02)I
P60203	118	118	T	DYKTTICG	79.283	79.283	GLS(0.008	GLS(-20.5
P59649	60	60	S	ISKKVKCR	82.279	82.279	S(0.002)ES	S(-26.91)E
Q4V888	89	89	T	HVVKCTV(	58.093	49.463	CTVCNEA	CT(-39.71
P68403	500	500	T	FGMCKEN	157.7	157.7	T(0.997)FC	T(25.22)FC
O09032	42	42	T	NGPSSNN	59.57	59.57	NCPS(0.21	NCPS(-5.6
Q3ZB98	351	351	S	TAKSSKGS	56.684	56.684	GSSQPGQ	GS(-34.84
Q4V8J4	65	65	S	TLRIEKGAI	57.174	57.174	S(0.583)PA	S(1.46)PAS
A0A0G2K2	212	212	S	EYGLRLAS	91.123	91.123	EIS(0.91)Q	EIS(10.03)(
P30009	26	26	S	KGEAAAEI	92.95	92.95	GEAAAERI	GEAAAERI
D3ZGB1	1020	1020	T	ETGAQAK	11.385	11.385	QIQS(0.70	QIQS(0)S(
D3ZGB1	1040	1040	T	QHSGDSQ	11.385	11.385	QIQS(0.70	QIQS(0)S(
P29995	1855	1855	S	DSDLMAL	52.716	52.716	DS(0.578)S	DS(1.37)S(
P63059	273	273	T	IMSLRAAV	17.078	14.258	Y(0.685)DI	Y(5.95)DPI
P63059	275	275	T	SLRAAVRY	17.078	17.078	Y(0.841)DI	Y(7.42)DPI
P63059	267	267	Y	KGCCMEII	17.078	17.078	Y(0.841)DI	Y(7.42)DPI
Q8VGC3	501	501	T	PTLASNSC	14.015	14.015	T(1)DRS(1	T(14.02)DI
F1LP64	238	238	S	KQGKDQI	70.919	70.919	S(0.062)AS	S(-10.33)A
P60669	164	164	S	GKASRAE(	46.964	46.964	AEGEDCG	AEGEDCG
P06907	206	206	S	QRRLSAM	84.213	50.353	S(0.164)S(	S(-7.05)S(
Q5RKH1	427	427	S	PRTRPRDI	55.401	55.401	S(0.694)KI	S(3.56)KD
O09032	38	38	S	SNTSNGPS	93.776	89.917	NCPS(0.99	NCPS(27.5
Q75PQ8	3	3	T	__________	24.792	24.792	MS(1)T(1)I	MS(24.79)
Q62737	147	147	T	TPIEPKPKE	39.163	39.163	ERPQVGG	ERPQVGG
P42930	90	90	S	PAFSRALN	136.81	136.81	QLS(0.223	QLS(-3.94
Q5XII9	38	38	S	RRIGTNLP	106.93	106.93	AS(0.991)F	AS(20.47)F
O35828	459	459	S	SLSSTSGI(	113.65	109.84	S(0.996)LQ	S(25.22)LQ
Q5M7V8	238	238	S	WPDATTY	54.094	54.094	AS(0.813)\	AS(7.28)VS
Q9EPA0	104	104	S	KSHNLRAI	66.498	66.498	LEAFS(0.0(	LEAFS(-23
Q9JKU0	214	214	Y	LLTVSLVQ	15.756	15.756	HY(0.793)S	HY(5.15)S(
P84903	257	257	S	KKMMKDI	41.378	41.378	AEQS(1)LH	AEQS(41.3
Q03555	327	327	S	VASRVGSI	43.897	43.897	CS(0.5)S(0	CS(0)S(0)K
Q03555	326	326	S	GVASRVG	43.897	43.897	CS(0.5)S(0	CS(0)S(0)K
O35430	570	570	S	NIVVLMAI	86.856	86.856	S(0.133)NS	S(-8.02)NS

Q99P82	197	197	S	AQSFGEN	145.1	145.1	FYYS(0.00	FY(-121.62
Q9R1N3	238	238	S	HQNEKRF	66.27	66.27	S(1)FADIG	S(66.27)FA
Q499S9	49	49	S	EPSFLQPL	98.737	98.737	S(0.818)VS	S(6.99)VS(
O08875	45	45	S	SPGSLRKQ	78.088	78.088	ISQHGGS(	IS(-34.76)
Q4G008	270	270	Y	AEMAVSR	91.238	91.238	VSTGDT(0	VS(-38.99)
O35052	18	18	T	ELRHRGG	52.555	52.555	GGCPGPG	GGCPGPG
Q9Z1Z1	547	547	S	VRRLFHPC	29.733	29.733	KES(0.753)	KES(6.93)E
Q6AYA6	168	168	S	IAKLITSFLI	100.02	76.064	LES(0.99)P	LES(19.81)
P63149	3	3	T	_________	6.3643	6.3643	S(1)T(1)PA	S(6.36)T(6.
Q9QZ86	507	507	S	KKKKDKKK	120.13	120.13	HIKEEPLS(	HIKEEPLS(
Q9JHY1	285	285	S	KKGTAPGI	76.228	76.228	VIYSQPS(1	VIY(-59.93
O35814	481	481	S	DGYQRCN	59.908	59.908	HDS(1)PEI	HDS(59.91
O35814	476	476	Y	CKEAADG	16.939	16.939	CMMAQY	CMMAQY
Q5FVJ6	280	280	S	RHRFSLQV	85.924	85.924	DNS(1)GH	DNS(85.92
Q63135	554	554	S	LTSQENNS	87.308	78.939	NS(0.987)I	NS(18.92)I
Q5RKH0	166	166	S	SADRGSK	88.37	88.37	AQEQS(1)	AQEQS(88
P06907	205	205	S	LQRRLSAN	66.299	64.297	S(0.989)S(	S(19.99)S(
Q9QZM5	224	224	T	MTSPARL	38.238	38.238	T(0.5)AS(0	T(0)AS(0)L
P52796	282	282	S	RHRKHTQ	96.464	96.464	AAALS(0.0	AAALS(-1
Q63269	2669	2669	S	KRRQRLGI	160.18	160.18	LGFVDVQ	LGFVDVQ
P61314	34	34	S	MRFLLRVF	61.78	61.78	QLS(1)ALH	QLS(61.78
Q9JLH5	1896	1896	S	RGNLELRF	39.531	39.531	AAHPGT(0	AAHPGT(-
Q9R1N3	1180	1180	S	LQGDGDT	63.408	63.408	GS(1)LLQI	GS(63.41)L
P08050	306	306	S	GDRNNSS	77.871	77.871	QAS(1)EQ	QAS(57.48
P34900	187	187	S	RKKDEGSY	106.67	106.67	KPS(0.96)S	KPS(13.8)S
Q5M7V8	532	532	S	NFRVTAYI	113.5	113.5	SS(0.004)S	S(-40.71)S
Q99P82	196	196	S	DAQSFGE	132.31	132.31	FYYSS(0.0	FY(-101.76
Q9Z327	134	134	S	PVPRVAQ	63.816	63.816	S(0.006)T(	S(-21.94)T
Q9JK71	954	954	S	EEHHGPPS	55.353	55.353	QS(1)PALC	QS(55.35)I
Q3ZB98	547	547	T	QQPTVEV	48.305	48.305	EPAPCVQ	EPAPCVQ
Q5FVI4	9	9	S	______ME	85.958	14.561	MES(0.393	MES(-6.65
Q9EPH2	41	41	T	ANGQENC	89.171	89.171	S(0.001)N	S(-32.26)N
Q923J6	2307	2307	T	WREDIKPI	16.306	16.306	PNLMS(1)	PNLMS(16
Q923J6	2305	2305	Y	NEWREDII	16.306	16.306	PNLMS(1)	PNLMS(16
Q62901	612	612	S	RASPVNEI	48.555	48.555	NS(0.711)I	NS(4.02)PS
P52796	280	280	S	RKRHRKHT	84.753	69.864	AAALS(0.9	AAALS(12.
P16443	164	164	S	APSPGPGS	40.496	40.496	S(0.846)S(	S(7.41)S(-
Q5FVI4	15	15	T	_MESRGKS	53.751	53.751	S(0.013)AS	S(-16.2)AS
P06765	34	34	S	LGLLLLPAY	47.204	47.204	AS(0.993)I	AS(22.31)I
P31596	520	520	S	SQHRMHI	85.845	85.845	T(0.004)Q	T(-24.41)Q
P18265;P1	279;216	216	Y	FGSAKQLY	149.96	149.96	GEPNVS(0	GEPNVS(-
P29975	247	247	S	APRSSDFT	158.8	158.8	VWT(0.142	VWT(-7.8)
Q8K4S7	527	527	T	DLIQKGIV	32.329	32.329	S(0.004)PC	S(-22.53)P
Q9WVC0	425	425	T	WEAQQRI	52.522	52.522	ILEQQNS(	ILEQQNS(
Q9Z2Q7	102	102	S	VTRERLLL	82.85	62.924	NEGS(1)EI	NEGS(62.9
A6YP92	22	22	T	EEGCSERP	14.31	14.31	S(0.687)PT	S(0.15)PT(
P60203	114	114	S	QIFGDYKT	113.22	113.22	GLS(0.997	GLS(24.63
Q9JI66	257	257	S	DNGSPAN	104.84	104.84	NLT(0.021	NLT(-15.1
P41123	139	139	S	LILFPRKPS	48.659	43.991	KGDS(0.87	KGDS(8.25
P52591	421	421	T	PTSSPFSSI	42.976	42.976	S(0.218)Q	S(-5.55)Q
Q498U0	26	26	S	DGVRERR	44.382	44.382	S(1)QDEP	S(34.04)QI
P06907	195	195	S	RYCWLRR	93.667	52.49	RLS(1)AMI	RLS(52.49)
Q5EXX3	130	130	S	GISFLEDLS	80.236	80.236	NFS(0.055	NFS(-12.3
Q810W7	1268	1268	S	KSAEPPRS	49.715	49.715	RVQS(1)AI	RVQS(49.7
Q0KL00	1633	1633	S	SQELLANA	146.17	146.17	T(0.311)AS	T(-3.45)AS
Q5EXX3	939	939	S	RKIRKVKW	53.597	53.597	GS(0.019)I	GS(-17.04
Q64649	879	879	T	PREKTISAI	24.041	24.041	T(0.637)IS(	T(-0.48)IS(
Q64649	875	875	Y	LPPEPREK	24.041	24.041	T(0.637)IS(	T(-0.48)IS(

Q64649	870	870	S	HLTVGLPF	24.041	24.041	T(0.637)IS(	T(-0.48)IS(
Q63796	569	569	S	ALSGVGLF	65.246	65.246	APPS(1)PC	APPS(65.2
P15146	1541	1541	S	AFKQAKD	78.69	78.69	VTDGIT(0.	VT(-32.83)
P59649	56	56	S	IIIIISKKVK(	35.711	35.711	KADS(0.83	KADS(6.09
P59649	58	58	S	IIISKKVKCI	35.711	35.711	KADS(0.83	KADS(6.09
P15146	1539	1539	T	PSAFKQAI	54.598	44.294	VT(0.006)[	VT(-21.82)
Q2THW7	380	380	S	AAMPHSS	57.414	57.414	GDS(0.998	GDS(29.45
P06907	229	229	T	RQTPVLY/	62.303	62.303	S(0.101)T(	S(-9.51)T(
O08678;O	215;208;2:	215	T	DFGFSNEF	87.319	48.968	LDT(0.983	LDT(17.9)F
Q6AYJ1	602	602	S	TMQVKRS	62.68	59.709	AAS(1)PE/	AAS(51.79
Q02294	921	921	S	TQVRCERS	38.3	38.3	RGS(0.998	RGS(27.66
Q63488	259	259	S	GRLEKESA	79.059	79.059	ASDES(1)L	AS(-53.48)
Q62649	56	56	S	PPGTQPIA	55.261	55.261	SEVFRY(0.	S(-38.49)E
P00564	372	372	S	VKLMVEM	41.401	41.401	GQS(1)IDI	GQS(41.4)
Q5JCS6	1353	1353	S	EVSSHSSG	48.948	48.948	S(0.163)GS	S(-6.14)GS
Q5BJP5	24	24	S	ATGIPSSK'	157.23	112.95	LS(0.028)S	LS(-14.61)
O35413	1113	1113	S	KGSEDYPI	106.03	106.03	GSEDYPDI	GS(-90.08
D3ZBN0;P	103;104	104	S	SKGTLVQ'	75.566	75.566	GTGAS(0.(	GT(-50.13
Q9WVC0	333	333	S	YNGVDNN	92.098	92.098	GQLT(0.03	GQLT(-14
P26684	424	424	S	NQEQNHI	58.37	58.37	SSHKDS(1	S(-39.62)S
Q6P767	171	171	Y	DEIRKKYG	18.161	18.161	EQNPY(1)I	EQNPY(18
P70615	576	576	T	PIGVPLEEI	68.626	63.216	FHQQGT(:	FHQQGT((
Q9R066	323	323	S	SNMEGYS	33.886	33.886	T(0.002)Q'	T(-27.65)C
P06907	228	228	S	GRQTPVL'	48.741	48.741	S(0.5)T(0.5	S(0)T(0)KA
Q6AXY7	525	525	S	DSKDQSD	95.417	95.417	S(0.003)QS	S(-25.33)C
Q62671	970	970	S	ISVVSSNG	110.12	110.12	AGS(0.121	AGS(-8.51
Q6URK4	359	359	S	YGPMKGC	116.39	116.39	S(0.001)S(	S(-30.65)S
P60203	116	116	T	FGDYKTTI	90.614	60.161	GLS(0.162	GLS(-7.13)
Q63312	14	14	S	__LTLGAR(	128.21	128.21	S(0.019)PS	S(-16.9)PS
Q62636	39	39	S	VQGIFVEK	100.22	100.22	YDPTIEDS	Y(-77.31)D
D3ZFB6	242	242	S	VEEDRIGR	42.629	42.629	AHGGHPC	AHGGHPC
Q8R4S8	278	278	S	RVKEALLP	63.401	63.401	GS(1)FPGL	GS(63.4)FF
Q5RKH0	130	130	S	EERSRPNS	64.439	45.433	KLS(0.988)	KLS(19.32)
Q9JI66	256	256	S	PDNGSPA	83.849	62.055	NLT(0.013	NLT(-16.3
P31211	292	292	Y	IDRWGKLI	20.663	20.663	LMT(1)PR(	LMT(20.66
P31211	285	285	T	AALSRDTI	20.663	20.663	LMT(1)PR(	LMT(20.66
Q62812	1940	1940	T	GDMPFVV	49.606	49.606	KGT(0.544	KGT(0.76)(
P19814	234	234	S	AGDSDFSI	73.464	73.464	GDKS(0.86	GDKS(8.36
D3ZHA0	2625	2625	S	VLVETVTK	67.993	67.993	GAS(0.979	GAS(19.71
D3ZXD8	12	12	S	____MADR	102.52	65.716	GGPAEEPS	GGPAEEPS
Q3T1J9	35	35	T	PEGSHQY	47.037	47.037	HAEAT(0.9	HAEAT(30
P19527	44	44	S	VRSGYST/	58.835	58.835	S(0.003)AY	S(-23.3)AY
P19332	574	574	T	VPMPDLK	54.982	54.982	IGS(0.43)T	IGS(-1.23)
P85125	173	173	S	YQDEVKLI	32.734	32.734	S(0.5)LKES	S(0)LKES(C
P85125	177	177	S	VKLPAKLS	32.734	32.734	S(0.5)LKES	S(0)LKES(C
Q63269	1842	1842	S	KGRVSSFS	80.706	80.706	Y(0.008)S(	Y(-20.69)S
Q569C0	121	121	S	CWKVRQF	147.07	128.85	RES(1)QT/	RES(38.4)C
P02688	94	94	Y	GLCHMYK	90.906	90.906	TTHY(0.99	T(-35.84)T
O55035	395	395	S	KGDKSELN	56.632	48.741	ENQRS(1)I	ENQRS(48
F1LP64	241	241	S	KDQNKAF	60.828	51.572	S(0.005)AS	S(-21.91)A
Q62639	175	175	S	FRRIILEAE	62.633	62.633	IDGAAS(1)	IDGAAS(6:
Q63356	1001	1001	S	SLYTSMAF	59.067	59.067	QQS(0.769	QQS(6.58)
P61150	212	212	S	HFVPKPIE'	62.924	35.204	EPS(1)LHE	EPS(35.2)L
P48679	403	403	S	RLSPSPTS(	79.511	79.511	AS(0.529)S	AS(0.72)S(
O08961	1186	1186	T	RDLTPETS	42.556	42.556	KGAQT(0.!	KGAQT(0).
P06907	209	209	S	LSAMEKG	81.338	81.338	S(0.001)S(	S(-32.54)S
P06907	120	120	S	PSWKDGS	28.765	28.765	DGS(0.006	DGS(-22.9
P06907	124	124	T	DGSIVIHN	28.765	28.765	DGS(0.006	DGS(-22.9

P06907	126	126	T	SIVIHNLDY	28.765	28.765	DGS(0.006	DGS(-22.9
Q5RKH1	258	258	S	PTLRRRSQ	59.265	59.265	ARS(1)PAI	ARS(59.26
P97573	245	245	S	LPSLESLQ	66.387	66.387	LFDQQLS(	LFDQQLS(
P32738	365	365	S	LKHMMTS	59.116	59.116	ADS(0.96)'	ADS(13.84
F1M3G7	765	765	S	SVVPQSEE	48.44	48.44	LEPDQVS(	LEPDQVS(
O54921	432	432	S	VLGHLSQT	122.94	122.94	GS(0.004)S	GS(-23.93
Q5RJQ4	330	330	S	DAQSGSQ	147.23	147.23	EHANIDA(	EHANIDA(
Q9EPH2	22	22	S	KAPRGDV	95.767	95.767	GDVTAEE/	GDVT(-78
Q65Z14	782	782	S	KCYPDIPN	91.584	91.584	SSILS(1)LI	S(-52.28)S
Q8VHK2	1035	1035	S	AIRRPPEG	76.064	60.91	PAS(1)PDI	PAS(60.91
P63326	146	146	S	AVPPGAD	37.083	37.083	AEAGAGS	AEAGAGS
P06907	210	210	S	SAMEKGK	63.816	63.816	SSKDS(0.0	S(-43.65)S
Q63358	1222	1222	S	CPKQVPIV	35.703	35.703	S(0.108)PS	S(-8.09)PS
Q9WU74	325	325	S	GGHSSQV	93.839	93.839	DVDGS(0.9	DVDGS(16
Q9R1Q2	353	353	S	ASKPSSPR	99.451	83.692	AEEKS(0.9	AEEKS(32.
Q8R4T5	93	93	S	PRRKGSGI	151.79	77.597	NFT(0.001	NFT(-32.7
Q6AYS6	409	409	S	KMLRRRV(	70.412	69.721	RS(0.008)D	RS(-20.98)
O89000	905	905	S	LAASKIREI	58.815	58.815	ACS(1)PLC	ACS(58.82
Q499S9	391	391	S	MVGRLTN	78.939	78.939	RIDS(1)YV	RIDS(46.98
Q62733	157	157	S	EKKLLKLRI	142.57	142.57	S(0.89)S(0.	S(12.1)S(-
O08984	70	70	S	KPLKSFKQ	61.161	51.359	S(0.002)GS	S(-25.38)G
Q8K1P7	1349	1349	S	FGRGSRHI	66.989	66.989	EVDYS(0.0	EVDY(-35.
P15865	146	146	T	PAGAAKK	63.091	53.166	ATGT(0.00	AT(-36.91)
P02688	72	72	S	SGKVPWL	50.473	50.473	S(0.003)PL	S(-25.6)PL
G3V6S8	316	316	S	SHSPLPAF	82.452	40.002	S(1)MS(1)I	S(40)MS(4
Q9WU74	454	454	S	ALDDINRF	98.233	83.168	S(0.979)S(	S(16.66)S(
Q63624	678	678	S	TITVGRPD	74.769	74.769	APS(1)PAF	APS(74.77
Q9WUD2	46	46	S	EPPPMESF	56.951	42.19	NS(0.863)S	NS(7.98)S(
F1LP90	602	602	S	RVPLKPYA	62.408	62.408	S(0.139)QS	S(-7.94)QS
Q91ZQ0	23	23	S	CDQRRGA	108.14	108.14	EQHNGS((	EQHNGS(2
P34900	188	188	S	KKDEGSYD	72.29	72.29	KPS(0.25)S	KPS(-4.77)
P26434	545	545	S	RKILIRRN(	73.985	73.985	S(0.006)S(	S(-22.04)S
Q3KR59	361	361	S	KPSSSSPV	62.813	62.813	CS(0.997)F	CS(24.86)F
O55164	798	798	S	PEEGYVSA	52.693	52.693	EDT(0.004	EDT(-23.8
P60669	627	627	S	PRPTSPRL	87.181	87.181	TLS(1)PVP	T(-34.4)LS
P50878	295	295	S	PMHKMM	64.199	64.199	ILKS(1)PEI	ILKS(64.2)I
Q63425	1312	1312	S	RVRLPRV(	31.876	31.876	GS(0.967)I	GS(14.66)I
Q5BJT0	75	75	S	STNAAASI	118.06	118.06	AS(0.002)S	AS(-26.28)
O08589	79	79	T	FNQQQRT	44.616	44.616	TGEPDEEE	T(-37.93)G
P22909	345	345	S	DSLPRRGI	104.67	104.67	RGPGAAG	RGPGAAG
P84092	236	236	S	VIEKQGK(	50.088	50.088	GTADET(0	GT(-38.17
P08050	372	372	S	AIVDQRPS	57.414	57.414	AS(0.551)S	AS(0.89)S(
Q9JJS5	246	246	S	KRDNPFFF	156.24	156.24	S(0.027)YS	S(-13.79)Y
D4A631	1076	1076	S	VKPRYISG	57.785	57.785	EGS(0.998	EGS(28.02
D3ZQL6	30	30	S	QCEGYRG	77.058	77.058	DLS(0.375	DLS(-1.95)
P60669	577	577	S	SPRVSRAS	43.246	43.246	QQS(0.5)S	QQS(0)S(0
O55035	356	356	T	TPSRSRSR	69.998	69.998	S(0.01)ET(	S(-19.87)E
D3ZXD8	16	16	S	MADRGGI	65.716	65.716	GGPAEEPS	GGPAEEPS
P97546	379	379	T	RPDEVPD/	44.425	44.425	T(0.095)NS	T(-7.74)NS
P0C5E3	170	170	S	KLGFPKKA	62.162	62.162	IAS(1)DEEI	IAS(43.52)
Q4KM98	131	131	S	EEIRAVGR	71.614	71.614	S(0.029)M	S(-15.18)M
Q5RKH1	387	387	S	NDRRSKQ	57.59	57.59	T(0.002)LS	T(-26.52)L
P35570	789	789	S	EPEEGARI	67.464	67.464	LS(0.023)S	LS(-15.6)S
P05197	57	57	T	KAGIIASAI	89.189	33.685	FT(0.988)D	FT(19.12)D
O70511	2216	2216	S	LSIKEKVK/	55.261	55.261	AS(0.447)S	AS(-0.92)S
P02688	41	41	S	RHGFLPRI	133.91	133.91	DTGILDS(1	DT(-86.68
P61980	379	379	S	QGGSGYD	126.51	126.51	GS(1)YGDI	GS(66.17)Y
P26453	371	371	S	QTLDEDD	41.644	41.644	GS(0.532)(	GS(0.55)G

P48679	404	404	S	LSPSPTSQ	115.57	55.337	AS(0.183)S	AS(-6.37)S
Q812D1	207	207	S	PRGRPKV	54.288	54.288	QPCPS(0.4	QPCPS(-1
Q642G4	282	282	S	VSNESPSS	50.077	50.077	DGHS(0.9	DGHS(25.6
D3ZAR1	197	197	S	KANQEGG	65.5	65.5	RDS(0.997	RDS(25.58
O35786	356	356	S	GPSSYPSH	115.7	115.7	MS(0.009)	MS(-20.34
Q9R050	320	320	S	NGSLGSG	31.883	31.883	NS(0.967)I	NS(17.46)I
P08050	328	328	S	YSAEQNR	164.41	164.41	MGQAGS(	MGQAGS(
Q921A3	107	107	S	GDEGQRH	70.816	70.816	SS(0.005)S	S(-35.66)S
P48679	414	414	S	RGRASSHS	116.73	116.73	ASSHSSQS	AS(-68.91)
Q9ERW3	222	222	T	PSLHDLTE	40.067	40.067	S(0.007)GS	S(-21.11)G
F1LP64	77	77	S	QVPKKDN	92.051	92.051	S(0.016)AS	S(-17.88)A
D3ZXD8	329	329	S	SASSSSSS	117.2	117.2	SSPS(0.00	S(-39.6)S(
O35786	355	355	S	TGPSSYPS	61.691	61.691	MS(0.985)	MS(18.04)
E9PTG8	514	514	S	GTSLSADL	106.2	62.823	ET(0.011)G	ET(-19.54)
Q9JKS6	617	617	S	PTATASKS	33.204	33.204	S(0.003)PV	S(-24.9)PV
P19332	573	573	S	PVPMPDL	58.699	58.699	IGS(0.972)	IGS(15.39)
Q62952	514	514	T	YDGPVFD	96.665	96.665	GGT(0.857	GGT(10.58
Q62733	384	384	S	AAGRPLEL	70.028	42.395	MEES(0.98	MEES(18.9
P97528	87	87	S	SMTYHYR	37.613	37.613	LDGGS(0.0	LDGGS(-1
Q5M7V8	320	320	S	GSGSLSPS	128.38	92.426	S(0.008)PV	S(-20.77)P
P48679	573	573	S	EDGDELLH	84.895	84.895	GS(0.05)H	GS(-12.47
P11497	79	79	S	SALQDGLA	105.86	105.86	S(0.009)S(	S(-20.49)S
P11345	621	621	S	IELLQHSLI	123.6	103.26	S(0.008)AS	S(-20.99)A
Q63433	377	377	S	PFLSRPAR	67.897	67.897	S(0.022)GS	S(-14.96)G
P97710	409	409	S	YLLRIKQKI	99.305	99.305	GST(0.003	GS(-45.43
B3DMA0	14	14	S	__MAAKQ	102.52	102.52	KHS(0.999	KHS(32.8)(
Q99P82	194	194	S	SGDAQSF	104.95	104.95	FYYS(0.09	FY(-80.76)
Q9WUD2	47	47	S	PPPMESPI	96.034	96.034	NS(0.017)S	NS(-17.52
Q9QZ48	331	331	S	TLLQQMN	79.013	79.013	AGDS(0.98	AGDS(16.9
P62243	130	130	T	SHYALPLG	61.477	61.477	GAKLT(1)P	GAKLT(61.
P49793	888	888	S	WVFKVSH	69.148	69.148	YGLQDS(1	Y(-53.01)G
Q5JCS6	1461	1461	S	HVLSKDDI	33.842	33.842	LMLPDS(1	LMLPDS(3
P13596	798	798	T	ESKEPIVEV	51.265	36.597	T(0.07)EEE	T(-11.23)E
P02688	68	68	S	PKRGSGKV	93.243	85.862	S(1)PLPSH	S(53.11)PL
Q5XIS8	59	59	S	LAVKRMY	104.42	104.42	AISAPT(0.0	AIS(-54.39
Q9JHY1	288	288	S	TAPGKKVI	96.544	58.09	VIYSQPS(0	VIY(-45.8)
P62718	24	24	T	EYKVVGRG	48.787	48.787	CHT(1)PPL	CHT(42.05
P49816	937	937	S	LSFDDTPE	51.252	51.252	S(0.748)T(	S(5.6)T(-5.
Q7TQ84	23	23	S	LVGATATT	46.592	46.592	ARS(1)NEI	ARS(46.59
Q6P773	146	146	S	RLEGNRGY	60.436	60.436	VTIVS(1)V	VT(-36.28)
P02688	190	190	S	LSKIFKLGG	95.815	86.833	S(0.002)GS	S(-26.15)G
P52481	309	309	S	RAQGQIRS	68.657	40.025	T(0.451)RT	T(-1.02)RT
Q1AAU6	1042	1042	S	QPPSEVTG	32.746	32.746	S(0.012)HT	S(-18.8)HT
Q62599	449	449	S	TRLDGERI	53.869	53.869	NNMS(1)F	NNMS(53.
P35280	185	185	S	KAKMDKK	59.372	59.372	KLEGNS(0	KLEGNS(-
O08678	414	414	S	STLQSPAH	60.91	60.91	S(0.126)IS(	S(-8.43)IS(
P49655	338	338	S	YVADFSLF	80.455	80.455	VAS(1)PGG	VAS(80.45
O35413	938	938	S	GSVLSLQD	45.829	45.829	S(0.149)Y(	S(-6.04)Y(
Q5XI74	110	110	S	KFEICVSSK	66.568	43.594	GNS(0.042	GNS(-13.5
Q4V8C3	146	146	S	RTSSSERV	82.417	82.417	RES(0.998)	RES(27.1)S
P62909	221	221	T	DHVSIVEP	179.93	98.943	DEILPT(0.0	DEILPT(-1
Q9Z327	537	537	S	MARSPMV	70.438	70.438	LVGQRS(1	LVGQRS(7
Q8CGU4	517	517	S	LALALVGT	69.984	69.984	IS(0.002)A	IS(-26.54)A
O70377	109	109	S	ESGKNYKA	198.87	198.87	ATWGDG(	AT(-103.0
Q5RJM0	244	244	S	MEINDDD	72.175	72.175	LPVS(1)PV	LPVS(36.2
Q5M7W5	799	799	S	ATSPSTLV	87.913	87.913	S(0.999)PS	S(33.31)PS
P97526	2798	2798	S	SPTTGHCI	48.44	48.44	HGS(0.83	HGS(7.16).
Q6PDU1	25	25	T	VEGMTSLI	139.97	139.97	T(0.993)S(	T(21.23)S(

Q9JIT3	203	203	S	ELDHRERE	76.586	41.967	ES(0.003)S	ES(-24.56)
O35431	305	305	T	EVKHSGD	38.634	38.634	T(0.5)RT(0	T(0)RT(0)P
O35431	307	307	T	KHSGDPQ	38.634	38.634	T(0.5)RT(0	T(0)RT(0)P
Q99JE6	1105	1105	S	EKKELQKII	128.03	128.03	NNS(1)ISE	NNS(35.42
Q5BJQ2	455	455	S	QQQQAV	67.169	67.169	APS(0.005	APS(-23.3
Q00715;Q 56;57		57	S	YIYKVLKQ	104.81	104.81	QVHPDT((	QVHPDT(-
Q63625	1205	1205	S	KHPHSPEK	100.73	38.093	EVS(1)PAF	EVS(38.09)
B2GV22	14	14	S	__MRRGEF	157.09	157.09	VAGGS(0.2	VAGGS(-3
P10888	58	58	S	DYPLPDVA	108.74	108.74	LLSAS(1)Q	LLS(-35.49
P61765	594	594	S	LDTLKKLN	75.854	45.433	LNKTDEEIS	LNKT(-33.
D4A1F2	515	515	S	RWGSVRR	48.741	48.741	RES(1)DIR	RES(48.74)
O88664	965	965	S	PMQGVPF	74.424	74.424	NS(1)PQA	NS(74.42)F
Q5XIB5	126	126	T	NPESPQRI	44.294	44.294	LS(0.435)P	LS(-1)PT(1
O08662	259	259	S	TLKRKTSSY	91.401	91.401	TSSVSSIS((	T(-68.3)S(-
D4AEC2	450	450	S	NAQSSTP	60.157	60.157	S(0.002)VS	S(-27.86)V
P41516	1371	1371	S	MPPKNTK	47.559	47.559	S(0.146)S((	S(-7.64)S(7
P15865	41	41	S	KAAGGAK	92.575	92.575	KAS(0.155	KAS(-6.94
P62997	26	26	S	ERESRSAS	94.309	35.204	S(0.446)GS	S(-1.03)GS
Q5U2W6	53	53	S	PAGPRGP(	95.483	95.483	S(1)APPM	S(95.48)AF
P02688	20	20	S	KRPSQRH(	147.58	97.631	YLATAS(0.	Y(-76.57)L
Q6P730	785	785	S	PGTRLRQ(	61.435	39.917	GDS(1)PEL	GDS(39.92
P11960	333	333	S	QPFLIEAM	91.355	91.355	IGHHS(0.9	IGHHS(16.
P62856	24	24	T	GRAKKGR(	25.319	25.319	GHVQPIR(	GHVQPIR(
Q9QYF3	602	602	T	MLPELFQD	103.97	103.97	AIS(0.247)	AIS(-4.81)
B2GV24	458	458	S	YKIKKVKKI	57.496	57.496	KDEDS(0.9	KDEDS(20
P84889	520	520	S	DPKSHKFV	80.438	80.438	LQSET(0.1	LQS(-36.0
P41516	1370	1370	S	KMPPKNT	49.189	49.189	S(0.897)S((	S(9.4)S(-9.
Q6AYK1	155	155	S	KSKPPKRD	51.927	41.283	S(0.998)PS	S(26.6)PS(
Q6AYK1	157	157	S	KPPKRDEK	51.927	32.606	S(0.997)PS	S(25.01)PS
P42930	15	15	S	_MTERRVF	107.56	107.56	S(0.094)PS	S(-9.82)PS
Q64632	1436	1436	T	RDYHSLTF	63.662	63.662	TDHSQS((	T(-54.53)D
Q63358	1220	1220	S	PSCPKQVI	66.546	66.546	S(0.927)PS	S(11.04)PS
D3ZXD8	327	327	S	APSASSSS	121.68	121.68	SSPS(0.018	S(-51.48)S
P02688	122	122	T	ENPVVHFI	113.76	102.71	T(1)PPPSQ	T(74.2)PPF
O55035	712	712	T	NEVKSSTL	55.064	55.064	T(0.89)RS(	T(9.06)RS(
Q9WUD2	82	82	S	PSQQEPD	65.305	65.305	LFS(1)VVS	LFS(41.51)
Q4V7C9	147	147	S	RERIGELGA	92.264	72.175	IGELGAPEY	IGELGAPEY
Q64632	1425	1425	S	HRMLSTSS	64.04	61.691	DYHS(0.99	DY(-48.73
P62755	247	247	S	RRLSSLRA	65.092	65.092	AS(0.042)T	AS(-10.49)
Q62896	50	50	S	LTESLRSKY	96.734	96.734	S(0.107)LS	S(-9.24)LS
Q68FR9	129	129	T	TLEKSSPTI	32.624	32.624	AT(0.011)A	AT(-18.91)
Q498D5	121	121	S	CIQDELGV	100.22	100.22	VTVHQVS	VT(-41)VH
Q62747	58	58	T	RKLGKRYK	60.196	60.196	NSLET(0.0	NS(-40.36
Q8VH46	549	549	S	QQKKVESA	56.087	56.087	T(0.162)AS	T(-7.14)AS
P54645	498	498	S	EAKSGTAT	98.582	98.582	SGS(0.031	S(-36.89)G
O08961	1187	1187	S	DLTPETSG	68.847	68.847	GAQT(0.21	GAQT(-5.6
Q7TSU1	356	356	S	NSQTNGIA	113.82	113.82	QS(0.01)LS	QS(-19.05
Q5M7V8	248	248	S	SASRASVS	53.683	53.683	ERS(1)PAL	ERS(53.68)
P47752	332	332	S	GGNPGHF	70.856	70.856	S(0.01)S(0.	S(-19.51)S
Q9Z340	378	378	S	KEQYEQLS	56.936	56.936	EMNNY(0.	EMNNY(-2
P06687	10	10	S	______MGD	84.213	84.213	DDKS(0.01	DDKS(-20
O88777	25	25	S	EEEVCDER	110.15	48.405	TSLMS(0.0	T(-39.35)S
O70531	35	35	S	AYGFPPEL	77.318	62.546	GS(0.813)S	GS(9.38)S(
Q8CG07	153	153	S	GLGKRPAA	71.263	71.263	RPAAAAA	RPAAAAA
Q6LED0	81	81	T	LPFQRLVR	74.133	74.133	EIAQDFKT	EIAQDFKT
Q5M7W5	786	786	S	LRPGPKTT	139.77	139.77	AT(0.014)S	AT(-18.35)
Q4KM77	56	56	S	RRRRASSL	71.349	60.398	RAQS(1)VI	RAQS(60.4
Q9WTR8	374	374	S	GPPHHPV	41.401	41.401	S(0.002)S((	S(-26.71)S

P11167	475	475	S	FDEIASGF	36.202	36.202	QGGAS(0.	QGGAS(-(
P06686	439	439	S	GLCNRAV	41.644	41.644	AGQENIS(	AGQENIS(
Q5M7V8	243	243	S	TYGTGSAS	123.96	123.96	ASVSDLS(	AS(-82.84)
O35821	14	14	S	__MAEMK	122.97	86.488	AEPAS(1)F	AEPAS(86.
P06494	703	703	T	TMRRLLQ	55.587	55.587	LLQET(0.0	LLQET(-29
P70615	302	302	S	NSARGGM	79.652	79.652	IES(0.94)LS	IES(12.07)I
P48679	429	429	S	SVTKKRKL	95.642	95.642	S(0.242)S(	S(-4.95)S(
Q63638	1177	1177	S	RTAASGPS	59.281	31.111	MPS(1)IPE	MPS(31.11
P34926	1307	1307	S	MTSDSSLT	86.557	64.069	S(0.005)PE	S(-21.61)P
P21396	383	383	S	IRKRKICEL	59.339	59.339	VLGS(0.97	VLGS(17.2
Q5M7W5	618	618	S	VKAADQK	141.54	128.32	STLPVDEG	S(-86.41)T
Q4V882	359	359	S	VLPSGPPI	46.66	46.66	TPVLPS(0.	T(-35.82)P
Q4V7E8	133	133	S	ASATTPLS	85.376	75.3	GS(0.877)(	GS(9.26)G
P34926	1763	1763	S	PEEEDKLT	72.399	72.399	S(0.009)PF	S(-20.59)P
Q5M7W5	506	506	T	VVTLPETK	104.41	104.41	VTEFNNV	VT(-44.99)
P34064	56	56	S	QTSEGVCI	104.41	104.41	IT(0.016)S(	IT(-17.94)S
P50137	287	287	T	QEIYSQVC	95.624	95.624	ILAT(1)PP(	ILAT(73.31
Q2EJA0	95	95	T	FFKPPEPKS	140.12	140.12	QAS(0.152	QAS(-7.47
Q1AAU6	1056	1056	S	LSPNVQSI	87.991	87.991	QAS(0.972	QAS(15.42
Q05764	612	612	T	KSTPASPV	45.863	45.863	AGT(0.547	AGT(0.85)I
P15146	1359	1359	T	EAQAEPKI	55.994	40.428	DGS(0.997	DGS(26.56
Q63425	131	131	S	EMKGPRA	117.2	54.023	LNIQS(0.9	LNIQS(17.
Q6P9V9	334	334	T	YRGDVVP	63.473	43.03	DVNAAIAT	DVNAAIAT
P16884	654	654	S	EAKSPAEV	139.86	39.531	S(1)PVEAK	S(39.53)PV
Q5QD51	585	585	S	PLEAPQDC	98.491	98.491	GPLEAPQI	GPLEAPQI
Q4G091	377	377	T	SRSLPNRF	17.489	17.489	PS(0.685)K	PS(0)KMFF
Q4G091	376	376	T	RSRSLPNR	17.489	17.489	PS(0.685)K	PS(0)KMFF
Q00566	421	421	S	LSSSICKEE	31.036	31.036	AGS(0.505	AGS(0.08)I
Q66HG9	238	238	S	VSFQPLPR	44.238	44.238	T(0.267)NI	T(-4.33)NI
Q924C3	25	25	S	QGPRHGF	43.497	43.497	ELES(0.878	ELES(8.58)
P34926	1784	1784	S	PEMTGQR	87.952	87.952	VPS(0.001	VPS(-31.9
Q62835	180	180	S	QEIQRRPF	91.889	91.889	QPASLHG	QPAS(-40
P15205	1138	1138	S	EPTPMDEI	32.172	32.172	DVMS(0.5	DVMS(3.4
P19332	191	191	S	DVERSHPA	98.281	98.281	SHPASELL	S(-85.05)H
Q4V8H8	468	468	S	YDEIFYNLA	67.364	67.364	YDEIFY(0.0	Y(-49.07)D
Q5XII5	476	476	S	LATASGQI	76.106	76.106	MFPEPT(0	MFPEPT(-
P34926	526	526	S	AAPPAAV	65.425	65.425	ELALS(0.8	ELALS(6.2
P23348	170	170	S	PPPPGPPC	74.732	74.732	FS(0.033)I	FS(-14.7)I
P15205	824	824	S	ASGPAKEI	64.224	56.563	S(0.022)LN	S(-19.45)L
Q4V893	106	106	S	FFGPALPP	37.228	37.228	QDDS(1)P	QDDS(37.2
P15146	884	884	S	DMGYCVF	86.507	86.507	Y(0.001)T(	Y(-32.18)T
Q9JK11	766	766	S	VSETVAQI	54.259	54.259	LS(0.028)A	LS(-15.48)
P26431	707	707	S	AHKLDSPT	68.83	68.83	IGS(1)DPL	IGS(53.93)
Q641Y8	475	475	T	GKNHIRTD	40.012	40.012	DNT(0.54)	DNT(0.72)
Q925N3	141	141	S	DAEQRAQ	96.487	96.487	GPET(0.01	GPET(-16.
O35346	913	913	S	YNEGVKPV	44.047	44.047	LQPQEIS(	LQPQEIS(2
Q5QD51	350	350	S	SEEQEPAE	19.379	19.379	T(0.01)EPA	T(-17.1)EP
Q9QYM0	43	43	S	GQHGDRE	65.184	64.52	S(1)LECQD	S(59.81)LE
B2DD29	586	586	S	MQVPTAE	59.359	59.359	MQVPTAE	MQVPT(-3
Q5M7W5	509	509	S	LPETKVTE	93.407	93.407	VTEFNNV	VT(-65.73)
Q91Z79	142	142	S	HERSLRMT	40.81	40.81	QAQS(0.9	QAQS(18.5
P19945	307	307	S	AAAPAKV	99.838	77.527	EES(0.003)	EES(-25.98
Q4V8H8	470	470	S	EIFYNLAPA	59.814	59.814	YDEIFYNLA	Y(-51.15)D
Q9ERE6	981	981	S	LKEQLKAA	70.983	70.983	AAT(0.016	AAT(-17.7
P70501	660	660	S	SPPRGLVA	41.521	41.521	GLVAAY(0	GLVAAY(-
Q3SWT4	183	183	S	QASDSESI	101.39	101.39	IS(0.961)D	IS(14.74)D
P34926	1767	1767	S	DKLTRSPF	82.99	72.399	S(0.009)PF	S(-20.59)P
Q63488	423	423	S	SSAPEDSE	40.727	17.917	LVGDT(1)V	LVGDT(17

P12839	751	751	S	VEGKEEEE	48.44	48.44	GS(1)GQE	GS(48.44)
Q9Z1T4	906	906	S	NIGEKNEN	117.86	117.86	LGDS(1)L	LGDS(45.6
Q9JHB5	33	33	S	FPHNQRR	58.32	58.32	DAS(0.008	DAS(-19.3
P16884	508	508	S	SPPAEEAA	76.143	30.956	S(1)PVKEE	S(30.96)PV
Q5XIT1	162	162	S	NKSKKLIG	69.954	69.954	T(0.43)S(0.	T(-1.22)S(
Q63425	133	133	S	KGPRAKV	117.93	105.14	LNIQS(0.0	LNIQS(-18
Q9QYM0	41	41	T	IPGQHGD	65.184	65.184	T(0.5)RS(0	T(0)RS(0)L
P26431	801	801	S	GPSDSPGS	25.408	25.15	CLS(1)DPC	CLS(25.15)
P16884	684	684	S	TVKSPAEA	91.969	91.969	SPVEVKS(	S(-55.78)P
P19944	104	104	S	AEEKKVEA	116.35	116.35	KEES(0.00	KEES(-25.5
P70580	181	181	S	HVGKLLKE	218.44	218.44	EGEEPTVY	EGEEPT(-4
P11530	3616	3616	S	TTVSSPST	143.28	143.28	S(0.001)DS	S(-30.25)D
P16884	762	762	S	EAKSPAEA	89.43	57.434	S(1)PVEVK	S(57.43)PV
P16884	690	690	S	EAKSPVEV	123.64	93.011	SPASVKS(	S(-58)PAS
Q5U2M8	168	168	S	IRGRSQNS	51.487	51.487	VLLAEDS(	VLLAEDS(
P16884	666	666	S	TVKSPVEA	128.9	128.9	SPAEVKS(	S(-56.81)P
Q62632	163	163	S	DKYFKSFD	87.352	87.352	SFDNGDS	S(-50.55)F
P31016	295	295	S	TDYPTAM	42.599	42.599	RY(0.009)S	RY(-20.45)
Q9ESN0	581	581	S	NLFEDNM	62.582	62.582	HNLFEDN	HNLFEDN
Q8VBU2	330	330	T	GYMASSC	28.202	25.619	S(0.539)RT	S(-0.72)RT
P0DJJ3	265	265	S	PLPPKAVP	89.47	89.47	T(0.196)GS	T(-6.03)GS
P16884	528	528	S	EAKSPAEA	83.039	61.821	S(1)PAEAK	S(61.82)PA
P16884	534	534	S	EAKSPAEA	126.63	70.41	S(1)PAEVK	S(70.41)PA
Q4V882	419	419	S	ESRDSAQ	104.06	104.06	S(0.5)PS(0	S(0)PS(0)P
Q4V882	417	417	S	PMESRDS	104.06	104.06	S(0.5)PS(0	S(0)PS(0)P
Q6P9T8	55	55	T	SDLQLERI	58.831	58.831	INVYY(0.0	INVY(-37.
P34926	1742	1742	S	PLPQKGLE	88.836	24.594	WLAES(1)	WLAES(24
Q5QD51	370	370	S	KVELPLED	110.4	110.4	VELPLEDQ	VELPLEDQ
P04466	15	15	S	_MAPKKA	96.052	50.498	AAAEGS(0	AAAEGS(0
P16884	768	768	S	EAKSPVEV	52.555	43.282	S(1)PVKEG	S(43.28)PV
P15205	1056	1056	Y	AVVDKAA	54.805	54.805	AAEAGVT	AAEAGVT
Q6AYH5	83	83	S	DFSDRIGK	81.665	81.665	T(0.088)GY	T(-10.13)G
P10362	558	558	S	EQLEQAIK	46.481	46.481	EHLGQGS	EHLGQGS
P10362	557	557	S	EEQLEQAI	46.481	46.481	EHLGQGS	EHLGQGS
P11497	25	25	S	TLELNQHS	77.045	77.045	FIIGS(0.02	FIIGS(-15.
P15205	1070	1070	S	QYGFLGTI	93.556	93.556	QPGVQS(	QPGVQS(
P16884	714	714	S	GAKSPAEA	104.17	92.773	SPVVAKS(	S(-62.6)PV
P0DJJ3	371	371	S	GSAGPPG	84.508	63.682	HVPS(1)PL	HVPS(63.6
P16884	414	414	S	RKLLEGEE	77.506	50.787	IGFGPS(0.	IGFGPS(24
Q6MG08	109	109	S	ERVLMERI	75.797	66.023	QLS(0.003	QLS(-25.5
Q925Q9	156	156	S	NGKTGMF	133.38	133.38	ELS(0.972)	ELS(15.33)
P37285	524	524	S	VLNDPEN	80.746	80.746	S(0.317)RE	S(-3.33)RE
P16884	672	672	S	EAKSPAEV	96.059	28.58	S(1)PAEAK	S(28.58)PA
P19527	67	67	S	SVRRSYSS	91.079	91.079	SYSSSSGS	S(-45.61)Y
P16884	630	630	S	EAKSPAEA	89.911	89.911	SPASVKS(	S(-53.57)P
Q2TL32	457	457	S	LRVRDILSI	49.423	49.423	T(0.002)KE	T(-26.7)KE
Q63560	681	681	S	PKDQAPV	45.611	45.611	GQS(0.79	GQS(5.83)
Q5QD51	274	274	S	EKQEKEPT	84.159	84.159	S(0.009)PE	S(-18.56)P
O88588	428	428	S	IGSLNSKG	66.387	66.387	DT(0.038)	DT(-13.22
P15205	1332	1332	S	SQSVTGSA	99.515	99.515	TLEVVSPS	T(-86.94)L
Q80X08	387	387	S	RGQPAQC	110.9	110.9	GQPAQGF	GQPAQGF
Q5QD51	1354	1354	S	SSERGKAL	57.348	57.348	ALGSLGGS	ALGS(-33.
P16884	702	702	S	SVKSPSEA	92.457	28.624	S(1)PAGAK	S(28.62)PA
P34926	894	894	S	EATQGLDY	114.63	103.18	TEATQGLI	T(-80.87)E
P16884	708	708	S	EAKSPAGA	108.17	27.742	S(1)PAEAK	S(27.74)PA
P23565	474	474	S	GASKEVTK	109.03	109.03	VGES(1)FE	VGES(47.2
Q5XIR9	98	98	S	IQDQDVLI	61.593	61.593	RAPS(1)PI	RAPS(61.5
O55164	1270	1270	S	NPFAESLQ	84.169	54.343	APS(0.958	APS(13.87

P16884	846	846	S	KTPAKEEA	117.08	38.511	RPADIRS(1	RPADIRS(3
P16884	588	588	S	EAKSPAEA	106.32	82.543	SPAEVKS(0	S(-52.11)P
Q80X08	531	531	S	NPKLVSET	117.38	117.38	GLFS(1)DE	GLFS(49.62
P57097	561	561	S	AKKSFCRR	38.583	38.583	AIELT(1)LC	AIELT(38.5
P57097	558	558	T	NYRAKKSF	38.583	38.583	AIELT(1)LC	AIELT(38.5
P57097	565	565	S	FCRRAIELT	38.583	38.583	AIELT(1)LC	AIELT(38.5
O88658	1057	1057	S	NQSDFPSA	88.948	88.948	S(0.001)GI	S(-30.25)G
O70511	855	855	S	HKMNVPE	80.061	80.061	MNVPETM	MNVPET(-
Q5FVH4	30	30	S	RAEGEEKT	39.163	39.163	T(0.005)LA	T(-21.91)L
P16884	600	600	S	EVKSPATV	116.19	29.299	S(1)PGEAK	S(29.3)PGE
P16884	902	902	T	AKEPPKKV	57.802	45.33	KVEEEKT(0	KVEEEKT(-
Q63425	7	7	S	________M	117.99	59.35	S(1)AEELR	S(59.35)AE
P37285	462	462	T	GEYGGWY	36.022	36.022	ACKVDS(0	ACKVDS(-
Q7TNK6	93	93	Y	GHGKSPEE	26.729	26.729	S(0.427)PE	S(0)PEELY(
P97839	973	973	S	KRLLAAKR	44.648	44.648	QNS(0.573	QNS(3.43)
P16884	651	651	T	SPAEAKSP	46.578	44.601	S(0.987)PA	S(18.45)PA
P34926	598	598	S	LEGEHVEF	118.77	40.589	EVVPDS(1	EVVPDS(4
P97603	1163	1163	S	PDPNPVM	29.591	29.591	NS(0.953)(	NS(13.48)(
Q6IMY8	58	58	S	DNEAGGR	24.893	24.893	PAMEPGN	PAMEPGN
P0DJJ3	335	335	T	PEHVTPEL	37.337	37.337	EKVVT(0.8	EKVVT(8.9
P16884	756	756	S	EAKSPAEA	57.434	57.434	S(1)PVEVK	S(57.43)PV
Q6JE36	362	362	S	SEGPRSRS	75.088	75.088	SHTS(0.00	S(-68.05)H
P26284	232	232	S	PCIFICENN	91.265	91.265	YGMGT(0.	Y(-75.62)G
P16884	827	827	S	EEAKSPEK	132.17	132.17	TLDVKS(1)	T(-49.09)L
Q9JK11	169	169	S	PAAPPSTF	78.319	71.903	RGS(0.782	RGS(5.89)(
P16884	678	678	S	EVKSPVTV	97.551	28.58	S(1)PAEAK	S(28.58)PA
Q6RJR6	111	111	S	DGKDPLV	48.44	48.44	T(0.008)LD	T(-20.82)L
P15205	1205	1205	S	GRDYNAS	114.2	114.2	DYNAS(0.0	DY(-72.26
P16884	660	660	S	EVKSPATV	94.487	39.531	S(1)PVEAK	S(39.53)PV
Q5QD51	614	614	S	SFKKMVTF	131.48	131.48	RPS(0.996)	RPS(24.14)
Q9JK11	107	107	S	GPLPAAPF	153.19	153.19	GPLPAAPF	GPLPAAPF
Q2KJ09	433	433	T	KDDSYMK	22.636	22.636	DDS(0.219	DDS(-10.0
Q2KJ09	431	431	S	EEKDDSYM	22.636	22.636	DDS(0.219	DDS(-10.0
Q2KJ09	434	434	S	DDSYMKT	22.636	22.636	DDS(0.219	DDS(-10.0
Q63430	59	59	S	RDLPSPLP	90.601	90.601	T(0.005)YS	T(-23.11)Y
P16884	516	516	S	SPEKETKSI	83.106	30.956	S(1)PVKEE	S(30.96)PV
P16884	594	594	S	EAKSPAEV	118.37	29.299	S(1)PGEAK	S(29.3)PGE
P16884	852	852	S	EAKRPADI	66.893	39.625	S(1)PAKEE	S(39.62)PA
P55260	12	12	S	___METKG	60.334	60.334	AAS(0.998	AAS(27.78
P16884	782	782	S	KSPVKEGA	117.79	117.08	SLAEAKS(1	S(-85.85)L
P16884	816	816	S	KSPEKAKS	83.54	44.616	EEAKS(1)P	EEAKS(44.
P29067	164	164	S	AFCAKSIEI	59.35	44.318	NS(1)VRLI	NS(44.32)V
Q5U318	116	116	S	RIPSAKKYI	139.67	72.2	QPS(1)EEE	QPS(72.2)E
P16884	808	808	S	EIKPPAEVI	80.522	41.399	S(1)PMKEE	S(41.4)PM
A0A096M	85	85	S	QKGDLFKA	87.326	87.326	AEYVFIVD	AEY(-53.1
P15146	610	610	S	LSDSRGNA	82.59	82.59	GNAQESL	GNAQES(-
P16086	1031	1031	S	VPAAYVKI	118.9	118.9	LDPAQS(0	LDPAQS(0
Q5U2M8	801	801	S	PSDHRLFS	52.541	52.541	LFS(0.002)	LFS(-24.47
P0C1X8	625	625	S	PTIQGQKV	61.409	43.813	VGS(0.43)I	VGS(-1.24
P34926	1306	1306	S	FMTSDSSL	43.955	43.955	S(0.019)PE	S(-14.19)P
P16884	860	860	S	RSPEQVKS	78.326	46.319	EEAKS(0.9	EEAKS(30.
P16884	696	696	S	EVKSPASV	87.824	28.624	S(1)PAGAI	S(28.62)PA
P61808	49	49	S	CYLRLQRI	78.078	78.078	ISQS(0.001	IS(-41.62)(
P15205	1436	1436	S	PFEGKNGI	55.881	55.881	QGFS(0.19	QGFS(-6.2
P97876	85	85	T	LRREIAKLK	23.987	23.987	EELRHLT(1	EELRHLT(2
Q9QUL6	739	739	S	YRVRKFLA	68.657	68.657	EEGAS(1)F	EEGAS(68.
P16884	546	546	S	EAKSPAEV	69.261	69.261	SPAEVKS(1	S(-42.23)P
P37285	460	460	S	SFGEYGGV	116.24	116.24	VDS(0.979	VDS(16.74

P34926	1254	1254	S	APVSIPEPI	91.967	53.1	AT(0.047)V	AT(-11.19)
P15146	521	521	T	AVTDAAM	74.987	74.987	VT(0.561)S	VT(1.06)S(
O55170	26	26	S	ELSPVGSE	117.94	117.94	CLS(0.295)	CLS(-2.58)
P16884	624	624	S	EVKSPVEA	74.46	74.46	SPAEAKS(	S(-43.23)P
P16884	832	832	T	PEKAKTLD	74.12	58.37	T(1)PAKEE	T(58.37)PA
P16884	692	692	S	KSPVEVKS	67.115	30.107	S(0.205)PA	S(-8.82)PA
B2GV05	624	624	S	ENPLKRGL	62.475	62.475	GLVAAY(0	GLVAAY(-
Q9JI66	245	245	S	TVSSASRN	89.344	89.344	MFSNPDN	MFS(-61.5
P16884	540	540	S	EAKSPAEA	113.35	70.41	S(1)PAEVK	S(70.41)PA
P15205	609	609	S	GKVESKPS	20.625	20.625	EVPS(0.5)K	EVPS(0)KE
Q9EPJ0	223	223	S	EDEEAESP	114.89	61.344	T(0.004)S(	T(-24.2)S(
P31000	459	459	S	IKTVETRD	36.029	31.42	DGQVINET	DGQVINET
P31000	458	458	T	LIKTVETRD	36.029	31.42	DGQVINET	DGQVINET
P34926	872	872	S	TEETGKSS	50.831	50.831	SSLLLDT(0	S(-42.44)S
P13668	25	25	S	ELEKRASG	60.093	60.093	ASGQAFEI	AS(-55.29)
D3ZML2	394	394	S	PERKSMEV	52.03	52.03	SMEVLS(0	S(-35.73)M
P16884	669	669	T	SPVEAKSP	36.677	34.009	S(0.053)PV	S(-15.31)P
Q5QD51	685	685	S	ICVGSSKK	151.92	151.92	ASS(0.038	AS(-42.53)
P02401	102	102	S	APAAAEEI	46.249	46.249	KEES(0.74)	KEES(4.55)
Q4V8B0	197	197	S	STESSIRPA	115.87	115.87	VVS(0.003	VVS(-24.3
P16884	618	618	S	EAKSPAEV	81.311	81.311	SPVEAKS(	S(-43.76)P
Q9JKC9	565	565	S	KTEEDDFC	102.48	102.48	S(0.544)GS	S(0.77)GS(
P34926	1897	1897	S	REGEGGA	77.464	77.464	EGEGGAG	EGEGGAG
Q9WU70	693	693	S	PYRREPRS	31.771	31.771	QPS(0.928	QPS(11.62
P04466	16	16	S	MAPKKAK	96.052	96.052	AAAEGS(0	AAAEGS(0
P19527	473	473	S	TIEATKAEI	101.53	58.073	DEPPS(1)E	DEPPS(58.
P13383	566	566	S	IEGRTIRLE	30.998	30.998	LELQGPR(	LELQGPR(
Q9ESM0	139	139	S	RSGSGSDI	76.378	76.378	AS(0.029)L	AS(-14.17)
Q9WTQ1	203	203	S	FKIPNNCS	39.863	39.863	RLS(0.968)	RLS(15.27)
Q5M7W5	546	546	T	ADLHSGTI	105.64	105.64	NADLHSG	NADLHS(-
Q64548	348	348	S	SSGTEPSA	44.252	44.252	GS(0.538)V	GS(0.66)VS
P35281	73	73	T	IKLQIWDT	37.696	37.696	FHT(0.92)I	FHT(12.46
P12839	545	545	S	EEDEGVKS	64.65	64.65	SDQAEEG	S(-49.77)D
P15205	2065	2065	S	YSYETSDR	58.955	33.947	CY(0.005)T	CY(-22.33)
P08721	270	270	S	IDSAERSD	55.724	55.724	SDAIDS(0.	S(-43.34)D
Q5PQM2	590	590	S	GTEPRPSS	67.827	67.827	AAS(0.997	AAS(24.66
P16884	417	417	S	LEGEECRI(	86.455	66.215	IGFGPS(0.(	IGFGPS(-1
Q9ESB5	15	15	S	_MEDSRET	77.19	77.19	ETSPS(0.0(	ET(-33.7)S
Q5M7W5	520	520	S	VTPLSEEE'	136.2	136.2	VTEFNNV'	VT(-108.5'
P12839	507	507	S	EEKEEEPE'	79.693	58.172	S(0.001)PV	S(-28.63)P
P34926	1574	1574	S	SKETVLDQ	99.366	32.981	ADS(1)VE(	ADS(32.98
Q63544	120	120	S	QDQEAKE	84.566	84.566	EQEEGEEA	EQEEGEEA
Q5M7W5	978	978	S	SQKLNFKE	86.625	62.303	VGS(1)LDI	VGS(52.65
B5DFC8	45	45	T	NYGKQPL	23.799	14.254	QPLLLS(0.	QPLLLS(0)
Q64548	210	210	S	IDITRPQEA	77.847	31.233	GQEEQS(1	GQEEQS(3
P15205	1788	1788	T	PKSDISPLT	63.69	63.69	ES(0.135)S	ES(-8.56)S
P02091;P1	140;140	140	S	PCAQAAF	89.629	69.03	VVAGVAS	VVAGVAS
Q6AXU6	74	74	S	PPSWAKS.	86.838	86.838	SAGGRED	S(-46.47)A
P16884	732	732	S	EAKSPAEA	147.9	147.9	SPAEAKPF	S(-112.46)
P16884	606	606	S	TVKSPGEA	68.576	28.806	S(1)PAEVK	S(28.81)PA
P15205	1781	1781	T	LEGEKLSP	137.98	137.98	SDISPLT(1	S(-89.89)D
P15205	884	884	S	YVIQKETE'	81.18	81.18	GS(0.412)A	GS(-1.54)A
P37285	521	521	S	VAEVLND	67.964	67.964	S(0.5)RES(	S(0)RES(0)
P15205	1317	1317	S	HCASPEEK	126.45	76.402	T(0.001)LE	T(-26.55)L
P16884	750	750	S	EAKSPAEA	44.632	43.162	S(0.11)PAE	S(-10.47)P
Q56B11	757	757	T	HRAGSGE	54.66	54.66	AGSGEDP'	AGS(-36.9
Q80X08	720	720	S	AATKKESII	62.605	62.605	VPLLFS(0.9	VPLLFS(14
P02091	51	51	S	YPWTQRY	129.12	79.837	YFDSFGDI	Y(-48.88)F

P15205	2027	2027	S	ESESYSYET	122.96	56.247	T(0.06)T(0.	T(-11.24)T
P34926	1134	1134	S	EPQKDEVI	72.495	64.127	FT(0.005)D	FT(-23.49)
Q66HA8	810	810	S	NVCEPVV	65.5	54.539	IES(1)PKLE	IES(54.54)I
Q9QYU1	54	54	S	SPTISAPD.	67.993	67.993	S(1)PGDT/	S(38.52)PC
P15146	1051	1051	S	KADQGLD	75.669	26.627	KDDQS(1)	KDDQS(26
P35465	222	222	S	PLPVTPTR	149.62	149.62	DVAT(0.0(	DVAT(-23
Q4KLH6	487	487	S	EAPVTSKC	81.215	40.002	S(1)APPLP	S(40)APPL
P15205	1618	1618	S	KEECPRPN	85.837	85.837	PMSISPPD	PMS(-56.5
Q9ERE6	1020	1020	S	DRSCVTR(	72.417	72.417	S(0.5)KS(0	S(0)KS(0)V
Q9Z340	668	668	T	KANQEAN	18.161	18.161	RS(0.667)N	RS(0)MS(0
Q62785	60	60	S	DGASGDP	110.74	110.74	S(0.027)LD	S(-15.53)L
P16884	582	582	S	EVKSPAEA	63.827	63.827	S(1)PAEVK	S(43.09)PA
P08733	15	15	S	_MSPKKAI	84.68	84.68	LEGGS(0.1	LEGGS(-7.
P15205	1315	1315	S	EEHCASPE	140.66	99.269	T(0.007)LE	T(-21.27)L
Q62785	63	63	S	SGDPKKEK	98.165	62.739	S(0.045)LD	S(-13.77)L
P16884	591	591	T	SPAEAKSF	46.408	46.408	S(0.001)PA	S(-28.92)F
P16884	612	612	S	EAKSPAEA	118.37	28.806	S(1)PAEVK	S(28.81)PA
O35430	264	264	S	SPEKEAEF.	100.19	100.19	MDS(1)YE	MDS(39.08
P15205	1009	1009	S	EEDMDEA	27.89	27.89	GEAEQS(1	GEAEQS(2
P15146	939	939	S	AAGRVKD	67.385	67.385	EAS(0.986	EAS(19.49)
P15205	1321	1321	T	PEEKTLEV	99.269	99.269	T(0.007)LE	T(-21.27)L
P15205	1874	1874	S	GDFNYAY	187.79	187.79	TPGDFNY.	T(-130.64)
P15205	1257	1257	S	RLSPAKSP	109.83	109.83	SPSLSPS(0	S(-65.07)F
P16884	687	687	S	SPAEAKSF	47.942	39.685	S(0.196)PA	S(-5.59)PA
P27321	175	175	T	DKVVVKKI	57.347	57.347	S(0.03)LT(	S(-14.32)L
P15205	1626	1626	T	SISPPDFSF	74.841	74.841	S(0.019)RT	S(-17.06)R
P62521	200	200	S	QPSKGDR	33.961	33.961	EY(0.003)V	EY(-24.53)
P12839	793	793	S	SDDKVVV	74.15	53.569	IT(0.124)S(	IT(-8.49)S(
B5DF41	219	219	S	SPARSLTR	35.678	35.678	S(0.006)S(	S(-21.81)S
P34926	1022	1022	S	SPVEDKSE	84.297	84.297	DFQEDS(1	DFQEDS(3
P12839	721	721	S	KAEEEGGS	179.58	129.24	EKAEEEGC	EKAEEEGC
P12839	761	761	T	TQEKGSG(	23.842	23.842	GVVT(0.5)	GVVT(0)N
Q5XIL2	189	189	Y	GCLLPNQ	14.015	14.015	GCLLPNQ	GCLLPNQ
Q5XIL2	182	182	Y	CPCCFGR(	14.015	14.015	GCLLPNQ	GCLLPNQ
P16884	880	880	S	ETRTEKVA	136.01	94.85	S(1)PVEEV	S(94.85)PV
Q68FR9	119	119	S	AISKLEVRI	104.17	104.17	LSTLEKS(0	LS(-86.62)
P34926	1148	1148	S	LSPEDAES	72.495	72.495	FT(0.005)D	FT(-22.14)
P15205	1905	1905	T	QAHDVGC	146.13	146.13	T(0.622)IKS	T(2.2)IKS(-
P16884	744	744	S	EAKSPAEA	43.523	43.523	S(0.945)PA	S(12.25)PA
P15146	1024	1024	S	TTKETAPE	98.165	98.165	GLS(0.128	GLS(-8.32)
P16884	720	720	S	EAKSPVVA	120.23	48.948	S(1)PAEAK	S(48.95)PA
Q66H20	308	308	S	AFAKETSL	51.963	51.963	ET(0.002)S	ET(-27.57)
O35923	3322	3322	S	SASTEARF	29.689	29.689	ES(0.997)L	ES(24.8)LR
P34926	1643	1643	S	PVGGQKE	121.47	121.47	EPVPAWE	EPVPAWE
P15205	1148	1148	S	PRDVMSD	155.76	155.76	DVMSDET	DVMS(-10
P02401	79	79	S	SVPAGGA	68.368	68.368	LASVPAG(	LAS(-36.0(
P15205	1496	1496	T	ALALDERK	129.93	89.721	KLGGDGS	KLGGDGS
P15205	1393	1393	S	PDSESPIEI	45.22	29.444	S(0.947)PF	S(17.82)PF
P19332	711	711	S	KTDHGAE	114.76	45.257	S(0.097)PV	S(-7.94)PV
Q5EB94	247	247	S	EANAEVM	35.944	35.944	KLY(1)S(1)	KLY(35.94)
Q5EB94	249	249	Y	NAEVMRE	35.944	35.944	KLY(1)S(1)	KLY(35.94)
Q5EB94	246	246	Y	QEANAEV	35.944	35.944	KLY(1)S(1)	KLY(35.94)
O35314	155	155	S	HPSNQQV	53.683	42.743	IRHS(1)EEI	IRHS(42.74
P19332	714	714	T	HGAEIVYK	45.257	40.067	S(0.013)PV	S(-21.09)F
P34926	1460	1460	T	RDSEEKDK	77.597	28.283	DRT(1)PEE	DRT(28.28
P15205	1382	1382	S	RSSISPMD	124.66	84.942	S(0.112)S(	S(-8.47)S(
Q8CGZ2	313	313	S	KPRERAED	53.625	53.625	AEDSTGT(	AEDS(-35.
P15205	1765	1765	S	RDMSLYA	82.029	82.029	VQS(1)LE(	VQS(82.03

P15205	1494	1494	S	QSALALDI	151.39	151.39	LGGDGS((	LGGDGS(7
Q64548	350	350	S	GTEPSAAE	170.18	170.18	GSVS(1)ED	GS(-33.91
P70483	245	245	S	VLDNFKFL	38.724	38.724	FLENAAAI	FLENAAAI
P15205	1371	1371	S	FEFTEAKD	124.66	124.66	S(0.018)S(	S(-17.17)S
P34980	293	293	T	MLKMIFN	28.212	28.212	MIFNQMS	MIFNQMS
Q6P7P5	413	413	S	KKFVEWLI	42.001	37.954	NAEEES(1)	NAEEES(3
P35465	224	224	T	PVTPTRD\	46.578	46.578	DVAT(0.09	DVAT(-14
P15205	1150	1150	S	DVMSDET	133.38	133.38	DVMSDET	DVMS(-97
P15205	1925	1925	T	CDSGYSYE	108.08	101.75	T(0.053)T(	T(-12.28)T
Q66H76	132	132	T	VYSFPNKC	41.562	41.562	S(0.042)AE	S(-11.05)A
Q5QD51	1352	1352	S	MPSSERGI	117.53	117.53	ALGS(0.00	ALGS(-27.
P12839	609	609	S	IKVEKPEK/	94.85	26.541	S(1)PVEEV	S(26.54)PV
P15205	1908	1908	S	DVGGYYY	128.98	128.98	T(0.001)IKS	T(-25.74)II
P15205	1248	1248	S	LDIKDVSD	39.506	25.803	LS(0.131)P	LS(-8.01)P
P15205	1274	1274	T	IEKTPLGEI	126.09	101.75	SVNFS(0.0	S(-52.57)V
Q5U1Z0	976	976	S	KDVENPD	47.088	47.088	S(0.923)PF	S(10.88)PF
Q5QD51	1571	1571	S	CQDETPS/	133.47	62.546	GLAS(1)PD	GLAS(62.5
P15205	1624	1624	S	PMSISPPD	41.383	37.426	S(0.5)RT(0	S(0)RT(0)P
P12839	713	713	S	QQQEKVK	178.22	27.055	VKEKAEEE	VKEKAEEE
P12839	604	604	S	AIKEEIKVE	72.643	30.416	AKS(1)PVF	AKS(30.42
P15205	1790	1790	S	SDISPLTPI	110.31	110.31	ESS(0.002)	ES(-32.98)
O35314	100	100	S	LLRDPSDA	76.471	76.471	WAS(0.122	WAS(-7.04
P70580	178	178	T	KYHHVGK	36.311	36.311	LLKEGEEPT	LLKEGEEPT
Q5XIS7	146	146	S	TPTRVSSS	36.585	36.585	VLS(0.992)	VLS(21.21)
Q9JIR0	149	149	S	HCTILKEEI	26.673	26.673	KS(1)S(1)F	KS(26.67)S
Q9JIR0	148	148	S	QHCTILKE	26.673	26.673	KS(1)S(1)F	KS(26.67)S
Q9JIR0	153	153	T	LKEENQM	26.673	26.673	KS(1)S(1)F	KS(26.67)S
P12839	767	767	S	GQEEEKG	218.2	127.97	GVVTNGL	GVVT(-71
P12839	551	551	S	KSDQAEE	110.13	110.13	EGS(0.5)S(	EGS(0)S(0)
P12839	550	550	S	VKSDQAE	169.98	169.98	EGS(0.543	EGS(0.75)S
P15205	1942	1942	T	EDGGYSCI	155.1	155.1	T(1)PEEG(	T(49.93)PE
P15205	1789	1789	Y	KSDISPLTI	108.25	108.25	ES(0.238)S	ES(-4.84)S
Q80X08	744	744	S	SGVKPVDI	38.374	38.374	AENAAAS	AENAAAS
P15205	1786	1786	S	LSPKSDISI	126.29	63.69	ES(0.135)S	ES(-8.56)S
P15205	1380	1380	S	NERSSISPI	91.767	91.767	S(0.022)S(	S(-17)S(-1
Q9JJ19	275	275	S	EIQKENSR	35.748	35.748	EALVEPAS	EALVEPAS
Q6P3V7	1180	1180	T	SDKAKYFF	21.775	20.427	T(0.75)LT(	T(0)LT(0)P
Q6P3V7	1178	1178	T	LVSDKAKY	21.775	20.427	T(0.75)LT(	T(0)LT(0)P
P34926	980	980	S	PPGEPALC	148.19	36.284	CLS(0.994)	CLS(24.07)
Q9JK11	295	295	S	FSELEYSEI	84.942	38.017	GS(0.818)I	GS(6.56)PI
B5DF21	472	472	S	SGYRQQR	33.835	33.835	GFGQS(0.2	GFGQS(-5
Q5M876	193	193	S	ELGPQPQ	29.154	29.154	NGIS(0.17	NGIS(-6.7
P15205	1201	1201	S	DATDGRD	138.92	138.92	DYNASAS	DY(-79.45
P34926	1375	1375	T	GALDEENI	75.479	75.479	QQDKT(1)	QQDKT(75
P34926	1160	1160	S	SVVSPDTT	77.746	77.746	QEAT(0.06	QEAT(-11
P34926	1447	1447	S	TQATEPRI	118.4	52.693	RDS(1)EEK	RDS(52.69
P34926	984	984	S	PALGEVEE	115.78	115.78	CLS(0.013)	CLS(-18.0
P12839	667	667	S	KEEKPKDV	115.14	32.946	KAES(1)PV	KAES(32.9
P15205	1778	1778	S	VQSLEGEK	110.53	48.44	S(0.017)DI	S(-17.55)D
P15205	1772	1772	S	SLASEKVQ	76.85	76.85	VQSLEGEK	VQS(-42.4
P12839	643	643	S	KEEEKVEE	64.675	40.725	KEVAKES(	KEVAKES(
Q9JK11	487	487	T	EKKIEERK/	77.527	77.527	AQIIT(0.0(	AQIIT(-23)
P15146	449	449	S	TETEPQTK	78.098	69.152	LEET(0.03	LEET(-14.
Q5BK82	91	91	T	TFCPECKN	17.281	17.281	MLCQY(1)	MLCQY(17
Q5BK82	87	87	Y	QAKETFCI	17.281	17.281	MLCQY(1)	MLCQY(17
P16884	436	436	S	GLPKIPSM	60.598	44.188	VKS(1)EEK	VKS(44.19)

Position.in	Charge	Mass.error	Reporter.i	Reporter.i	Reporter.i	Reporter.i	Reporter.i	Reporter.i
6	3	-0.39761	1411	1910.9	2220.9	3056.8	4851.9	2453.8
7	3	-1.5729	2043	1948.86	3098.7	4007.4	6330.6	2607.3
1	2	0.33459	5213.7	3807.3	8298.3	15924.3	6914.7	10506.6
5	2	-3.8457	7760.8	7546.1	9825.8	9611.5	25417	10594
4	2	-3.8457	7760.8	7546.1	9825.8	9611.5	25417	10594
2	2	-3.8457	7760.8	7546.1	9825.8	9611.5	25417	10594
1	2	-0.03166	13472.7	12333	13754.7	38274	17080.8	16168.5
3	2	0.019986	11525.1	12802.5	13596.9	27188.4	19031.1	22713.3
3	3	0.33591	5849.4	4179.3	5548.2	10934.7	9930.6	5883.9
8	2	-0.47399	10230	10001	12697	13029	29509	12061
16	2	2.281	4160.3	3358.7	3119.1	3860.6	9686.4	4084.5
6	2	3.4383	2298.8	2431.5	2782.5	2973.3	6301.7	3104.1
7	2	3.4383	2298.8	2431.5	2782.5	2973.3	6301.7	3104.1
3	3	0.28928	18282.6	24792	19537.2	42426	34353	29661.3
8	2	3.715	768.69	852.09	1058.7	810.03	2268.3	1164.6
6	2	0.54819	10482.6	9099	16960.2	14484	17168.1	29909.1
6	2	-1.1116	15549	14576.4	23191.5	17237.1	32727	36780
10	4	-2.2377	4521.2	4561.8	6378.9	5232.1	12225	5277.2
4	4	-2.2377	4521.2	4561.8	6378.9	5232.1	12225	5277.2
13	4	-2.2377	4521.2	4561.8	6378.9	5232.1	12225	5277.2
9	4	-2.2377	4521.2	4561.8	6378.9	5232.1	12225	5277.2
3	3	-0.19045	2617.26	2238.42	4450.2	4445.1	5187	4374.6
2	3	-0.07304	3007.5	3688.5	3451.5	4766.7	6278.7	4223.4
3	2	1.1701	8041.5	7908	10341.6	9453.6	13260.6	18698.4
7	3	0.52646	5538.3	6507.3	7771.5	8126.7	11943.6	9710.4
3	2	-0.53626	20734.6	19400.4	27908.6	22669	50641	25394.2
12	3	-1.0159	3883.5	3455.4	5566.8	4658.1	9192.9	4622.7
1	3	-0.30905	61752	57978	76863	57129	148563	78444
8	2	0.5547	2681.22	3477.6	4238.7	3384	7256.4	4478.1
10	3	0.051848	992.31	1054.53	1130.04	1626.54	1304.13	1962.3
1	2	-0.18566	9670.2	12469.8	13738.2	19375.5	16944.9	17059.5
3	2	-1.1886	10149.3	9913.8	15792.3	12511.2	14231.1	29671.2
15	3	-0.34183	18707.1	22246.2	28248.3	31611	34926	35811
9	4	-4.4522	2440.5	2213.7	3134.2	2324.8	5988.4	2573.2
29	4	-4.4522	2440.5	2213.7	3134.2	2324.8	5988.4	2573.2
2	3	-0.15056	6451.5	7911.6	8313.9	11940.3	8854.2	12879.6
7	2	0.065884	12510	10640	14268	12215	26183	13604
9	2	-2.0074	12510	10640	14268	12215	26183	13604
1	2	-2.0074	12510	10640	14268	12215	26183	13604
1	3	1.8575	14212	12518	16474	13480	30703	15600
3	2	0.13206	4864.5	6461.1	7517.4	7088.7	8580	12002.1
11	3	-0.19098	3144.9	3983.1	4703.4	4582.5	7543.8	4106.7
2	3	-0.20122	46122	36807	45684	42060	81003	53901
1	3	0.094715	10257.3	11018.7	14603.7	14108.7	17850.6	18371.4
4	3	-0.20344	45603	46923	66774	43407	105417	66183
3	4	2.7011	3801.7	3513.2	5669	3676.6	4827.8	10782
8	3	-0.03303	10422.6	11088.9	13205.4	14864.1	14109.9	20609.1
7	2	0.21749	35502	37470	48126	66951	40830	63216
2	3	-0.74426	9683.7	9766.5	13302.9	12927.3	18034.5	13432.2
1	2	0.25747	17186.4	19257.6	21610.8	27368.4	31116	19437.6
2	2	-1.1319	4081.2	4418.1	8070.3	7736.7	6054.3	9343.5
8	3	-0.11174	5244.6	5534.7	6538.2	7629	9540.6	5967.9
2	2	-0.61432	14764	12765	13383	13563	27802	12550
4	3	0.090217	9629.1	9404.7	13636.8	14811.9	15091.2	14470.2
3	3	0.24373	7997.7	10163.4	13958.1	9198.3	20653.8	12526.2
2	3	0.24373	7997.7	10163.4	13958.1	9198.3	20653.8	12526.2
3	3	-0.35947	15830.7	15500.7	21978.9	17048.4	25828.5	31377

8	3	-0.19007	132453	119946	173853	190314	167667	233055
1	2	0.10813	8745.9	9661.5	11975.4	17259	8944.2	16030.8
1	2	0.13545	6602.4	7311.6	13885.2	8626.2	12951.3	16393.8
7	3	-0.58299	7215	8108.4	7212	11225.7	8184.6	11967.3
9	3	-0.26708	1144.71	2153.97	2096.76	2191.5	2786.16	2166.03
11	2	-0.42816	7616.1	7785.9	9618	8366.7	13258.5	12040.8
3	4	0.30818	3679.5	3655.2	4971.3	4767.6	6424.8	5103.9
3	2	0.22182	14318.7	13545	15677.7	19659.6	20398.5	18191.4
2	2	-1.2764	705.09	405.21	458.27	773.1	715.83	621.42
8	4	0.49421	93063	90063	126789	103800	156117	156237
7	2	-0.15205	12716.7	12087.9	22442.4	16905.6	20770.2	25985.1
3	3	0.27525	1499.1	1844.2	1726.2	1813.4	2762.6	2149.5
6	2	-1.206	1499.1	1844.2	1726.2	1813.4	2762.6	2149.5
3	2	0.28681	11187	8151.6	12765.3	10307.4	15265.2	18105.9
2	2	0.22521	10688.7	9680.1	12455.7	13247.7	15124.5	15658.2
5	2	0.024494	5306.1	6314.1	7223.7	9004.8	7051.2	9441.3
1	3	-1.1806	103498	89446	106156	104986	168814	118438
1	3	-0.56084	4825.8	6810.6	6181.2	9143.4	5680.8	9666.6
7	3	-0.0466	3138.3	2789.52	5532.3	3636.9	4996.2	6845.4
11	2	-0.89873	28311.3	31323	52311	34938	63702	45366
3	2	-0.02186	7630.2	8313	9053.4	10401.6	10751.4	12432
7	3	-0.1548	1081.8	1176.33	1644.21	1918.05	1227.63	2148.24
2	3	-0.75215	3140.4	2782.8	2887.17	4399.2	4238.1	2675.64
3	3	-0.53386	4357.5	3363.9	3912.3	3948	4197.3	8095.8
3	2	0.29765	17700.9	18429.9	29359.8	28658.7	25459.2	32142
3	2	-1.4745	53487	59235	72096	69456	75438	102963
7	2	-0.10105	126609	122874	163482	190131	155130	200226
3	2	0.33907	12672.9	14143.5	23586.6	26209.5	14985.6	25281.9
2	3	-0.14201	1942.71	1022.85	1767.24	2030.85	1760.22	2552.31
22	4	1.496	6258.6	5707.2	6937.8	6055.2	11621.7	6054.9
9	3	1.2758	9456.9	6482.1	11931.3	9413.7	14852.7	11121.9
6	2	0.1165	13051.8	20203.5	20939.4	19789.2	23420.1	27990.6
10	3	3.3224	313.5	378.69	483.44	487.71	377.14	715.33
8	3	3.3224	313.5	378.69	483.44	487.71	377.14	715.33
2	3	-0.32574	1857.42	2140.59	1825.68	1794.3	1964.22	4362.9
5	2	-0.01814	13390.5	13653.6	26209.8	16024.2	23590.8	29631.9
1	3	-0.29524	1738.62	1604.25	1462.68	1578.18	2333.67	2408.16
9	3	0.42414	36222	36090	48405	40890	62802	49440
2	3	-0.32859	2233.65	2318.34	2633.67	1995.39	4152.9	2964.57
3	2	0.71495	15678.3	8478	20640	14879.1	16573.2	28043.4
7	2	-0.03826	61788	63591	82113	57138	124023	77034
4	3	-0.22857	7005.6	6988.5	9172.2	7926.3	11618.1	9769.5
7	3	-0.70521	1243.59	1447.23	1876.62	1325.16	2273.07	2230.38
10	3	0.64959	7316.7	7651.5	14257.8	8185.8	12921.6	16558.5
4	2	-0.13526	15852.3	17222.4	16933.8	24078.3	19639.2	20027.7
3	4	1.0348	8212.6	8466	9054.2	8373.8	14037	9768.7
3	2	0.009357	11597.4	9243	17511.6	13582.8	17007.9	17981.4
6	4	0.236	10204.2	8815.2	11301.3	13488.6	10736.4	15138
4	4	-0.15256	2135.37	1977.75	2765.04	2552.46	3339.6	2697.45
3	3	0.30268	6648.3	7069.8	12150.9	11695.8	9214.2	11850.3
1	3	-0.41198	3552.9	2596.89	6131.4	5024.1	5330.1	4830.9
3	3	-0.04639	1471710	1389870	1727790	1715310	2086320	2009970
5	3	-0.52153	18564.6	19874.1	24086.7	23635.8	30234	24131.7
4	3	0.049196	4590	4149.3	7227	4686	7065.9	8666.7
3	2	-0.0931	21446.1	19629.9	25224.9	33117	25149	24670.5
4	3	0.22968	4884.6	4741.5	5492.7	7344.6	5332.5	6511.2
12	4	-2.5012	532.76	477.14	598.44	577.56	713.61	745.41
8	4	-2.5012	532.76	477.14	598.44	577.56	713.61	745.41

3	4	-2.5012	532.76	477.14	598.44	577.56	713.61	745.41
4	2	0.39991	13793.1	11425.2	15666.9	17642.4	16749	16829.7
8	4	0.07661	37032	34329	47943	45540	52455	51135
4	3	0.71485	16535	16286	21841	17549	28679	20790
6	3	0.71485	16535	16286	21841	17549	28679	20790
6	3	0.29092	28264.2	23548.8	31575	31926	38241	33312
3	3	0.64414	10882.5	11899.8	15064.2	18045	12759.3	16956.9
2	2	0.35765	41001	37257	45711	37068	67680	47289
3	5	-0.44401	13133.7	13094.7	14254.2	12111	22720.8	14595
3	3	0.61732	33132	28683	47982	44223	39378	55251
3	3	0.6827	1481.07	1746.06	1746.96	2137.8	1836.6	2331.24
5	2	1.8853	56976	52005	64071	74382	70173	71013
7	3	-0.24627	6460.8	6588.6	7270.8	10981.2	5796.3	8966.7
3	2	0.61617	8378.4	6277.8	8432.1	8413.5	6778.2	15377.4
5	3	-0.90963	3981.6	3937.8	5316.3	4826.4	4755.6	7317.6
3	2	1.716	40233	33648	61587	42522	61851	62646
15	3	0.24077	11928	12238.8	14004.3	15236.1	15294.9	17100.9
7	2	0.2266	39267	38199	60381	38352	48123	92757
6	3	-0.37135	18660	18321.6	22614.9	22239	26911.2	24050.7
6	3	-0.66055	3960	4433.4	4713.9	6362.1	4153.2	5981.1
5	2	1.5563	2586.3	2775.72	2279.64	3443.4	3154.8	2843.94
6	2	0.16458	8503.8	8899.2	6132	10032	9031.8	10709.4
8	2	0.19121	1377.48	1561.62	2386.26	1585.68	2094.57	3036.3
1	3	0.2126	33915	30429	37554	30090	54585	38547
3	2	-0.20687	24176.4	24804.3	32967	30879	32478	38334
4	2	-0.61635	16617	14550.9	25737.9	14976.3	23523	33252
4	2	0.25602	8918.7	12597	12710.1	13668	12180.3	17150.4
5	3	0.29912	25620	23001.9	31212	24229.2	40917	31332
3	2	0.18167	8016.6	8251.8	10959.3	10132.5	10221.6	13656.3
8	3	-0.24537	19008.3	19414.5	22448.1	19456.2	21932.7	36648
8	3	0.36997	4563	3622.2	6096.3	4324.5	6401.4	6858.6
2	3	-0.08153	28696.5	30720	34470	46281	29309.4	41010
3	3	-0.31169	31458	32808	40458	51006	34152	43929
5	4	-0.15641	30726	24236.7	30816	36483	33393	35631
10	3	-1.6359	1568.9	1125.3	1710.1	1677.6	1691.7	2098.1
3	3	-1.6359	1568.9	1125.3	1710.1	1677.6	1691.7	2098.1
3	4	0.98025	15046.8	16378.5	19598.1	17377.2	25319.4	18268.2
4	3	0.50366	10731.3	9895.2	14825.4	11407.8	16025.7	15695.7
3	2	-0.15602	6330	5960.7	7688.7	10481.4	5100.3	9399
8	3	0.047789	26785.6	27921.6	35322.2	30382.2	40585	38340.2
5	3	-0.1127	3815.4	4109.1	3985.8	4090.2	4989.6	5681.1
7	3	-0.35497	7338.3	6776.4	8317.2	9587.1	7941.9	10114.8
4	3	-0.92407	4059.6	3188.4	5766.6	3204	5528.4	7443.3
1	4	0.14279	2178.99	1955.25	3006	2376.63	2790.63	3626.7
5	4	0.14279	2178.99	1955.25	3006	2376.63	2790.63	3626.7
2	3	0.1477	3747	6012	4470.6	5183.4	6204.3	5953.8
3	3	0.10845	55494	54048	70452	77139	63258	79617
4	3	0.22359	5933.1	6454.2	10847.4	6921.6	9927.9	11319.6
5	3	1.1248	13481.1	13546.2	14420.1	16110.6	15666.3	19179.6
6	2	-0.0836	8307.3	9636.3	8241.9	11893.2	10459.8	9003.9
6	2	0.037157	8781.6	9244.5	14526	9509.7	12751.2	17687.1
3	2	-2.0181	5933.7	6698.1	9144.6	9069.3	6600	11178.3
3	3	-0.02551	33003	32859	39612	31572	51630	42489
2	4	-0.06773	20278.5	21827.1	25567.5	24299.7	21933.3	38382
5	3	-0.1638	5227.5	5402.7	5408.1	6369	5592.6	7813.5
5	2	1.3959	215464	197271	285658	193917	339828	296136
12	3	-2.0186	3747	3705.2	4023	4686.1	4475.7	4624.9
16	3	-2.0186	3747	3705.2	4023	4686.1	4475.7	4624.9

18	3	-2.0186	3747	3705.2	4023	4686.1	4475.7	4624.9
3	3	-0.04605	36096	41838	51189	55665	43722	56493
7	3	-0.53482	8895.9	8108.7	9587.7	9857.4	10683	11535.3
3	3	-0.00036	1591.8	1607.4	2545.62	1783.74	2417.07	2687.58
7	2	-0.29152	8085.3	8540.4	9490.5	6153.9	11016	15236.7
3	2	-0.10661	7045.5	7587.3	10183.2	8917.8	9339	11657.4
21	3	0.23727	3729.6	2584.89	5102.4	3859.8	4059.3	5970.6
12	3	0.15948	23659.5	22406.1	28957.2	26592	29405.1	34350
5	2	0.56866	9609.9	9774	16955.7	14168.4	11499.9	18325.8
3	2	-0.63465	7799.4	8104.5	10780.5	8337.9	11141.1	12562.5
7	3	0.19025	1894.47	1708.38	2542.41	2747.52	2238.96	2244.96
6	2	-0.04862	29067	25747.8	28703.8	24288.6	40159.4	34905.8
3	3	0.12589	8390.7	9826.8	12273.3	13566.9	9450	13730.1
5	2	1.4628	5169	6168.3	9025.5	9320.4	5115.3	10410.6
5	4	-0.00133	36273	38853	49203	52716	45009	49866
5	3	0.41459	9219	10009.8	11029.8	10698.6	10661.1	15600.6
4	3	-0.10064	25778.4	30471	34437	40827	27522	41142
3	2	-0.19825	4939.5	5505	7314.9	5682.9	7644.6	7665.9
4	2	0.078617	64341	63513	80475	79935	76338	93174
1	2	-0.37434	7550.7	11083.2	14884.5	13569.6	9498.6	17682.3
6	3	0.004641	4293	5392.5	3774.3	5558.4	5397.6	5055.3
7	3	-0.12656	10348.8	11398.2	14142.6	15945.6	10752.6	16532.4
6	3	1.256	25318.5	22326.6	23363.1	32025	24205.2	28713.3
5	2	0.34667	15389.1	14881.2	14573.7	15065.1	19892.1	18199.2
3	2	-1.2416	31887	43938	35241	50241	36591	46422
1	2	-0.61434	11922	13716.6	15519.6	16455	13292.1	20002.8
3	2	0.42873	5032.3	6421.5	7509.2	7800.7	6513.2	8238.8
2	3	0.32499	26115.9	28877.1	29866.2	32205	31050	38550
3	2	-0.32369	1808.22	2692.53	2742.21	2038.95	2876.55	3858.6
6	3	-0.32225	8850.3	7214.7	11108.1	11559.9	8772	12045.9
4	3	0.23989	31905	33201	46455	43929	39441	48729
5	2	0.56692	17184.9	15535.8	17051.1	24446.4	16302.9	17860.2
2	3	-0.14793	7440	8235.3	9519.3	7452	11330.4	10878.6
7	3	-1.4408	2437.59	2046.48	3233.7	2687.04	2980.5	3423.6
3	2	-0.2448	10438.5	12642.6	12577.2	13777.2	12257.4	16679.1
4	3	0.10721	14046	13939.2	14495.1	18962.1	14328	16906.8
2	3	0.066797	15282.3	11286.3	17832.9	16085.7	18717	16549.8
3	2	0.43209	46734	59985	63438	70011	55842	76458
10	2	1.1696	2881.38	2439.6	2011.98	2916.12	2559.63	3375.3
11	3	-0.15476	5580.6	4548.6	6308.7	6316.8	5542.5	7730.4
9	3	0.27406	13490.4	14497.5	18022.2	19872.3	14666.1	19727.1
2	3	-0.3012	18932.1	17166.6	21725.1	24465	19459.8	24150.3
5	3	0.025875	26616.6	27503.1	33144	32247	33132	36891
3	2	-0.05553	12069	13935	14106.9	16608.6	12962.4	18074.1
5	2	-0.31217	9828.3	9826.2	16896.3	12778.8	12710.1	17417.1
3	3	-0.13736	3108.9	3404.1	3374.4	4396.8	3024.3	4298.4
3	3	-0.02708	5806.2	6357.9	7623.9	7591.8	6374.1	9566.7
12	3	0.047789	7279.7	7578	8466.2	6943.9	9913.1	10491
4	3	0.56276	10245.9	7582.5	11025	9851.1	10651.5	13570.8
3	3	0.41857	8021.7	10317.6	9952.8	12363.3	7782.3	13749
3	2	0.24899	6638.1	7083	8995.8	9005.1	7314.9	10395.3
3	3	0.10401	5279.4	5760	7412.4	7289.1	5412.3	9240.9
4	2	-0.73503	11909.7	15511.2	17399.1	19698.3	13491	19305.9
2	3	0.50414	21939.3	23448.3	39885	29008.5	24216.3	48747
3	3	-0.48435	3111.9	2784.54	2379.21	3392.7	2537.13	3968.4
7	2	0.94953	17566.8	15626.4	15888	17498.7	20055.3	19132.2
2	3	0.42619	38754	43746	54783	59634	38169	63198
2	4	-0.08154	11470.8	9799.5	14536.2	11949	12756.3	17116.5

3	3	-0.29354	58601	73697	80851	88089	57791	106958
7	3	-0.5097	20425.5	22616.7	27488.7	24392.7	27219.6	29491.8
4	3	0.31145	3723.9	3754.5	5419.8	4435.5	4482.3	6053.4
3	3	0.085196	11669.4	14104.8	13542	18145.5	11448.3	16036.2
3	2	-0.27935	46080	47265	67746	58812	46437	84954
2	3	0.43625	6300.3	5555.7	6033	5764.2	6458.1	8853.9
9	4	0.61585	29284.8	29511	42291	40458	30246	46821
3	2	-2.9972	12618.9	15937.5	18518.7	17339.4	14210.7	23828.7
13	3	-0.1894	7767.9	8657.7	8427.6	10839.9	7660.8	10310.4
5	3	-0.04186	5752.2	4684.2	5322.6	5910.9	6085.8	6012.9
3	2	-0.55831	17755.2	18293.7	28393.8	23255.1	18997.5	33165
7	2	0.32917	7764.3	6652.5	7695.3	6972	8721.9	9886.2
2	2	-1.4898	14564.1	15628.8	23263.2	20318.1	14684.7	27752.4
4	3	-1.5889	18841.2	26428.2	26324.4	31047	19639.2	32916
9	3	-1.4998	2689.74	3066.9	4411.2	3396.6	3830.4	4349.1
3	2	-0.32155	8850	9274.2	15363.6	9956.1	10897.2	18380.4
3	2	0.15734	5235.3	5460.9	5946.9	5114.1	5915.7	8517
4	3	-0.74365	13806	16800	20946	21672.3	13348.8	25208.7
9	2	0.05177	10603.2	9936.9	12996.9	8964.6	12898.8	17180.4
6	3	0.010788	44601	57579	65061	64980	47322	83376
5	3	0.095946	6216.6	6040.8	6257.7	7750.8	6484.2	6811.8
4	2	1.0611	49107	58734	56892	60189	57978	71577
3	3	0.36549	29274.9	33429	40050	33396	37194	47619
3	2	0.50907	14961.9	11925.9	13584.9	14218.5	15421.2	16685.1
5	2	-0.47436	36543	26825.4	36303	33804	37050	43413
3	3	0.30387	38784	44559	56124	43446	55131	59622
5	3	0.11055	45201	41532	57654	54816	47181	63459
3	2	-0.16068	29311.8	27021.3	30084	27050.7	31419	41739
4	3	-0.08991	13651.2	14737.8	17723.1	15719.7	16034.4	21222.9
5	3	1.0312	17023.8	19297.8	21632.7	18653.4	22382.7	24856.8
6	3	1.5149	15167.4	17361.6	20598.6	17415.9	18359.7	25402.2
6	2	3.0356	15675	14579.1	19167.3	17970.9	14961.9	24431.1
6	4	-0.34879	18700.8	16372.5	18997.8	18407.7	18856.5	25116.6
1	2	0.26009	155577	149481	180048	159933	199956	183630
7	2	0.6592	12162.5	11401.7	14594.8	13629.8	13801	15808.9
10	4	0.2898	55908	46968	80505	75609	57366	73293
3	3	0.12909	3785.1	3947.4	4083.3	4278.3	3308.7	6324
1	3	0.49911	12440.7	14764.5	18655.5	14514.6	16087.2	21881.4
3	3	-0.03159	8518.8	7409.1	8252.4	8381.4	8916.6	10238.4
5	2	-0.7144	11516.7	9003	14751.9	11559.9	12105	16622.1
3	2	-0.30339	200865	207036	216879	215802	240201	249036
5	3	-0.88748	13323.1	13574.3	13615.2	13022.9	14928.7	18484
7	3	-0.67913	3237.9	3051.9	5084.4	4382.4	3425.4	5078.7
4	3	-0.28354	9957.6	9292.5	11254.2	10787.4	9906.3	14313.3
10	4	0.34676	9085.2	10017	11716.8	11412.9	10420.8	13042.8
3	2	0.030025	8677.8	9536.4	9440.4	10486.2	9553.2	11241.9
3	2	0.79816	9788.4	10721.1	12956.4	10794	12829.5	13919.7
3	2	0.48242	5081.4	4146.9	5429.1	5897.7	4818.9	5752.8
7	2	0.078772	9079.5	10208.1	11936.1	10764.9	10705.8	13902.9
3	3	0.15813	30477	33228	34602	36312	33162	41796
7	3	0.19823	72324	68025	81897	81717	81333	84618
6	3	0.067898	11504.7	10561.5	11910.9	15100.8	10079.7	13031.7
5	2	0.40261	13431.9	14135.7	18868.8	15191.7	15171	22485.3
9	2	-0.00148	18819.6	21071.4	25207.8	27304.2	18183.3	28189.8
4	3	0.13331	15795.9	16927.2	22372.5	22041	16585.8	23301.6
1	2	0.018592	13293.6	14700.6	15954.9	16911.3	11869.2	21914.7
3	3	0.20497	6046.8	6758.4	6329.7	5989.8	7025.4	8718.9
1	2	0.70387	51540	54717	69855	56355	63612	77757

9	2	-0.03834	7821	7829.1	8474.7	8515.8	6469.5	13168.8
1	4	-0.32265	2785.74	3394.8	3510.3	3076.8	2694.45	5581.2
3	4	-0.32265	2785.74	3394.8	3510.3	3076.8	2694.45	5581.2
3	2	-0.09407	51594	55728	50391	48834	55026	77202
4	2	0.38625	5481.6	4918.5	5789.7	5987.4	5151.9	7229.4
9	3	0.034469	14591.4	16076.7	17332.8	15714.9	16853.7	21718.8
3	3	0.002939	27744.9	28711.5	38826	32250	30540	45021
7	2	-0.05242	12302.4	12118.8	15372.9	15243.3	11245.8	18818.4
5	2	0.009694	14465.1	14817.3	18228.3	16911	15975	20366.7
10	2	0.049476	9716.1	10629.3	12382.2	13805.4	9206.1	13821.6
3	4	-0.06477	32967	34800	34743	45615	27800.1	42474
2	2	-0.0906	9056.7	9723	10171.8	10243.5	9584.1	12846.9
4	3	-0.0292	3792.6	3997.2	5465.4	5419.8	3757.5	5663.1
11	3	-0.49719	6252.6	4825.5	6616.8	5757.6	6148.5	8018.7
3	2	0.18895	9978.3	9209.4	12531.3	10469.1	10581.3	14654.1
2	2	3.4358	12084.6	15118.5	15351.6	17786.4	12332.4	17606.7
8	3	0.042824	33891	36843	38376	45141	33465	43200
7	3	0.40153	31987.6	40268	41641	41401	35170	52059
1	2	0.2128	21278.7	18070.2	21565.2	20087.4	21044.7	27472.5
6	3	-0.00661	94890	91509	97563	100053	103044	112047
3	3	0.15812	14316.9	13231.5	17645.1	14182.2	15462.9	21147.6
5	3	-0.13485	29742.9	26768.4	33636	29416.5	30567	41397
9	3	0.48999	15907.8	18443.1	22092.9	20026.2	16996.8	26676.9
5	3	0.018092	3326.4	3273.6	3633	3204.3	3863.4	4324.8
5	4	-0.10653	16722.3	15069.9	18951.9	15263.7	16698.9	25735.8
6	2	-0.11857	6880.8	7970.1	8467.2	9165.3	6309.6	10976.4
1	2	2.5041	12147.9	15714.9	15480.3	17729.7	11820.6	19269.9
1	3	0.06256	40731	41186	49121	38350	49794	57035
3	3	-0.18616	40731	41186	49121	38350	49794	57035
3	2	0.60365	43845	46974	56340	57603	43734	61890
8	3	0.15481	7975.2	7044.9	8587.2	8505.3	6843.3	11249.4
1	2	-0.25941	9273.6	8932.8	12899.7	10117.8	10535.4	13663.2
5	2	0.32385	9501.9	8652.6	10359.6	9366	9621.9	12684
1	2	0.89807	1016310	1063620	1183970	1110430	1218900	1218750
1	3	0.55807	21841.5	25271.7	27488.7	25461.6	20638.8	38376
3	2	-0.07179	5658.6	5584.2	7403.7	6436.8	6496.8	7367.7
13	2	-1.4952	46278	56115	68673	55707	55806	77121
4	2	-0.11904	12054.3	12070.5	12144	13861.5	10761.9	15536.7
9	3	-0.07387	56776	57441	72164	65842	62071	74466
3	3	0.14385	10586.4	11917.5	15766.2	11784.3	12202.8	18173.1
6	3	-1.6548	5303.4	6009	6636.6	6195.9	5854.2	7568.4
7	3	1.5196	6270.9	5673.6	7083.6	6327.3	6033.6	8599.2
8	2	0.50691	6408	6788.1	7327.2	6453	5926.8	10661.4
3	3	0.075196	5760.9	6005.1	7059	6998.4	5230.2	8538.9
5	2	0.052001	14370.9	16237.5	18635.1	17601.6	14806.8	21549.3
5	2	-0.06577	14453.4	13101	15260.1	16485	12424.8	17988.3
5	3	-0.13107	10148.1	10063.8	13431.6	11639.4	11019.3	13711.8
3	3	0.013014	27194.7	36330	41544	36177	30024	49509
4	2	-0.28887	26042.1	20483.1	26238	22981.2	23949.3	32940
6	2	0.11459	9645.9	9968.4	11032.2	11352.9	8396.7	14067
5	2	-0.16368	180810	153567	210018	162225	189816	239511
8	3	-0.67362	20703.6	23447.4	29352.9	21379.8	23442	35955
2	2	0.28608	16151.4	14442.3	16842.6	17779.5	14792.1	18643.2
12	3	0.35796	9847.5	11275.8	13826.1	10909.5	10932.9	16261.2
8	2	-0.03202	69138	64725	77085	66885	71919	88878
3	2	0.76775	18755.7	19078.8	18979.2	20463.6	16538.4	24911.1
4	3	0.20703	11454.3	10151.7	11237.7	11763.3	9860.4	14004.6
8	3	-0.30933	7844.7	9052.5	11376.9	8958	8888.7	12658.8

7	4	0.1362	4075.8	3822	5056.2	4424.4	3729.3	5892.9
7	3	0.22875	15796.5	13511.7	16549.8	14746.2	15080.4	19495.8
7	2	0.065891	57633	74370	82131	74841	64518	90303
5	3	-0.20478	23682.9	23254.5	27200.4	26417.4	21118.2	32367
12	3	-0.87703	9965.4	10086.9	10154.1	9440.7	9353.7	13892.7
3	3	-0.32444	6945.6	7180.2	8472.6	7411.8	6880.8	9739.2
2	2	-0.13341	21968.1	21812.4	22090.8	21927	20074.2	28180.8
3	3	0.039869	9879	9672.3	11361.3	7707.3	6006.9	10007.1
7	3	0.44579	25278.3	26270.1	30411	18065.1	17937	26644.5
4	3	-0.35043	8941.8	9712.8	10038.9	7131	6008.4	8649.3
9	4	-0.36637	82587	88197	95991	66144	49395	88788
18	3	0.27265	6778.5	6682.8	8297.1	4721.1	4683.6	7053
2	3	0.49069	30462	31836	29681.7	21584.4	18693.6	29844.6
7	3	0.30916	27580.4	29145.2	32843.8	19007.2	18351.4	30771.6
8	3	-0.2313	18106.5	20743.8	20388	13061.7	11789.4	20296.8
3	2	-0.36265	23282.1	22104	23194.8	14905.5	13401	24230.7
4	3	-0.00339	10179.9	10608.6	13538.4	7407	6965.4	11486.7
4	3	-0.66951	20887.5	25323	22999.2	15507.9	14533.2	21994.8
3	3	-0.18147	11294.4	9416.7	12439.8	7679.4	6686.7	10383
3	4	-0.11048	5565	5406.3	5787	3951.9	3155.1	5514.9
9	4	0.58896	16449.7	16181.6	18624	11768.9	9624.8	17207.4
5	3	0.12511	107658	123234	135783	79425	73956	121326
8	3	0.25499	9133.2	8191.2	10019.7	6132	6248.1	7672.8
1	3	-0.06736	407760	427580	492120	289538	240420	473020
17	3	0.11964	26975.7	29826	32955	19580.7	15890.1	32553
9	3	4.3571	1876.4	2134.5	2265.7	1377.3	1252.5	2049.9
8	3	4.3571	1876.4	2134.5	2265.7	1377.3	1252.5	2049.9
3	3	-0.1325	15703.2	16793.7	18916.2	10681.2	11440.5	15788.4
4	3	1.4301	12325.2	12372.3	14052.6	7660.2	8565	12547.8
4	3	-0.23086	5812.2	5611.8	6161.4	3960.3	3361.2	5842.2
9	3	-0.20607	7407	8552.7	8665.5	5699.4	4592.1	8125.2
8	3	0.004842	7203.2	7095.8	7893.7	5327.2	4292.44	6827.9
4	3	-1.708	4117.3	4613.9	4298.8	2875.1	2637.6	4213.4
12	3	0.4658	30219	28301.7	30342	20582.4	20346.3	23748.6
16	4	0.21229	20551.5	21344.1	22185.6	15432.3	11750.4	20634.9
8	3	0.030742	7089.9	7448.4	8575.2	5901	4824	6019.8
5	3	0.27733	27323.4	30732	35889	25212.6	17371.2	25851.9
5	3	-0.09135	9097.8	9065.4	9765	7276.5	5007	8288.7
4	2	0.22991	20284	19927.1	23480.6	14163.9	11941.8	21131
4	3	0.27246	14453.1	14752.2	16313.1	10406.1	7511.1	16287.3
7	4	0.77913	5886	6961.5	8796.3	4655.1	4104.3	7190.7
4	2	0.27234	10798.5	9281.4	13321.5	8059.8	6153	10226.4
3	3	0.25169	27861.6	30204	35097	20725.8	16772.1	31656
3	3	-0.5249	5407.5	6003.6	6741.3	4002.3	3874.8	5317.5
6	3	0.50559	6154.5	5924.1	7522.8	4383	3407.1	6792.3
7	3	0.62296	4573.2	4122.6	4565.1	2624.37	2764.62	4425.3
20	4	-0.6143	1672.35	2109.63	2281.47	1342.14	1254.39	1810.62
1	3	-0.17172	50046	56319	55131	34506	33285	50838
16	3	-0.2058	8065.8	8169	9958.2	6609	4653	7815.6
11	3	-0.17265	41634	46518	51444	31668	23246.1	48975
4	3	1.291	12775.2	12780.3	15572.4	9964.8	8220.9	11454.6
6	2	0.37254	75804	77055	89748	61320	44247	70623
18	3	0.088392	16414.2	14650.5	16429.2	11103.9	8979.3	14698.8
10	4	0.25111	10214.4	9577.5	10320.9	7536.9	5041.8	9563.4
12	3	-0.05528	2316.24	2326.11	2262.72	1513.38	1533.33	1923.96
2	3	-0.18419	24383.7	25622.1	26989.8	20724.9	12857.4	22302.3
11	3	0.30916	53312.4	57935	55726.2	38803.2	31307	51606
7	3	-0.69041	13766.4	15340.8	18085.7	10892.3	7537.9	16334

2	3	0.10231	5059.5	4167.9	5549.4	2926.68	2877.84	5007
4	2	0.21568	27567	25571.4	23526.3	17944.8	13518.3	24988.8
6	3	0.34952	10283.7	9073.8	10797	6573.3	6425.7	8607.9
1	4	0.84636	357680	335720	423790	224765	192469	408820
2	2	-0.03432	21338.4	23721.9	26256.3	12882	15212.1	23714.4
7	3	-0.06127	146589	168138	189312	102462	96426	168216
1	3	-0.45572	47613	53580	52719	32811	30360	48246
3	4	-0.10606	9597.6	9886.2	11999.4	6765.3	7389.6	7875.3
7	4	1.3497	164569	170800	189999	110972	97400	173705
7	2	-0.21242	121557	121482	128262	99714	67563	96453
9	3	0.013303	533250	588810	703080	391230	281970	672810
4	2	-0.38112	16876.2	24595.5	21775.8	13918.2	11125.2	21075.3
7	4	-0.37076	543950	570910	634890	321502	347732	605640
7	2	-0.24492	319540	330330	390970	207354	199638	346820
7	3	0.23453	4409.7	4578.6	4128.9	2952.84	2607.36	3873
7	2	0.10583	534750	544310	650680	334176	324130	600720
11	3	-0.9758	28548.9	25374.6	30795	15585	17865.9	27643.5
3	3	-0.29547	18078.9	19899.3	19963.8	14286.9	10210.5	17093.7
17	3	0.63256	8520.9	9373.8	9072	5630.7	4345.8	9963.6
3	3	0.3057	2373.3	2733.5	2497.2	1602.7	1567.9	2268.2
3	3	0.7305	11525.5	16104.9	12670.9	8146.3	8910.5	11641.1
1	4	0.20698	391853.2	385214.8	441751.8	269125.6	213395	397445.2
1	3	-0.05703	576190	594550	666080	389440	320800	618680
3	3	1.5197	15094.8	14464.2	18037.5	9429.9	9313.2	15261.9
1	3	1.5197	15094.8	14464.2	18037.5	9429.9	9313.2	15261.9
9	3	-0.04212	6230.4	7641	6539.1	4521.9	4307.4	5563.2
5	4	-0.23132	81966	100368	102510	58692	52155	93870
15	3	0.15705	13465.8	17712.6	14732.4	11192.4	7296.6	14658.3
6	4	-0.56166	13881.6	10690.5	13390.2	9246.9	7859.4	9291.9
1	3	0.011791	461730	444540	508200	235368	328830	437040
11	3	-0.04622	5982	4911.6	5709.3	2841.9	3377.1	5774.1
5	3	-0.17381	21580.2	17389.2	18966	10212	13174.8	17767.5
8	3	-1.6266	10315.5	10240.8	14048.7	6380.4	6203.4	12342
7	3	-1.6266	10315.5	10240.8	14048.7	6380.4	6203.4	12342
7	3	-0.39197	5186.1	6760.5	8954.1	4143.6	4309.8	6140.1
6	2	-0.3037	9048	11121.9	12799.2	7506.6	4896.3	11393.4
7	4	0.34012	265661	273537	299840	167510	167209	261679
4	3	1.2497	21047.4	20777.4	22872.9	14141.1	10947.9	21348
6	4	-0.72942	23887.2	23656.5	22934.7	14098.5	17444.1	16853.4
7	2	-0.15564	24622.8	35154	27968.1	21022.2	14595.9	26898
3	3	-1.0659	10365.3	9619.5	10938.9	6534.3	5139.3	10562.1
4	3	0.11595	46077	45513	45057	28572.3	23686.5	45909
1	3	-0.05712	321698	331504	365854	209478	174652	345920
12	3	0.57739	46950	51282	44715	36198	23768.4	41190
7	4	0.32913	202710	219021	248316	135192	124941	214527
7	3	-1.5309	16900.5	18300.3	18384	11028.9	9440.1	17842.5
3	4	0.26418	19876.5	24686.4	22380.9	15092.1	11816.4	20418.9
7	3	0.20166	15061.5	17169.6	17873.7	11457.9	7124.1	17469.6
4	3	0.081277	6222.3	6618.6	6419.4	4045.2	3642.3	5891.1
23	4	0.388	19664.8	23706	22709.2	13939.1	12218.9	20378
15	4	-0.35471	78473	93264	99909	58931	39288	97089
10	2	-0.46027	4015.96	4670	5996.8	3000.96	2670.4	4588.2
7	4	1.0194	563958	578770	630306	341138	328690	585892
17	4	0.20869	61761	72648	70659	42429	36699	65535
7	2	0.41654	599436	621682	694782	362298	355180	635908
4	3	-0.59307	46662	44676	51837	29186.7	24976.8	47007
4	3	-0.25738	9941.4	10832.7	11931	7869.6	5317.8	9652.2
3	2	0.88539	17994	21068.7	27818.4	13414.8	10488.9	23268

7	3	-0.05648	713700	731370	723600	445500	400470	672690
7	2	-0.53116	292716	305774	355826	183393	160162	332110
4	3	0.1383	10725.9	14079.6	15560.7	7332.3	8020.2	12659.7
8	4	3.319	4290	4506.3	5863.4	3071.2	2465.5	4687.5
5	4	3.319	4290	4506.3	5863.4	3071.2	2465.5	4687.5
12	4	3.319	4290	4506.3	5863.4	3071.2	2465.5	4687.5
4	2	0.29016	47010	49977	49044	30621	25014	47175
14	3	1.0542	11607.3	14163	12005.1	8963.4	6363.6	10995.6
9	3	0.006401	10352.1	15234.6	14700.6	9454.2	5668.2	13253.4
7	4	0.20047	497030	434710	504720	279732	258356	469450
10	4	0.46509	65718	67728	56766	36510	31503	68037
1	3	0.29983	115008	123801	124800	81078	53712	122253
8	3	-0.64994	16947.9	19376.1	21868.5	12976.5	9328.5	18162
12	4	-3.1699	2714.2	2686.6	2808.1	2045.6	1601	1864.6
3	3	-0.08958	4730.4	6298.2	5797.2	3732.3	3593.4	3946.2
4	4	-0.09271	17949	18109	22494	10711	10275	19790
6	2	0.36004	95547	98001	111387	62646	54738	92445
2	3	0.037239	2916.72	2795.34	3210	1619.43	1361.4	3352.2
9	3	0.32398	3752.1	4057.8	5222.1	3077.7	2140.92	3623.4
5	4	0.087903	5371.8	7474.2	8031.6	4653.6	3208.8	6537.9
1	4	-0.37076	181667.4	188744.4	206655	103781.4	103108	195185.2
9	3	-0.18112	32397	46515	40332	21946.2	21175.2	40029
6	2	-0.09131	30843	27959.1	29323.2	21603	15363.3	22857.9
6	2	0.66129	902340	905340	914610	459810	510540	930660
3	4	0.11468	12691.2	12129.3	13788.6	7810.2	6603.6	12271.8
7	3	-0.05712	347518	359274	422000	216266	187812	380138
4	2	0.020345	7382.4	7545	7814.7	4191.6	3984.6	7650
14	4	0.84376	3695.7	4667.6	4552.3	2842.6	1934.3	4175.6
7	3	-0.06736	243370	259108	276710	153226	129427	257442
3	4	0.061484	210081	261897	245679	152295	100821	250014
16	3	0.37236	57603	74589	85728	52764	28433.7	68931
15	3	-3.8903	1934.9	2321.1	2996.1	1787.3	1405.2	1549.3
13	3	-3.8903	1934.9	2321.1	2996.1	1787.3	1405.2	1549.3
16	3	-3.8903	1934.9	2321.1	2996.1	1787.3	1405.2	1549.3
3	2	-0.01175	15473.4	16708.5	18112.5	8373.6	7677.3	19536.9
9	4	0.84636	483210	492680	588420	283134	278312	514430
1	4	0.20047	289220	303958	330960	176954	161466	296460
1	3	0.21613	219354	220455	250623	131706	119451	223059
3	3	-0.10954	7289.7	6762.3	7601.4	4496.1	3540	6840.9
7	5	-0.00171	1036200	1070430	1126590	558780	589230	1082310
5	3	0.39228	866730	847680	829410	437250	462330	864090
2	3	-0.30199	5267.4	4503.6	4468.5	3357.6	2153.28	4275.6
3	2	1.0864	433140	574020	570300	310590	250485	527700
1	3	-1.0301	1681800	1635930	1901430	992250	794520	1847550
9	3	-0.07616	9437.1	7708.8	8497.5	5092.8	4612.5	7815.9
11	3	0.5603	17845.8	21074.1	19833.3	12567.6	7853.7	20611.5
8	4	0.21128	3485.4	2972.16	2788.29	1743.84	2173.83	2202.6
11	3	0.55712	4714.2	4209.9	4896	2847.15	2918.04	3372.6
9	3	-1.1796	19243	21026	20050	12734	9122.4	19814
6	3	0.61953	10566	10700.1	11798.4	6774	6288.9	9115.2
5	4	-0.21107	252405	236544	243387	132804	121323	254091
1	4	1.0194	126137	127800	141582	74209	66906.4	130437
9	3	-0.20821	5917.2	5238.3	5506.5	3612.9	2544.27	5297.7
8	4	-0.62303	12757.2	13477.5	15470.1	9303.9	5238	14346.6
7	4	4.4683	14548.8	12990.9	14167.8	6609.6	9222	12206.1
5	2	0.55201	24258.9	27157.5	25585.2	17318.4	11892.3	23285.1
7	4	-0.00955	32406	34288.6	39293	20226.6	17144.9	35234.6
3	2	-0.17667	42651	42699	43038	21258.6	21102.9	46977

6	3	0.43142	14797.5	16047.9	16651.8	10515.6	8989.5	11872.8
2	3	0.14281	6232.2	7737.6	7967.1	4670.4	2735.22	7799.1
5	4	0.002114	5897.6	8784.2	10322	4397.7	3640.3	9188.4
7	2	-1.3083	116124	121192	143777	69582	60148	130880
1	3	0.021413	288198	307050	337470	167808	151548	318450
9	4	0.043855	23376.2	28268.4	27807.2	13741	14753.8	25145.4
10	3	-0.16813	4416.9	5090.4	4506.3	2971.71	2811.6	3426.9
9	3	-2.4305	33909	36618	31836	24259.8	14031.3	31383
7	3	-0.05703	203803	213898	248791	116985	113425	221073
4	4	0.63453	3616.2	3907.8	4191.6	2975.97	1662.27	3153.6
4	3	0.3982	96207	111513	98526	72978	34455	103740
9	3	-0.37284	3211.2	2793.39	3091.2	2059.05	1709.01	2186.22
8	3	-0.37284	3211.2	2793.39	3091.2	2059.05	1709.01	2186.22
10	3	0.33873	3108.9	2724.09	3424.5	1809.3	2205.99	1865.13
11	3	0.092981	12477.3	13123.8	13231.5	8213.1	6605.7	11031
12	3	-0.049	33654	37314	34656	21318.9	15143.4	35694
16	4	0.27041	13895	14432	15250	8173.1	7840	13045
4	2	0.21805	24091.2	27129.6	30405	16806.9	11272.8	27184.5
4	2	-0.65541	11605.2	11342.1	10492.8	8438.4	4455	9519.6
6	3	0.13295	23500.8	23376.3	33582	12693.6	13656.9	27748.8
7	3	0.14755	116167	123432	135982	61754	63384	128945
1	3	0.51964	8794.8	10556.1	12720.3	5216.4	6262.5	9634.2
15	4	0.30956	22751.1	26498.4	30975	16246.8	13745.7	22734.9
3	3	-0.10761	4185.9	3659.1	3723	1658.1	2590.41	3390
7	3	-0.40507	9395.4	7018.5	9252.3	5797.8	4759.5	5987.1
5	3	0.064008	64965	56571	56592	33600	32712	51879
9	3	-0.18876	5912.7	8235.3	8498.1	4905.9	4385.1	5175.3
4	3	0.38231	8457.3	8717.7	10578.6	5894.7	4223.7	8221.2
3	3	-0.27551	4327.8	4298.7	4479.9	2977.29	2996.67	2177.13
17	4	-0.97905	14840.1	15379.5	16924.8	8779.5	7200.3	15681.9
2	3	0.18919	4646.4	4897.2	5836.5	3784.8	1610.85	4924.8
3	3	0.16671	2637.09	3151.5	2610.39	1514.04	1531.26	2527.11
9	2	-0.52705	12801.3	10054.8	11195.4	4823.4	6870.6	10979.4
8	4	-0.18329	47516	61310	66802	43390	18751.4	55119
10	3	0.76537	7361.7	5613.3	7773.6	4003.8	2799.18	7196.1
3	3	0.012614	5131.2	5345.1	4303.2	3460.8	3153.9	2685.9
9	2	-0.85749	69573	78993	45045	46776	34107	44862
10	3	-0.60015	6875.1	4991.7	6317.4	3311.1	3015.6	5685.3
22	4	0.34786	33387	46332	42381	24445.2	18772.2	37140
5	4	0.30081	114276	93558	113004	56778	49158	107358
3	3	-0.34369	43467	45024	59928	25849.2	19717.2	53538
10	2	-1.0506	17844	16809.3	21775.2	8947.2	7598.1	21503.7
3	3	0.14685	81735	87807	99207	40635	44589	92793
12	4	1.2693	9361.2	8885.7	8197.8	5259	6290.4	4750.2
6	3	-0.18557	21784.5	21916.5	23891.1	10356.9	14564.4	18171.9
5	3	-3.0362	11866.8	12636.1	14427.7	9544	4626.1	11169
7	3	-1.0691	14937	14778	8173.2	8409.6	6066	10404
10	3	-0.99362	9965.4	10611.9	10728.9	6240.3	3985.8	10605
13	5	0.25964	418380	435390	485100	197697	241869	436950
1	4	-0.37722	126470	132130	149550	60273	70351	137120
7	2	0.29244	27943.6	33908	34681.2	23188.3	12237.9	26916
5	4	0.52	4171.8	6515.7	6840.9	3049.2	3306.3	4761
1	4	-0.25974	9751.8	10803	10491	5823.3	3593.1	11463
8	3	1.2902	32631.4	41821	41726	23718.1	15068.7	37402
7	4	0.030153	66079	66088	77742	31069	37071	68376
14	3	0.11083	2279.73	2118.6	2539.2	1216.05	1060.14	2249.61
6	4	0.37971	8667.3	10668.6	12045.3	5121.9	5297.7	9838.8
10	3	0.44724	40554	30777	20417.1	21309	13771.2	24579

4	3	-0.28158	119298	140058	163425	69774	73992	127368
7	3	-0.2673	14147.8	15317.4	15795.6	8785.8	7483.8	12699.4
3	2	0.010423	81411	86289	97464	48210	34500	92286
1	2	0.13636	21545.4	24260.1	26466.6	11203.5	13819.8	21049.2
5	3	-0.46176	120384	136593	133752	82902	44850	129264
8	3	-0.0306	5589	6033.3	6526.2	3566.42	3080.72	4847.4
1	3	0.54996	87060	82887	98073	48108	36096	91308
10	3	0.094166	10221	11960.4	10222.8	5415.9	5470.8	10074
3	3	-0.0604	5322.3	3562.5	4698.9	3566.4	2506.29	2109.33
5	2	0.086253	992.39	1054.4	958.19	671.14	562.32	618.75
4	3	0.18083	9135	10268.7	8970.9	5477.7	3575.1	9549.9
1	3	-0.05553	73360	76833	90153	41119	32527	82737
6	3	0.38245	7235.7	6651.6	12201.9	4455.6	3731.1	8369.7
6	4	-0.55883	73454	86032	82208	49844	34858.8	68549
7	3	-0.43511	6369	8124	6975.3	4090.8	3111.6	6525
10	3	-0.00797	22805	23781.4	24551.6	11175.6	12493.1	21387.2
7	4	-0.37722	206032	207026	247910	84397	118120	218322
3	3	0.14014	7066.5	7797.9	8757.9	3819.3	3131.1	8299.8
6	3	-0.5085	4212.6	3578.1	5360.7	2207.79	2007.72	4053.3
3	2	-0.05701	24069	26005.5	31047	15855.9	10443	25049.1
12	4	-0.55883	17155	17964	17011	9800.2	8883.3	13623
18	5	0.62354	468480	597480	500340	350880	174504	471780
10	2	0.029829	98466	123670	112687	68162	45202	97453
4	3	0.52986	22105	22812	25613	12841	8870	23319
5	2	-1.1672	10330.5	10821.6	10909.8	5754.3	4436.1	10181.4
3	2	0.023942	31848	34809	40575	17172.6	14881.2	36252
7	3	0.23257	4971.9	4708.8	5771.7	3283.2	2102.25	4197.9
3	2	0.056909	14336.1	14667	11495.7	8064.3	5214	12539.7
8	3	-0.28044	9547.2	11356.2	11644.5	5420.1	5740.5	8887.8
6	3	1.96E-05	42156	50013	50478	24563.1	21607.5	43242
17	5	-0.45675	659300	680540	640090	341960	293082	615570
4	4	0.29945	1426.89	1398.39	2071.86	956.52	574.38	1539.51
16	4	-3.9008	1335.8	1068.3	1315.8	684.19	454.98	1228.9
9	4	-3.9008	1335.8	1068.3	1315.8	684.19	454.98	1228.9
1	3	0.24098	634170	728370	643650	304290	299520	675450
8	4	0.13616	19508.7	20511.3	26298.3	16105.5	4839.3	21100.2
21	4	-0.04254	4871.5	5334.3	5251.5	2904.9	2827.3	3601.6
1	4	-0.29242	71274	77817	95079	44007	37746	68433
1	4	0.050675	22203	20768	24782	9074.3	11979	21057
4	3	0.50138	7758.6	8310.3	7696.2	3926.4	4056.9	6684.6
1	3	0.28433	276984	288522	337590	123984	136734	308280
12	4	0.095265	6543.3	6886.5	5889.9	4524.3	3698.4	3015.6
2	3	-1.4868	8811.3	8596.5	8388.9	3427.8	4501.8	8157.3
10	3	0.31739	110154	133908	130101	76131	46386	108444
14	3	-0.09421	10455.9	13302	12300.6	7511.4	4374.3	10353.3
18	4	0.48216	5145.3	5679	5818.2	3681.9	3067.8	2894.07
9	4	0.17095	79347	101517	87534	57042	30540	78678
1	4	-0.45435	9753.6	10766.1	7728.3	6277.8	4266	6492
5	2	0.036349	11024.7	13982.6	14460.9	7766.3	4981.9	11340
4	3	-3.5451	2079.7	2266.8	2237.8	1561.6	523.99	2009.6
6	3	-3.5451	2079.7	2266.8	2237.8	1561.6	523.99	2009.6
3	3	-3.5451	2079.7	2266.8	2237.8	1561.6	523.99	2009.6
4	3	0.31956	13230.6	12571.8	15825.6	7404.6	4911.6	13480.5
8	2	0.07088	5666.5	6468.9	5614.2	3614.6	2386.7	4788.9
3	4	0.15281	30756	36465	33321	19564.5	13278.9	28284.9
15	4	-0.10322	65933	71404	70540	36544.4	29133	61050
11	3	1.5692	4611.3	3329.4	3795	2842.38	2127.54	1688.1
3	2	-0.48785	26720.4	25638	36696	13213.5	11312.1	30393

6	2	-0.0617	115419	139569	116001	73722	40959	113643
4	2	-0.45808	47070	52170	67851	27071.7	20457.9	54258
10	3	1.151	8592	10634.1	10267.8	5180.7	4856.1	6977.7
4	3	-0.07217	176785	196076	195008	101771	75307	157726
13	4	-1.1988	11292.6	12805.5	10966.8	8430.6	3048	9338.7
8	3	-0.18175	3001.2	3424.2	2775.21	2230.65	1060.02	2041.56
10	3	-0.83306	1884.6	1975.5	1945.5	853.81	1069.5	1415.2
16	4	0.12119	30720	36405	37881	20250.3	11574.6	29948.1
4	4	0.27611	172275	216927	240126	100371	84762	182895
7	3	-1.155	1209.27	1306.8	1600.98	488.97	974.04	791.07
8	3	0.041504	16287.1	23072.9	23670.4	10190.5	6051.8	21722.7
1	4	-0.57796	223141	220269	224284	118473	66331	215708
4	3	-0.5964	119265	146055	179202	72117	53493	135165
6	4	0.27542	2712	2792.1	2700.2	1321	1245.4	2179.3
7	3	0.22551	52839	63711	63171	31983	21626.7	51315
1	3	1.2992	7117.2	7084.2	5945.4	3471	4707	2583.69
4	2	0.14261	51663	57168	58512	30384	21072	45051
1	3	-0.02735	6270	6704.4	8176.2	3400.5	2073.75	7080.6
11	4	1.4472	1086560	1113440	1098490	489350	440280	1000510
3	4	0.13393	223222	239744	224778	133452	52054	225080
7	2	0.55945	8906.7	10775.1	12813.3	6702.3	1822.68	10772.1
4	3	-0.40971	22216.5	27722.7	30399	15072.3	7065.3	24948.6
9	4	0.075866	16601.4	18026.1	20405.4	10993.5	4817.7	16263.3
3	3	0.15339	8805	10624.2	9989.4	3226.5	7416	5081.4
3	4	3.8499	3558.2	3779.1	3706.8	1941.1	1550.7	2771.5
2	4	3.8499	3558.2	3779.1	3706.8	1941.1	1550.7	2771.5
7	4	3.8499	3558.2	3779.1	3706.8	1941.1	1550.7	2771.5
10	3	0.34141	265227	315300	287778	167799	102219	226722
4	5	0.22177	12614.1	10552.8	12083.1	4388.7	4303.5	12221.7
3	4	0.087839	43173	33351	42198	16085.1	12221.1	42273
1	3	0.71317	118908	147714	149112	75222	29234.4	140748
6	3	-0.55995	15834	17956	16929	7961.6	6412.1	14545
7	3	-0.12785	2257.23	3120.9	3178.8	1152.06	1176	2531.4
3	3	-3.0362	55211	69075	65048	37761.4	17465.4	52779
13	3	0.19689	11651	13050	12862	6930.8	3801.4	10491
8	4	0.95101	13143.9	15312.9	10897.5	6295.8	4386.3	11875.5
3	4	-0.14628	1682.6	1816.2	2099.2	949.65	627.67	1534.7
1	4	-0.14628	1682.6	1816.2	2099.2	949.65	627.67	1534.7
3	3	-2.8111	54030	57882	66624	20879.1	30315	46257
2	3	0.33237	4482.3	6672.9	5491.8	2824.95	2158.26	4119.9
7	3	-0.23342	3554.7	4023.9	2396.73	2039.97	1646.61	1562.49
21	4	0.14289	14577.9	11820.9	11998.2	5481.3	6619.2	8547.6
10	4	0.021751	400570	509660	498860	272189	132165	356250
5	3	-0.0021	25026.6	29571.3	28842	13432.8	9248.4	22542.3
7	3	-0.41984	37089	41370	48216	20330.1	10317.3	39264
3	3	0.46331	91845	110604	112089	56088	24096	92814
7	2	-0.25012	21632.1	26069.7	31614	9611.1	11028	21704.7
4	4	0.053809	3151800	3500100	3113400	1729080	747960	2897520
4	2	0.067861	54160	58845	54833	29823	20705.4	37490
10	3	0.22374	296028	383580	294357	182682	93183	239442
7	4	0.053809	494250	497580	428700	216609	115641	441630
8	3	0.63498	17699.1	28531.2	27023.7	13905.9	4201.2	20815.8
8	4	0.20841	51006	50826	64530	25488.9	13673.7	48180
9	3	4.1068	3684.2	4703.3	4490.6	2485.5	860.76	2232.1
5	3	4.1068	3684.2	4703.3	4490.6	2485.5	860.76	2232.1
3	4	0.053876	85416	129366	122046	44421	22862.7	63969

baseMean	log2FoldChange	lfcSE	stat	pvalue
2787.171799	-1.160338159	0.353368351	-3.283650494	0.001024719
3530.853867	-1.149138577	0.383995741	-2.992581567	0.002766287
8558.133871	-1.145121742	0.328511481	-3.485789107	0.000490688
12484.73637	-1.130146685	0.619092946	-1.825487906	0.067927435
12484.73637	-1.130146685	0.619092946	-1.825487906	0.067927435
12484.73637	-1.130146685	0.619092946	-1.825487906	0.067927435
19204.87497	-1.067562154	0.347466271	-3.072419526	0.002123311
18161.83964	-1.043213492	0.207325031	-5.031777821	4.86E-07
7382.674211	-1.017287468	0.328508913	-3.096681486	0.001957
15401.46947	-1.000127169	0.382428978	-2.615197138	0.008917597
4991.662756	-0.976184759	0.408477916	-2.389810368	0.016857074
3475.581596	-0.971648388	0.363040984	-2.676415147	0.007441441
3475.581596	-0.971648388	0.363040984	-2.676415147	0.007441441
29106.2554	-0.965641796	0.267627927	-3.608150342	0.000308388
1203.219521	-0.913987224	0.417014209	-2.191741203	0.028398199
16079.23598	-0.891224081	0.239560668	-3.720243761	0.000199031
23409.12166	-0.877085283	0.270969179	-3.236845191	0.00120859
6662.213072	-0.827804867	0.385610313	-2.146739441	0.031814031
6662.213072	-0.827804867	0.385610313	-2.146739441	0.031814031
6662.213072	-0.827804867	0.385610313	-2.146739441	0.031814031
6662.213072	-0.827804867	0.385610313	-2.146739441	0.031814031
3954.323114	-0.817586802	0.282299197	-2.896171196	0.003777463
4381.494076	-0.80962092	0.285869169	-2.832137943	0.00462379
11226.53681	-0.800846874	0.20660586	-3.876205995	0.000106098
8447.232826	-0.797614901	0.250771269	-3.180647062	0.001469465
28901.22687	-0.794145203	0.353803395	-2.244594635	0.024794168
5430.515481	-0.779460038	0.357350465	-2.18122016	0.029167136
83185.76773	-0.77907263	0.359705606	-2.165861799	0.030321749
4382.838395	-0.777823963	0.339050698	-2.294122878	0.021783442
1347.063496	-0.772736961	0.184527814	-4.187644909	2.82E-05
15130.83134	-0.770815406	0.215444038	-3.577798723	0.0003465
14975.37022	-0.766405085	0.24359326	-3.146249146	0.001653791
28932.03976	-0.758943734	0.198276194	-3.827709812	0.000129341
3252.373857	-0.752374006	0.404242021	-1.861196925	0.062716372
3252.373857	-0.752374006	0.404242021	-1.861196925	0.062716372
9419.214352	-0.729928642	0.172950228	-4.220454925	2.44E-05
15475.78616	-0.722579193	0.345937518	-2.088756364	0.03672966
15475.78616	-0.722579193	0.345937518	-2.088756364	0.03672966
15475.78616	-0.722579193	0.345937518	-2.088756364	0.03672966
17833.92214	-0.71829376	0.353369487	-2.032698879	0.042082955
7713.493666	-0.707894737	0.198176507	-3.572041657	0.000354209
4842.221254	-0.706369473	0.330768105	-2.135542882	0.032716691
52406.67268	-0.678440429	0.30202423	-2.246311263	0.024684078
14493.29711	-0.677970907	0.202223689	-3.352579073	0.000800624
64000.34725	-0.669290016	0.33616648	-1.990948106	0.046486593
5201.187484	-0.667920782	0.286883035	-2.328198951	0.019901542
14007.51792	-0.666046127	0.133211128	-4.999928594	5.74E-07
48707.61247	-0.66456375	0.200091195	-3.321304314	0.000895978
13139.99151	-0.661160796	0.258729206	-2.55541617	0.010606091
23388.96468	-0.660071555	0.285383366	-2.312929321	0.020726525
6555.728354	-0.657307874	0.2353643	-2.792725461	0.005226603
6952.13515	-0.6529372	0.288510509	-2.263131428	0.023627589
16516.73066	-0.647408712	0.364445827	-1.776419604	0.075663782
13005.2157	-0.646453338	0.213539772	-3.027320539	0.002467322
12735.31701	-0.644932281	0.342398732	-1.883570881	0.059623044
12735.31701	-0.644932281	0.342398732	-1.883570881	0.059623044
21214.14537	-0.643959644	0.220155903	-2.925016473	0.00344438

169284.78	-0.637227424	0.154678862	-4.119680064	3.79E-05
12061.96236	-0.632868616	0.229829189	-2.753647697	0.005893517
10852.19841	-0.630099078	0.276610721	-2.277927174	0.022730917
9021.637549	-0.627127938	0.185396685	-3.382627562	0.000717959
2132.164031	-0.622994422	0.313392446	-1.987905034	0.046822194
9905.095513	-0.622174785	0.239385286	-2.599051907	0.009348164
4855.860941	-0.620894162	0.25296804	-2.454437177	0.014110532
17283.46141	-0.619407629	0.215690928	-2.871737043	0.004082225
628.9124631	-0.616219673	0.362874159	-1.69816356	0.089476896
121848.142	-0.614162507	0.223404656	-2.74910343	0.005975853
18377.51382	-0.611823925	0.231121693	-2.64719385	0.008116281
2009.485526	-0.611120034	0.283284871	-2.157263226	0.030985161
2009.485526	-0.611120034	0.283284871	-2.157263226	0.030985161
12628.44713	-0.609290066	0.232916559	-2.615915623	0.008898855
12936.14761	-0.608754565	0.188947811	-3.22181327	0.001273821
7408.725108	-0.606387227	0.174713999	-3.470742071	0.000519022
118184.6424	-0.60612712	0.273095102	-2.219472688	0.026454583
7034.292071	-0.60486697	0.216337007	-2.795947755	0.005174776
4434.995971	-0.60199007	0.263174701	-2.28741618	0.022171543
43363.44829	-0.600163474	0.306769143	-1.956401055	0.050417928
9828.657894	-0.598608224	0.16453445	-3.638193851	0.000274557
1519.370933	-0.596019052	0.228470376	-2.608736684	0.009087714
3466.950834	-0.58669126	0.313020806	-1.874288382	0.060890688
4567.549632	-0.583510415	0.223096446	-2.615507441	0.008909498
25264.10894	-0.583036166	0.196094413	-2.973242105	0.002946719
71835.76955	-0.580887205	0.150885528	-3.849853666	0.000118188
160125.4541	-0.576193317	0.162736039	-3.540662046	0.000399124
19317.68657	-0.574843421	0.247971872	-2.318179948	0.020439543
1843.022785	-0.574103331	0.266942858	-2.150660019	0.031503044
7354.572892	-0.573413578	0.338323689	-1.694866771	0.090100697
10727.90099	-0.567726746	0.292537943	-1.940694394	0.052295357
20917.65257	-0.564983467	0.211679963	-2.669045564	0.007606713
452.0075767	-0.562130763	0.279159701	-2.013652978	0.04404598
452.0075767	-0.562130763	0.279159701	-2.013652978	0.04404598
2268.530688	-0.559781029	0.265145298	-2.111223666	0.034753092
20206.05134	-0.558762841	0.264843686	-2.109783505	0.034877008
1875.147525	-0.558206802	0.259298436	-2.152758077	0.031337697
46401.13182	-0.55711132	0.252412468	-2.207146596	0.027303819
2774.415101	-0.555562224	0.316447387	-1.755622725	0.079152849
17055.02273	-0.551756762	0.278228118	-1.983109277	0.047355225
79549.28768	-0.549189897	0.319312024	-1.719916118	0.08544769
8870.895208	-0.546006134	0.242806531	-2.248729194	0.024529731
1740.895686	-0.540589253	0.278523403	-1.940911421	0.052269023
11013.33946	-0.540577236	0.274321935	-1.970594282	0.048770301
19244.88666	-0.538316525	0.214098225	-2.514343709	0.011925415
9883.974936	-0.537897073	0.28066025	-1.916541699	0.055296181
14509.75022	-0.53718401	0.237292626	-2.263804063	0.023586169
11610.27428	-0.536688774	0.160626364	-3.341224694	0.000834097
2619.435141	-0.534067635	0.258971384	-2.062265055	0.039182508
9744.433282	-0.533132097	0.227211776	-2.346410501	0.018955218
4608.795525	-0.532417631	0.295414926	-1.802270583	0.071502844
1752377.049	-0.531781447	0.197548194	-2.691907408	0.007104467
23834.41701	-0.531685949	0.236734186	-2.245919602	0.024709158
6019.538559	-0.528060952	0.249240418	-2.11868106	0.03411743
25235.32911	-0.525155249	0.231382795	-2.269638279	0.023229539
5760.205321	-0.521535673	0.20069495	-2.598648714	0.00935915
611.9823929	-0.5208064	0.267521675	-1.94678207	0.051560867
611.9823929	-0.5208064	0.267521675	-1.94678207	0.051560867

611.9823929	-0.5208064	0.267521675	-1.94678207	0.051560867
15520.11056	-0.518858766	0.205085322	-2.529965389	0.011407378
45138.79898	-0.518398758	0.201008035	-2.578995211	0.009908816
20660.01198	-0.515431145	0.271541828	-1.89816482	0.057674373
20660.01198	-0.515431145	0.271541828	-1.89816482	0.057674373
31608.42766	-0.513723659	0.223890737	-2.294528422	0.021760164
14290.9188	-0.513330126	0.191093722	-2.686274151	0.007225378
47002.44059	-0.511386989	0.284706882	-1.796187663	0.072464676
15375.37808	-0.510244204	0.300957863	-1.69540081	0.089999412
41174.19496	-0.508974781	0.182535088	-2.788366816	0.005297452
1886.941295	-0.508161192	0.191937604	-2.647533265	0.008108138
65499.88877	-0.507766774	0.192865935	-2.632744728	0.008469798
7697.252888	-0.505935945	0.237868705	-2.12695464	0.033423852
8739.677554	-0.504885832	0.217702809	-2.319151666	0.020386814
4971.534125	-0.500896465	0.15890768	-3.152122435	0.001620883
50477.69275	-0.499537542	0.246819458	-2.02389855	0.042980591
14389.17992	-0.497371576	0.167483883	-2.969668291	0.002981215
51420.33681	-0.497150582	0.241271326	-2.060545653	0.039346406
22422.17316	-0.496824723	0.213032137	-2.332158565	0.019692351
4941.580073	-0.495767567	0.20076641	-2.469375061	0.013534927
2905.527763	-0.493970931	0.259142632	-1.906173937	0.056627642
8989.233557	-0.491738142	0.236965368	-2.075147713	0.037972847
1977.731344	-0.490957927	0.248805013	-1.973263804	0.048465514
38301.66564	-0.48992822	0.283250904	-1.729661629	0.083690748
30630.17701	-0.489367596	0.165361192	-2.959386002	0.003082527
21112.57341	-0.488305388	0.255798012	-1.908949115	0.056268656
12827.02618	-0.487026649	0.17606615	-2.766157208	0.005672118
29861.96561	-0.486051424	0.266830679	-1.821572488	0.068519879
10161.92675	-0.485675758	0.154114597	-3.151393627	0.001624933
22820.71389	-0.484639937	0.172577432	-2.808246309	0.004981211
5311.474509	-0.484201036	0.244284031	-1.982123164	0.047465458
35170.77867	-0.482384981	0.199902842	-2.413097161	0.015817602
39121.95654	-0.482298049	0.201593649	-2.392426798	0.016737367
32192.38669	-0.481684331	0.198344062	-2.428529126	0.015160207
1645.873411	-0.480980337	0.220206002	-2.184229005	0.028945425
1645.873411	-0.480980337	0.220206002	-2.184229005	0.028945425
19028.15321	-0.477510995	0.26172343	-1.824486995	0.068078481
13160.74929	-0.47750914	0.225488155	-2.1176684	0.034203161
7453.061957	-0.477392576	0.243760577	-1.958448661	0.05017739
33502.14067	-0.474836275	0.211412007	-2.246023208	0.024702521
4466.422435	-0.47319435	0.193634802	-2.443746403	0.01453564
8365.422148	-0.471421003	0.166016077	-2.839610549	0.004516864
4798.746152	-0.468729788	0.279988905	-1.674101296	0.094110693
2638.840037	-0.467125314	0.20434374	-2.285978092	0.02225554
2638.840037	-0.467125314	0.20434374	-2.285978092	0.02225554
5330.868001	-0.466343691	0.256431662	-1.818588579	0.068974222
66763.44787	-0.465989263	0.165174546	-2.82119293	0.004784542
8516.763436	-0.465152504	0.248054309	-1.875204288	0.060764624
15449.88795	-0.462563891	0.154025168	-3.003170815	0.002671824
9787.098347	-0.458305784	0.252749199	-1.813282834	0.069788209
11924.9375	-0.455791433	0.222766626	-2.046049003	0.04075155
8007.063947	-0.455715845	0.181861069	-2.505846068	0.012215879
39048.63786	-0.45560519	0.249752664	-1.824225545	0.068117981
25020.17976	-0.453717085	0.145724734	-3.113521447	0.001848691
5964.371748	-0.452901999	0.153293395	-2.954478239	0.003131982
256825.1458	-0.450581178	0.258352809	-1.744053719	0.081149723
4253.177508	-0.449030171	0.19946732	-2.251146561	0.024376256
4253.177508	-0.449030171	0.19946732	-2.251146561	0.024376256

4253.177508	-0.449030171	0.19946732	-2.251146561	0.024376256
47499.48629	-0.44845982	0.174807007	-2.565456763	0.010304006
9840.595195	-0.448131195	0.180319299	-2.485209284	0.012947529
2099.569947	-0.448005863	0.244319709	-1.833686957	0.066700476
9647.772217	-0.446307741	0.256087719	-1.742792442	0.081369877
9092.460115	-0.442955768	0.169320685	-2.616075929	0.008894678
4164.741806	-0.442588887	0.232684345	-1.902099978	0.057158085
27585.50961	-0.441942954	0.164451721	-2.687372024	0.007201669
13198.42881	-0.441610995	0.208110429	-2.122003198	0.033837469
9778.151299	-0.440799028	0.200144294	-2.202406174	0.02763663
2248.679885	-0.439864449	0.246998659	-1.780837397	0.074939018
30876.86693	-0.439429046	0.249831976	-1.758898333	0.078594778
11163.56231	-0.43889442	0.188501377	-2.32833535	0.019894304
7408.211741	-0.438243842	0.241901856	-1.811659688	0.070038796
45554.28595	-0.437656996	0.187304675	-2.336604767	0.019459744
11126.61229	-0.435216812	0.138057589	-3.152429457	0.001619179
33259.95641	-0.434942063	0.182259523	-2.386388679	0.017014758
6481.023374	-0.433618763	0.219739191	-1.973333754	0.04845755
76329.25966	-0.433471177	0.151561429	-2.860036213	0.004235926
12157.65795	-0.432412494	0.22826476	-1.894346262	0.058179064
4999.786161	-0.431140986	0.258249617	-1.669473861	0.095023506
13118.15589	-0.431118947	0.181395053	-2.376685254	0.017468987
26213.92056	-0.430380559	0.202883636	-2.121317257	0.033895113
16553.8875	-0.429441253	0.223305751	-1.923108796	0.054466388
40980.31634	-0.428371057	0.21713259	-1.972854726	0.048512116
15059.07735	-0.427684933	0.146168196	-2.925978052	0.003433752
6915.933139	-0.427261456	0.186431637	-2.291786216	0.021917982
31160.38115	-0.427203545	0.150786653	-2.833165507	0.004608952
2646.667531	-0.42691638	0.245615536	-1.738148926	0.082184576
9895.949843	-0.425234467	0.191121193	-2.22494669	0.026084817
40544.9927	-0.424688616	0.171342178	-2.478599377	0.013189936
18282.02138	-0.422920107	0.237300011	-1.782216967	0.074713857
9200.386066	-0.422807705	0.228658425	-1.849079935	0.064446271
2798.634338	-0.418100776	0.20989912	-1.991912954	0.046380609
13039.50432	-0.41774395	0.154121902	-2.710477512	0.00671864
15561.56944	-0.416354062	0.196392113	-2.120014166	0.034004851
16130.05104	-0.415589846	0.237411059	-1.750507527	0.080030777
61958.44269	-0.414862456	0.168542159	-2.461475856	0.013836671
2712.276952	-0.414771215	0.234983154	-1.76511043	0.077545208
5979.299181	-0.412675708	0.166313725	-2.481308799	0.013090092
16692.88702	-0.411354537	0.176612543	-2.329135463	0.019851891
21034.10063	-0.410730668	0.175060134	-2.346226175	0.018964595
31695.37885	-0.409442677	0.166686075	-2.456370024	0.014034856
14611.69711	-0.407303455	0.162198344	-2.511144359	0.012034047
13099.12859	-0.406322616	0.204184108	-1.989981587	0.046592964
3603.933506	-0.404579026	0.19799241	-2.043406741	0.041012191
7166.299521	-0.404521372	0.144806609	-2.793528376	0.005213645
8472.84657	-0.403555847	0.209110618	-1.929867794	0.053623221
10457.36852	-0.402371387	0.186456248	-2.157993588	0.03092833
10282.2711	-0.40224522	0.197993086	-2.031612459	0.042192904
8194.12451	-0.398126774	0.156634869	-2.54175061	0.011029884
6648.484972	-0.397906129	0.167246411	-2.379160949	0.017352098
16171.50904	-0.397493592	0.197234317	-2.015336876	0.043869364
30388.6422	-0.394606075	0.222922982	-1.770145323	0.076702935
3026.228621	-0.391172692	0.219120169	-1.785197107	0.074229351
17845.3095	-0.389944963	0.213077007	-1.830065892	0.067240086
49282.77554	-0.387649412	0.186366552	-2.080037474	0.037522096
12845.18747	-0.386360493	0.170234924	-2.269572446	0.023233537

76674.93979	-0.386306795	0.182757846	-2.113763119	0.034535504
25331.88943	-0.385149441	0.178423047	-2.158630551	0.030878841
4607.519144	-0.382100041	0.174783035	-2.186139179	0.028805424
14184.41779	-0.380432163	0.211664209	-1.797338171	0.07228195
57495.4401	-0.380298807	0.170348391	-2.232476656	0.025583476
6466.572648	-0.380219556	0.173449927	-2.192099839	0.028372298
36084.51881	-0.379696824	0.170023395	-2.233203394	0.025535535
16850.76174	-0.378980395	0.162994256	-2.325115035	0.02006581
8964.276606	-0.377045669	0.191908413	-1.964716727	0.049447033
5688.832946	-0.376110091	0.215554931	-1.744845688	0.081011734
22893.45727	-0.376017548	0.184297784	-2.040271676	0.041323274
7966.297062	-0.375477469	0.193660485	-1.938843998	0.052520337
18999.36531	-0.375469002	0.192043499	-1.955124775	0.050568345
25674.13855	-0.374891051	0.204811557	-1.830419418	0.067187246
3612.888504	-0.374315428	0.211623092	-1.76878347	0.076930016
11853.93535	-0.373861091	0.218557972	-1.710580896	0.087158508
5979.888588	-0.373666604	0.167746135	-2.227572063	0.025909065
18360.26259	-0.370696675	0.200606878	-1.847876192	0.064620255
11982.08406	-0.36973928	0.20790134	-1.778436251	0.075332233
59679.262	-0.369623846	0.173172906	-2.134420757	0.032808354
6660.174305	-0.368473587	0.209727891	-1.756912661	0.078932697
59140.29142	-0.368113928	0.16033921	-2.295844713	0.021684762
36632.83857	-0.366544556	0.15960001	-2.296644952	0.021639032
14560.11199	-0.366437812	0.196771302	-1.862252308	0.062567534
35695.77579	-0.365150343	0.189877157	-1.923087268	0.054469091
49599.3706	-0.365070549	0.194552917	-1.876458885	0.060592295
51405.8257	-0.365064232	0.154471592	-2.363309829	0.01811252
30968.49216	-0.362458898	0.162969099	-2.224095861	0.026141995
16427.16722	-0.362135617	0.144385687	-2.508112988	0.012137785
20664.35268	-0.360589054	0.178988169	-2.014597144	0.043946877
18898.09526	-0.360016301	0.151154988	-2.381769245	0.01722969
17587.63291	-0.359712232	0.142296688	-2.527903046	0.011474603
19347.61922	-0.359368794	0.152092976	-2.36282308	0.018136325
172982.0419	-0.35874929	0.206973958	-1.733306416	0.083041221
13582.76615	-0.358193999	0.166858142	-2.146697761	0.031817351
64709.94386	-0.35675313	0.210148997	-1.69761995	0.089579515
4223.276403	-0.3562393	0.172280541	-2.067786062	0.038660145
16237.91209	-0.355612656	0.171489074	-2.073675295	0.038109477
8648.968845	-0.354512213	0.179157853	-1.978770155	0.04784189
12476.0957	-0.354334733	0.187114146	-1.893682226	0.058267202
223090.8278	-0.353716449	0.181475346	-1.949115716	0.051281607
14482.93113	-0.352500395	0.16801939	-2.097974495	0.035907398
3999.223911	-0.351253672	0.205613217	-1.708322438	0.08757653
10844.83589	-0.349978151	0.135286681	-2.586937221	0.009683323
10932.66051	-0.349772679	0.155455207	-2.249990116	0.024449573
9858.850097	-0.34790971	0.17257873	-2.015947799	0.043805435
11846.71922	-0.34702607	0.184063655	-1.885359005	0.059381385
5202.340509	-0.345125603	0.196068909	-1.760226057	0.078369486
11050.64248	-0.344748088	0.149272042	-2.30952885	0.020914253
34929.84383	-0.343274274	0.150026629	-2.288088959	0.022132341
78784.04319	-0.342390202	0.181421067	-1.887268153	0.059124268
12073.15176	-0.341391233	0.206837954	-1.650525097	0.098835585
16350.79815	-0.340259652	0.152397837	-2.232706565	0.025568301
22975.07405	-0.339443446	0.179974419	-1.886064965	0.059286201
19393.02685	-0.339428121	0.17226418	-1.970392927	0.048793356
15564.12569	-0.339045994	0.163660016	-2.071648305	0.038298252
6804.712652	-0.33765654	0.181125759	-1.86421049	0.062292151
62034.46765	-0.33764248	0.162873574	-2.073034149	0.038169102

8555.3559	-0.337513036	0.1739901	-1.939840461	0.052399082
3428.737474	-0.337401955	0.201965271	-1.670593925	0.094801913
3428.737474	-0.337401955	0.201965271	-1.670593925	0.094801913
56169.52526	-0.337036533	0.171905422	-1.960592797	0.049926543
5736.754334	-0.336736657	0.152888899	-2.202492527	0.027630536
16985.33001	-0.336162426	0.149733095	-2.245077661	0.024763147
33473.6436	-0.334706871	0.146768937	-2.280502118	0.022577925
14022.16143	-0.334062587	0.153860406	-2.171205675	0.029915629
16740.21039	-0.333142987	0.146094069	-2.280332047	0.022588002
11537.00563	-0.332912772	0.182866936	-1.820519224	0.068679971
36349.1417	-0.332829586	0.201891465	-1.648556988	0.099238427
10239.55359	-0.33092189	0.143028937	-2.313670909	0.020685781
4645.451048	-0.330377107	0.193040852	-1.711436232	0.087000614
6240.928692	-0.329598594	0.180017983	-1.830920381	0.067112428
11143.56489	-0.329231511	0.151058487	-2.179496945	0.029294771
15009.5915	-0.32867075	0.188594963	-1.742733451	0.081380186
38542.13132	-0.328028683	0.178735303	-1.835276401	0.066464746
40126.80286	-0.327620432	0.150981062	-2.169943875	0.030011098
21515.56992	-0.32670861	0.158007425	-2.067678851	0.038670232
100364.4906	-0.325449867	0.174140786	-1.868889385	0.061638205
15862.21589	-0.324883207	0.155789969	-2.085392335	0.037033702
31722.65833	-0.324832689	0.145154024	-2.237848319	0.025230948
19793.77998	-0.324792943	0.145506522	-2.232153841	0.025604797
3609.374844	-0.324449975	0.190156689	-1.706224361	0.087966315
17844.44144	-0.323225404	0.160390672	-2.015238169	0.0438797
8205.777863	-0.323116948	0.171653388	-1.882380248	0.059784406
15249.28451	-0.321102083	0.188329532	-1.705001228	0.088194195
45950.85441	-0.317305046	0.181848833	-1.744883597	0.081005133
45950.85441	-0.317305046	0.181848833	-1.744883597	0.081005133
51471.97673	-0.3141609	0.157216286	-1.998271988	0.045687181
8278.261969	-0.312057877	0.150586926	-2.072277357	0.038239583
10820.72834	-0.311234118	0.167671388	-1.856214835	0.063422942
9985.560367	-0.30842497	0.14922332	-2.066868435	0.038746553
1142179.443	-0.307561278	0.185586904	-1.657235892	0.097471793
26063.41164	-0.306253654	0.155984777	-1.963356048	0.049604817
6487.680169	-0.304970177	0.176968936	-1.723297796	0.084834686
59384.91098	-0.30260283	0.157784567	-1.917822738	0.055133492
12699.70525	-0.298134927	0.161368341	-1.847542862	0.064668502
64685.65786	-0.295073618	0.155762302	-1.894384035	0.058174054
13218.50077	-0.292908907	0.165253717	-1.772479992	0.076314914
6237.650655	-0.292587948	0.153110905	-1.910954337	0.056010449
6612.32616	-0.291611714	0.144530838	-2.017643555	0.043628397
7138.71004	-0.289853028	0.159228845	-1.820355017	0.068704958
6528.610133	-0.288690956	0.154086937	-1.873558921	0.060991244
17065.00446	-0.288598593	0.141342681	-2.041836141	0.041167788
14888.29143	-0.287450415	0.161878149	-1.775720911	0.075778929
11619.94781	-0.286763275	0.159544274	-1.797389956	0.072273734
36265.03487	-0.285948259	0.16962155	-1.685801472	0.091834044
25271.16087	-0.284650288	0.165621385	-1.718680759	0.085672517
10630.11485	-0.283326576	0.15158477	-1.869096591	0.061609377
188159.5644	-0.282350834	0.170602808	-1.655018683	0.097920708
25320.09076	-0.281019157	0.166190561	-1.690945358	0.090847239
16441.28124	-0.278120076	0.167290635	-1.662496385	0.096413288
12022.02809	-0.275845798	0.152533261	-1.808430484	0.070539527
72872.36339	-0.273816511	0.151109632	-1.812038772	0.069980206
19671.83529	-0.268446828	0.149985188	-1.789822266	0.073482489
11363.14268	-0.268306339	0.155895688	-1.721063247	0.085239348
9686.468721	-0.26786122	0.157197297	-1.703981081	0.088384622

4444.341892	-0.265403124	0.15267489	-1.738354779	0.08214832
15796.04095	-0.263674258	0.155947325	-1.690790512	0.09087682
73403.42448	-0.262995433	0.159711752	-1.646688048	0.099622182
25445.55635	-0.257680764	0.136742163	-1.884428028	0.059507102
10393.38094	-0.252640104	0.148657999	-1.699471977	0.08923029
7705.906483	-0.243794611	0.135708543	-1.796457359	0.072421809
22558.0572	-0.23953181	0.143702347	-1.66686081	0.095542085
8920.479828	0.240961432	0.144850014	1.663523703	0.09620765
23630.59694	0.241590675	0.132585087	1.822155727	0.06843136
8287.53185	0.245521681	0.145736081	1.684700716	0.092046328
76778.88503	0.2522025	0.148963392	1.693050194	0.090445914
6235.612627	0.256328795	0.144751157	1.770823812	0.076590005
26561.63021	0.258939777	0.153644624	1.685316217	0.091927578
25663.33597	0.263237265	0.143882971	1.829523417	0.067321235
17013.71143	0.265270373	0.153648492	1.726475603	0.084261885
19722.29142	0.265864105	0.160681941	1.654598538	0.09800596
9780.227976	0.268257043	0.149994733	1.788443077	0.073704552
19861.00592	0.272947254	0.157511389	1.73287313	0.083118221
9455.489537	0.275939971	0.152901098	1.804695803	0.071122297
4793.386372	0.279277889	0.158613691	1.760742645	0.078281971
14621.27154	0.279491483	0.145437765	1.921725642	0.054640289
104431.7567	0.279899387	0.139740614	2.002992397	0.045178105
7794.748562	0.28138121	0.160212633	1.756298514	0.079037451
377484.9776	0.285512314	0.15971357	1.7876522	0.073832138
25532.37995	0.285545489	0.17167659	1.66327564	0.096257273
1785.538465	0.287425393	0.173600186	1.65567445	0.097787764
1785.538465	0.287425393	0.173600186	1.65567445	0.097787764
14614.75168	0.287850089	0.144959303	1.985730357	0.047063272
11025.52599	0.287884889	0.156227413	1.842729669	0.06536849
5010.823912	0.289144351	0.154316939	1.873704554	0.060971158
7011.330856	0.289367622	0.15816574	1.8295215	0.067321522
6317.839775	0.289654789	0.144830989	1.999950364	0.045505624
3721.566865	0.289777205	0.166970871	1.735495565	0.082653066
25348.08226	0.290554447	0.165679884	1.753709862	0.079480235
18258.03725	0.290897741	0.151215021	1.92373575	0.054387714
6559.832468	0.291767861	0.171292684	1.703329379	0.088506446
26593.31055	0.294111509	0.175293278	1.677825373	0.093381195
7937.819957	0.297531308	0.170173515	1.748399619	0.080394854
18035.7601	0.300572732	0.142808418	2.104726996	0.035315079
12903.98851	0.301364804	0.182706315	1.649449307	0.09905562
6089.528876	0.301662945	0.173000562	1.743710776	0.081209535
9414.599748	0.302402436	0.167359993	1.806897994	0.070778185
26319.81078	0.303300196	0.154547629	1.96250306	0.049703945
5132.135325	0.303500082	0.146841861	2.066849871	0.038748303
5532.567562	0.303795088	0.174797723	1.737980805	0.082214196
3763.515964	0.305701095	0.17603197	1.736622587	0.08245381
1710.523374	0.306719994	0.184842188	1.659361414	0.097042987
45802.37168	0.307167893	0.143242241	2.144394635	0.03200128
7382.485628	0.30994134	0.164152028	1.88813591	0.059007707
39398.44808	0.310129708	0.175607066	1.766043445	0.07738856
11592.97021	0.310524304	0.14627447	2.122887916	0.033763245
68449.16846	0.311480072	0.151801184	2.051894877	0.04017988
13446.7086	0.312832189	0.150703451	2.075813035	0.037911246
8518.964002	0.316417468	0.180155128	1.756361154	0.079026761
1956.253316	0.318521953	0.188955106	1.685701756	0.091853258
21732.35898	0.319071003	0.185372016	1.721246875	0.085206035
47197.01402	0.322000485	0.149060281	2.16020313	0.030756948
13240.83742	0.322239717	0.187094411	1.722337484	0.085008401

4152.548398	0.323596017	0.182296627	1.77510699	0.075880223
21749.14599	0.324563645	0.185575919	1.748953456	0.080299065
8490.099866	0.325438049	0.150951524	2.15591099	0.031090616
313401.0173	0.326354335	0.182435811	1.788872116	0.073635413
20021.49639	0.327505721	0.174470349	1.877142581	0.060498555
141337.6563	0.327535706	0.151733331	2.158627279	0.030879095
43321.44595	0.331223674	0.142333768	2.327091309	0.019960406
8814.203362	0.331281348	0.180991042	1.830374277	0.067193991
147424.6721	0.332848063	0.142631826	2.333617068	0.019615782
104430.4083	0.333546789	0.182090648	1.831762328	0.066986838
510062.0658	0.334298619	0.200242159	1.669471709	0.095023933
17765.53469	0.334476319	0.196096489	1.705672146	0.088069139
490509.1591	0.335439772	0.169529835	1.978647432	0.047855715
291148.0305	0.336521741	0.148061819	2.272846194	0.02303545
3701.567891	0.337070464	0.175004891	1.926063105	0.054096495
483664.39	0.337666434	0.163239837	2.068529604	0.03859025
23739.83843	0.338075925	0.170262623	1.985614454	0.04707615
16268.9679	0.338465532	0.162244796	2.086141062	0.036965847
7579.061624	0.339017522	0.20532736	1.651107397	0.098716647
2137.422943	0.342047082	0.182030411	1.879065589	0.060235538
11343.80627	0.342360804	0.201755161	1.696912251	0.089713252
340839.0388	0.344276579	0.148204752	2.322979352	0.020180262
513137.9728	0.347037349	0.154820345	2.24154874	0.024990552
13257.59334	0.349497792	0.148500447	2.353513395	0.018596938
13257.59334	0.349497792	0.148500447	2.353513395	0.018596938
5729.215867	0.349852505	0.182232932	1.91980945	0.054881973
79463.17624	0.351223103	0.159483916	2.202247795	0.02764781
12876.7606	0.351459399	0.208351394	1.686858875	0.091630491
10616.17896	0.351471073	0.195347872	1.799206045	0.071986094
394729.7518	0.352840047	0.193930694	1.819413107	0.068848429
4644.214649	0.354017087	0.212327875	1.667313285	0.095452126
16243.58895	0.354724515	0.203474627	1.743335374	0.08127505
9581.766756	0.355560798	0.195437222	1.819309515	0.068864223
9581.766756	0.355560798	0.195437222	1.819309515	0.068864223
5763.564462	0.355590927	0.197176272	1.803416423	0.07132284
9146.042409	0.355813944	0.210468136	1.690583432	0.090916391
233946.5731	0.355894733	0.136434241	2.608544083	0.00909283
18034.02132	0.359043353	0.161685294	2.220630851	0.026375974
19706.27528	0.362580229	0.209411251	1.731426689	0.08337569
24522.0056	0.363465374	0.207076162	1.755225563	0.079220733
8608.422176	0.363876009	0.178070208	2.043441256	0.041008777
38151.66114	0.365254976	0.170231133	2.145641462	0.031901595
283211.5274	0.365874839	0.161781227	2.261540755	0.023725792
39988.44661	0.365943818	0.191823904	1.907707071	0.056429087
185856.6444	0.366161093	0.141183091	2.593519456	0.009499916
14918.08794	0.367155092	0.163588387	2.244383592	0.024807731
18638.69214	0.36977419	0.168164147	2.198888378	0.02788586
13902.69896	0.371202953	0.212610713	1.745927789	0.0808235
5361.261899	0.372552934	0.156470022	2.380986018	0.017266367
18351.30622	0.374061904	0.154089102	2.427568853	0.015200401
75166.26373	0.374572965	0.20975618	1.785754133	0.074139076
4036.954421	0.376109681	0.175901587	2.138182422	0.03250194
491463.1564	0.376307413	0.154231442	2.4398878	0.014691824
56856.53835	0.379359649	0.156394134	2.425664186	0.015280402
529938.3563	0.379754636	0.156885731	2.420581104	0.015495722
39610.03393	0.380161602	0.1547219	2.45706395	0.014007775
9039.142481	0.380718554	0.170780453	2.229286466	0.025794851
18308.92527	0.386780112	0.204674667	1.88973124	0.058793914

601118.2388	0.387023533	0.146665679	2.638814593	0.008319647
262929.5909	0.387733067	0.176283302	2.199488334	0.027843218
11092.57027	0.388019599	0.183049766	2.119749219	0.0340272
4021.310861	0.388578092	0.176472623	2.201917127	0.027671163
4021.310861	0.388578092	0.176472623	2.201917127	0.027671163
4021.310861	0.388578092	0.176472623	2.201917127	0.027671163
40438.35783	0.388732695	0.161526269	2.406622133	0.016100819
10480.11257	0.389769703	0.188422963	2.06858918	0.038584654
11088.98212	0.39141121	0.232090368	1.686460379	0.09170716
396671.6383	0.392511698	0.162120819	2.421106064	0.015473362
52893.00793	0.393383936	0.213304959	1.844232492	0.065149265
100414.5484	0.394133508	0.194074195	2.030839333	0.042271296
15983.06843	0.395693669	0.168680911	2.345811787	0.018985692
2264.914814	0.399105129	0.213443968	1.86983559	0.061506652
4633.868669	0.400568416	0.20463905	1.957438796	0.050295901
16027.91546	0.404053646	0.175849275	2.297727107	0.021577326
83677.55861	0.405070346	0.132411846	3.059169993	0.002219512
2449.081765	0.406740761	0.23804458	1.708674744	0.087511215
3553.874713	0.40797114	0.183864187	2.218872239	0.026495418
5702.314093	0.408072666	0.207158512	1.969857099	0.04885475
158370.5692	0.408112143	0.172527996	2.365483593	0.018006541
32785.85294	0.408278822	0.203972379	2.00163779	0.045323702
24282.60519	0.408578361	0.177466317	2.302286808	0.021319003
749331.6054	0.409422556	0.1901425	2.153240632	0.031299772
10590.98344	0.410872789	0.156967892	2.617559452	0.008856106
308757.6412	0.411568502	0.169813943	2.423643747	0.015365671
6246.112492	0.412598662	0.182069271	2.266163089	0.0234414
3538.518383	0.414388249	0.208660697	1.985942992	0.047039654
213464.001	0.415268814	0.164998283	2.516806876	0.011842372
196825.2835	0.416182011	0.216315365	1.923959548	0.054359654
59313.23621	0.4163016	0.231045167	1.801819118	0.071573869
1968.920509	0.416303722	0.239269406	1.739895332	0.081877399
1968.920509	0.416303722	0.239269406	1.739895332	0.081877399
1968.920509	0.416303722	0.239269406	1.739895332	0.081877399
13750.54869	0.419266824	0.23744736	1.765725352	0.077441937
426732.2153	0.419649437	0.16480823	2.546289321	0.010887491
252644.7434	0.420974441	0.153256818	2.746856194	0.006016951
188568.7998	0.422320133	0.155478746	2.716256358	0.006602477
5925.07129	0.422424518	0.17058704	2.476299005	0.013275235
884621.7302	0.423248804	0.177906115	2.379057091	0.017356987
698408.8382	0.423252959	0.191276464	2.212781177	0.026912736
3917.442595	0.423551882	0.216466577	1.956661798	0.050387244
430332.0644	0.424116597	0.191685867	2.212560603	0.026927954
1424529.292	0.424170976	0.196460793	2.159061716	0.030845379
7034.523903	0.425318624	0.176569354	2.408790736	0.016005472
16077.11432	0.426666499	0.228577548	1.866615958	0.061955238
2546.115763	0.426698128	0.241771779	1.764879797	0.07758397
3776.070263	0.426793736	0.180044645	2.370488362	0.017764603
16516.93201	0.427738774	0.191039082	2.239011884	0.025155143
9023.923925	0.427740604	0.13380403	3.196769222	0.001389761
200563.6258	0.429007629	0.193174385	2.220830828	0.026362422
107885.1566	0.42904583	0.165663472	2.589863811	0.009601391
4563.558259	0.42977439	0.19926446	2.15680403	0.031020937
11344.91686	0.431241943	0.234203431	1.841313519	0.065575628
11404.41427	0.431243278	0.20580826	2.095364283	0.036138622
21057.28581	0.432071789	0.180170795	2.398123341	0.016479315
28816.89104	0.433139026	0.173428492	2.497507891	0.012506968
35070.24305	0.43561773	0.217189168	2.005706525	0.044887569

12937.71011	0.436876342	0.152440501	2.865881048	0.004158505
5958.330807	0.438521644	0.249853378	1.755115927	0.07923948
6737.14207	0.439346692	0.258668797	1.698491263	0.08941508
103236.0843	0.442488725	0.187718722	2.357190162	0.018413815
253164.3903	0.443353958	0.18371541	2.413264937	0.015810322
21587.07834	0.444409955	0.177663483	2.501414179	0.012369842
3824.021955	0.444446422	0.187967892	2.364480538	0.018055376
27966.84211	0.446155117	0.219594891	2.031719018	0.042182109
180352.2197	0.448161424	0.17513577	2.558937124	0.010499273
3176.838004	0.448530039	0.220052006	2.03829107	0.041520832
83457.95806	0.44873539	0.265388185	1.690864234	0.090862736
2474.401756	0.448833109	0.192961393	2.326025437	0.020017194
2474.401756	0.448833109	0.192961393	2.326025437	0.020017194
2505.296275	0.451857429	0.24646669	1.833340762	0.066751911
10543.12695	0.45304505	0.14889928	3.042627531	0.002345224
28713.36363	0.454454203	0.206585148	2.199839667	0.027818273
11807.06106	0.45550401	0.1479726	3.078299707	0.002081854
22023.42405	0.457759305	0.204303094	2.240589195	0.025052697
9104.030257	0.457901967	0.233019433	1.965080601	0.04940491
21563.2718	0.459320885	0.216829266	2.118352809	0.034145199
101436.159	0.460263662	0.195056948	2.359637358	0.018292807
8609.709772	0.462209188	0.191355859	2.415443091	0.015716078
21570.94541	0.46238436	0.14580278	3.171300021	0.001517583
3142.333987	0.462848489	0.247906439	1.867028909	0.061897551
6931.558062	0.464888503	0.196185173	2.369641381	0.017805346
48385.6834	0.465059503	0.16985898	2.737915313	0.006183
6085.881193	0.468098846	0.198593115	2.357074898	0.018419531
7457.58466	0.468303301	0.169940737	2.755685949	0.00585692
3542.572473	0.469049364	0.275438104	1.702921116	0.088582832
12702.93874	0.470002897	0.187391718	2.508130565	0.012137181
4129.74602	0.471041973	0.282346381	1.668312414	0.095253725
2277.027376	0.471325443	0.207296344	2.273679471	0.022985265
9217.12414	0.472563043	0.239851257	1.970233761	0.048811586
47090.44562	0.473951304	0.271492168	1.745727354	0.08085834
5576.311408	0.473973713	0.238354905	1.988520914	0.046754108
4016.118283	0.475257483	0.268847128	1.767761057	0.077100858
52973.48272	0.479583559	0.269527923	1.779346472	0.075182976
4892.71178	0.484443978	0.205517815	2.357187277	0.018413958
32780.57577	0.485480355	0.191356823	2.537042295	0.011179345
86096.62098	0.490176647	0.200680614	2.442571	0.014583061
39464.40208	0.493084733	0.236310032	2.08660093	0.036924223
15044.5465	0.493818507	0.25565649	1.931570397	0.053412553
71782.6442	0.494826162	0.212391442	2.329783894	0.019817576
7127.088974	0.495377514	0.263707398	1.878512008	0.060311156
18087.13949	0.497115383	0.196931371	2.524307739	0.01159264
10388.97148	0.497280002	0.238416786	2.085759188	0.037000442
10342.85538	0.497631812	0.283299386	1.756558029	0.078993172
8384.67204	0.498718407	0.229253177	2.175404561	0.029599813
357545.7051	0.49931026	0.202334949	2.467741055	0.013596864
108797.4663	0.503535206	0.208636605	2.413455712	0.015802048
25741.52187	0.504296595	0.221961599	2.271999288	0.023086553
4649.330146	0.504653353	0.213418458	2.364619058	0.018048625
8305.337433	0.504795171	0.267199665	1.88920585	0.058864251
30954.09431	0.506677533	0.218725224	2.316502527	0.020530847
55840.55651	0.509035409	0.202038895	2.51949214	0.011752426
1846.406051	0.509900552	0.214043009	2.382234084	0.017207955
8321.223104	0.5110705	0.192990038	2.648170377	0.008092873
24946.78574	0.51142938	0.289274079	1.767975136	0.07706506

112060.3702	0.51178007	0.172482933	2.967134557	0.003005894
12064.29784	0.512169905	0.144161585	3.55274885	0.000381228
70488.86614	0.512373636	0.22341369	2.293385134	0.021825842
19198.56108	0.514903497	0.18188763	2.830887927	0.004641898
104135.8761	0.515592536	0.243800862	2.11481014	0.034446132
4823.822166	0.51657364	0.143001826	3.612356953	0.000303426
71136.55234	0.519862093	0.213444884	2.435580015	0.014867936
8656.059923	0.520772069	0.199028838	2.616565899	0.008881922
3617.775141	0.522033681	0.30924585	1.688086292	0.091394665
801.9191863	0.523530043	0.261317616	2.003424227	0.045131773
7566.242131	0.527108238	0.240911091	2.187978294	0.028671183
63500.95234	0.530140728	0.217936621	2.432545413	0.014993111
6794.479854	0.533659799	0.246537989	2.16461488	0.030417191
64053.17235	0.536214383	0.176277463	3.041877128	0.002351078
5696.419939	0.538496695	0.206090641	2.612911931	0.008977443
18823.73597	0.540335021	0.179592689	3.00866937	0.002623945
173938.4075	0.544695636	0.235159656	2.316280121	0.020542979
6210.636138	0.548876765	0.240459213	2.282618988	0.022452821
3440.030171	0.550605384	0.209502101	2.628161627	0.008584772
21279.48344	0.551235305	0.203550629	2.708099248	0.006766978
13778.85762	0.554011576	0.151440415	3.65828089	0.000253913
414134.2038	0.55420318	0.25393572	2.1824546	0.029075998
88284.97039	0.555293665	0.197966586	2.804986823	0.005031864
18526.44824	0.557072188	0.220499964	2.526404892	0.011523659
8448.735802	0.557393132	0.202247908	2.75598961	0.005851486
28085.51107	0.558846444	0.218304544	2.559939585	0.010469036
4051.141793	0.559442243	0.192647755	2.903964508	0.003684699
10749.40682	0.561358276	0.239979749	2.339190198	0.019325592
8539.14161	0.56261529	0.163002981	3.451564422	0.000557347
37445.81017	0.562743114	0.177866257	3.163855376	0.001556941
522172.8883	0.563690675	0.190102398	2.965194976	0.003024911
1272.313515	0.564346905	0.275227618	2.050473386	0.040318258
976.8781246	0.564594374	0.283832745	1.989179842	0.046681356
976.8781246	0.564594374	0.283832745	1.989179842	0.046681356
528858.9412	0.565356471	0.225104698	2.511526756	0.012021017
17283.91612	0.567373855	0.343612817	1.651201081	0.098697522
4060.020874	0.569350018	0.16558769	3.438359567	0.00058525
63660.19906	0.569430629	0.151638987	3.755172986	0.000173222
17715.9365	0.573146155	0.217933132	2.629917493	0.008540559
6247.969968	0.57405044	0.177776437	3.229058087	0.001241987
235549.2234	0.576764545	0.2310454	2.496325589	0.012548736
5086.504834	0.579369579	0.27938459	2.073734913	0.038103937
6770.780968	0.580327252	0.234691359	2.472725266	0.013408718
97638.17269	0.587089359	0.208589321	2.814570539	0.004884246
9413.370842	0.588479671	0.224641823	2.619635398	0.008802382
4340.237356	0.589624532	0.235832515	2.500183373	0.012412904
70175.76618	0.590798926	0.240725023	2.454248083	0.014117955
7444.63776	0.591442667	0.232040209	2.548880085	0.010806945
10236.72516	0.598731489	0.207983776	2.878741314	0.003992657
1714.909601	0.599228907	0.348954257	1.717213345	0.085940198
1714.909601	0.599228907	0.348954257	1.717213345	0.085940198
1714.909601	0.599228907	0.348954257	1.717213345	0.085940198
10780.7791	0.599936014	0.232590037	2.57937108	0.00989804
4635.231657	0.601337778	0.206178093	2.916593948	0.003538761
26158.16767	0.606791274	0.193845834	3.130277604	0.001746412
54025.59347	0.607560459	0.179347118	3.387623213	0.00070501
3060.04951	0.609005541	0.311197407	1.956974983	0.05035041
22885.35309	0.610800683	0.248051355	2.462396073	0.013801216

96576.67859	0.614434197	0.248537127	2.472202861	0.013428329
42837.41845	0.621269813	0.232010388	2.677767223	0.007411471
7573.631405	0.645496492	0.157434067	4.100106817	4.13E-05
145745.6498	0.651195596	0.177012024	3.678821253	0.000234314
9039.00482	0.651323713	0.306594986	2.12437823	0.033638529
2375.002104	0.651749555	0.278333204	2.341616258	0.019200445
1491.521828	0.653622182	0.214353474	3.049272631	0.002293962
26793.60879	0.662603353	0.225535225	2.93791514	0.003304275
159989.0662	0.664375273	0.194783431	3.410840796	0.000647629
1045.809268	0.665929605	0.325918686	2.043238491	0.041028835
16019.35979	0.667264255	0.296280899	2.252133897	0.024313812
170865.4887	0.667543342	0.263016535	2.538028043	0.011147905
112524.2006	0.668833277	0.223486323	2.992725759	0.002764981
2099.366473	0.670329742	0.189271129	3.541637568	0.000397651
45777.22933	0.674074192	0.204866434	3.290310558	0.001000769
5174.053519	0.676569736	0.329205645	2.055158366	0.039863711
42568.23472	0.681027756	0.181804352	3.745937597	0.000179721
5349.266144	0.682314243	0.282607958	2.414349009	0.015763355
840614.7634	0.684176674	0.208064191	3.288296122	0.001007958
175274.596	0.686923267	0.32200695	2.133256031	0.032903729
8190.529176	0.689767602	0.389025579	1.773064907	0.076217952
20295.12674	0.689829381	0.286461666	2.408103643	0.016035628
13922.31799	0.690154222	0.280854938	2.457333406	0.013997271
7469.906809	0.692497104	0.326053367	2.123876563	0.033680467
2806.514322	0.692646331	0.175092273	3.9558932	7.62E-05
2806.514322	0.692646331	0.175092273	3.9558932	7.62E-05
2806.514322	0.692646331	0.175092273	3.9558932	7.62E-05
220660.4505	0.693939445	0.205575609	3.375592311	0.00073657
8949.380044	0.695283795	0.281784484	2.467431082	0.013608641
30036.63464	0.702477002	0.29799343	2.357357344	0.018405526
104778.4269	0.709713564	0.338357029	2.097528655	0.035946803
12813.68892	0.715309834	0.204346343	3.500477776	0.000464425
2147.940875	0.716120532	0.249924637	2.865345887	0.00416554
47737.36719	0.71649419	0.266422262	2.689318019	0.007159817
9441.104048	0.730353373	0.241793945	3.020561055	0.002523068
9974.70245	0.734283966	0.265934783	2.761142999	0.005759945
1396.180465	0.741289639	0.240915045	3.076975284	0.002091126
1396.180465	0.741289639	0.240915045	3.076975284	0.002091126
44530.68358	0.743180871	0.215119461	3.454735653	0.000550833
4165.263791	0.751229015	0.216619783	3.467961265	0.000524423
2531.215155	0.751342253	0.309850005	2.424857967	0.015314377
9649.099194	0.752121765	0.203521365	3.695542069	0.000219418
348653.4439	0.783231566	0.248428918	3.152739113	0.001617463
20655.85401	0.786902335	0.211069547	3.72816613	0.000192878
31172.6643	0.790892368	0.292889348	2.700311135	0.006927466
77656.24512	0.791157609	0.299812158	2.638844312	0.008318918
19447.87346	0.793834859	0.219947196	3.609206548	0.000307135
2419625.624	0.799654205	0.303364486	2.635951937	0.008390161
41505.56675	0.803072505	0.174199291	4.610079075	4.03E-06
240545.0096	0.820118097	0.255213848	3.21345453	0.001311486
350247.5737	0.830420885	0.302494773	2.745240441	0.006046658
17808.9198	0.841786847	0.370110414	2.27442086	0.022940694
40248.76193	0.855905611	0.277532292	3.08398567	0.002042474
2973.504364	1.076004047	0.320579583	3.356433484	0.000789547
2973.504364	1.076004047	0.320579583	3.356433484	0.000789547
74610.23062	1.258841053	0.26159001	4.812267312	1.49E-06

padj
0.067924242
0.112311266
0.049804799
0.402928546
0.402928546
0.402928546
0.098521619
0.000931389
0.097789798
0.198211487
0.248871712
0.187362784
0.187362784
0.04006574
0.276989038
0.035913963
0.078509978
0.285444573
0.285444573
0.285444573
0.285444573
0.133360855
0.149276096
0.028717157
0.086521044
0.26592578
0.279453861
0.285260795
0.260626228
0.015258241
0.042610037
0.086637289
0.030007139
0.387267638
0.387267638
0.015258241
0.305293057
0.305293057
0.305293057
0.323814076
0.042610037
0.290338288
0.26592578
0.059100584
0.341302089
0.253594361
0.000931389
0.063263829
0.215303637
0.254999071
0.157185246
0.264628993
0.422742649
0.106851479
0.381211902
0.381211902
0.125700516

0.016766155
0.167859355
0.262740279
0.058298292
0.341302089
0.201314687
0.232767088
0.140934095
0.444868154
0.167859355
0.198208132
0.285260795
0.285260795
0.198211487
0.079564834
0.050097809
0.269771525
0.157185246
0.26096027
0.349162964
0.04006574
0.198211487
0.3831713
0.198211487
0.116963244
0.029528921
0.043211874
0.254670213
0.285444573
0.445429323
0.356797101
0.1900508
0.331160517
0.331160517
0.295492255
0.295771597
0.285444573
0.273635268
0.426108994
0.342560733
0.436146505
0.26592578
0.356797101
0.345708561
0.223997302
0.367284244
0.264628993
0.060203261
0.311160846
0.252727572
0.411454736
0.186254181
0.26592578
0.293118381
0.262935639
0.201314687
0.353311598
0.353311598

0.353311598
0.222790732
0.206306631
0.377674123
0.377674123
0.260626228
0.186254181
0.412921523
0.445429323
0.157854362
0.198208132
0.198211487
0.293118381
0.254670213
0.086521044
0.328472848
0.116963244
0.31170031
0.253594361
0.232636145
0.373076233
0.308466879
0.345708561
0.431472301
0.117788806
0.372221172
0.167482185
0.402928546
0.086521044
0.155567057
0.342595125
0.240072769
0.248232723
0.240072769
0.278975487
0.278975487
0.402928546
0.293118381
0.349162964
0.26592578
0.238019003
0.149276096
0.455546246
0.26096027
0.26096027
0.402928546
0.152354823
0.3831713
0.111257501
0.406951714
0.319572318
0.224164829
0.402928546
0.093821055
0.118286963
0.427706865
0.26592578
0.26592578

0.26592578
0.213168231
0.231063597
0.402689322
0.427706865
0.198211487
0.375808626
0.186254181
0.293118381
0.273635268
0.421110607
0.426108994
0.253594361
0.406951716
0.253594361
0.086521044
0.249449095
0.345708561
0.141838029
0.379262269
0.457241086
0.24995273
0.293118381
0.364774449
0.345708561
0.125700516
0.260767791
0.149276096
0.427935431
0.269553018
0.232636145
0.420572977
0.396308103
0.341302089
0.18164583
0.293118381
0.427706865
0.232767088
0.424230192
0.232331244
0.253594361
0.252727572
0.232767088
0.223997302
0.341302089
0.319572318
0.157185246
0.362850461
0.285260795
0.323814076
0.219785669
0.249449095
0.331160517
0.424230192
0.418571063
0.402689322
0.308466879
0.262935639

0.294412909
0.285260795
0.278975487
0.412921523
0.267409584
0.276989038
0.267409584
0.253594361
0.348381698
0.427706865
0.320329343
0.35687459
0.34945954
0.402689322
0.424230192
0.440951456
0.268858288
0.396308103
0.421860507
0.290358402
0.426108994
0.260626228
0.260626228
0.387267638
0.364774449
0.3831713
0.25032066
0.269553018
0.223997302
0.331160517
0.249449095
0.222790732
0.25032066
0.429885322
0.285444573
0.444868154
0.308466879
0.308466879
0.343883547
0.379262269
0.353311598
0.302474656
0.441690325
0.204230092
0.26592578
0.331160517
0.381211902
0.426108994
0.256337708
0.26096027
0.381211902
0.465243447
0.267409584
0.381211902
0.345708561
0.308466879
0.386854504
0.308466879

0.356797101
0.457241086
0.457241086
0.349162964
0.273635268
0.26592578
0.262020829
0.284110999
0.262020829
0.402928546
0.465789609
0.254999071
0.440839302
0.402689322
0.279851227
0.427706865
0.402689322
0.284186724
0.308466879
0.385001714
0.305293057
0.266938501
0.267409584
0.442641597
0.331160517
0.381492632
0.442641597
0.427706865
0.427706865
0.338794436
0.308466879
0.390887508
0.308466879
0.463352778
0.348679076
0.435985855
0.366954059
0.396308103
0.379262269
0.423710837
0.371269264
0.331160517
0.402928546
0.3831713
0.319887501
0.422742649
0.412921523
0.446885612
0.436146505
0.385001714
0.463352778
0.446067128
0.459838998
0.409129257
0.406951716
0.417781855
0.435995908
0.442641597

0.427935431
0.446067128
0.466916086
0.381211902
0.444868154
0.412921523
0.457700137
0.459770032
0.402928546
0.446885612
0.446067128
0.424230192
0.446885612
0.402689322
0.43372837
0.463352778
0.417781855
0.429885322
0.411041318
0.426108994
0.365168022
0.337329848
0.426108994
0.417781855
0.459770032
0.463352778
0.463352778
0.341302089
0.399091833
0.3831713
0.402689322
0.338220289
0.428845303
0.426697195
0.364774449
0.442641597
0.452689731
0.427706865
0.298706711
0.465604421
0.427706865
0.409781722
0.348679076
0.308466879
0.427935431
0.42849596
0.462163667
0.285549881
0.381211902
0.424230192
0.293118381
0.316757888
0.308466879
0.426108994
0.446885612
0.435995908
0.285260795
0.435995908

0.422742649
0.427706865
0.285260795
0.417781855
0.3831713
0.285260795
0.253594361
0.402689322
0.253594361
0.402689322
0.457241086
0.442641597
0.343883547
0.262935639
0.364774449
0.308466879
0.341302089
0.305293057
0.465243447
0.3831713
0.444868154
0.254052287
0.26679069
0.250634249
0.250634249
0.366030077
0.273635268
0.446885612
0.412921523
0.402928546
0.457700137
0.427706865
0.402928546
0.402928546
0.411454736
0.446067128
0.198211487
0.269771525
0.430531385
0.426108994
0.319572318
0.285444573
0.264815712
0.372523731
0.202998213
0.26592578
0.273635268
0.427706865
0.249449095
0.240072769
0.418571063
0.289222747
0.238595225
0.240072769
0.240072769
0.232767088
0.268531008
0.381211902

0.198211487
0.273635268
0.293118381
0.273635268
0.273635268
0.273635268
0.240992909
0.308466879
0.446885612
0.240072769
0.398502472
0.323814076
0.252727572
0.385001714
0.349162964
0.260626228
0.101534844
0.441690325
0.269771525
0.345708561
0.25032066
0.337640786
0.26031625
0.285444573
0.198211487
0.240072769
0.264366896
0.341302089
0.223997302
0.364774449
0.411454736
0.427935431
0.427935431
0.427935431
0.424230192
0.218287473
0.167859355
0.180208789
0.232636145
0.249449095
0.27246727
0.349162964
0.27246727
0.285260795
0.240992909
0.385499256
0.424230192
0.25032066
0.266938501
0.083591531
0.269771525
0.203825604
0.285260795
0.399605326
0.303302957
0.245526678
0.225183951
0.336708598

0.140934095
0.426108994
0.444868154
0.25032066
0.240072769
0.225183951
0.25032066
0.323814076
0.214475714
0.321094437
0.446067128
0.253594361
0.253594361
0.402689322
0.103193281
0.273635268
0.098434471
0.26679069
0.348381698
0.293118381
0.25032066
0.240072769
0.086521044
0.385499256
0.25032066
0.170189682
0.25032066
0.167859355
0.442641597
0.223997302
0.457668785
0.262935639
0.345708561
0.427706865
0.341302089
0.424230192
0.421751826
0.25032066
0.220063706
0.238019003
0.305293057
0.36217948
0.253594361
0.3831713
0.222798188
0.305293057
0.426108994
0.281936049
0.232636145
0.240072769
0.262935639
0.25032066
0.381211902
0.254670213
0.223997302
0.249449095
0.198208132
0.424230192

0.116963244
0.043211874
0.260626228
0.149276096
0.294412909
0.04006574
0.240072769
0.198211487
0.446885612
0.337329848
0.278814383
0.240072769
0.285260795
0.103193281
0.198211487
0.110682771
0.254670213
0.262020829
0.198211487
0.18164583
0.039271828
0.279404861
0.155652316
0.222790732
0.167859355
0.214475714
0.13151542
0.253594361
0.050285056
0.086521044
0.116963244
0.317079184
0.341302089
0.341302089
0.223997302
0.465243447
0.05137546
0.035913963
0.198211487
0.079097503
0.225183951
0.308466879
0.232636145
0.15401971
0.198211487
0.225183951
0.232767088
0.218018374
0.139442469
0.436146505
0.436146505
0.436146505
0.206306631
0.127709964
0.090037231
0.058298292
0.349162964
0.232767088

0.232636145
0.187362784
0.016766155
0.038052661
0.293118381
0.253594361
0.103193281
0.123359583
0.055355231
0.319572318
0.26592578
0.220063706
0.112311266
0.043211874
0.067924242
0.315030008
0.035913963
0.240072769
0.067924242
0.290411173
0.423710837
0.240992909
0.232767088
0.293118381
0.022514316
0.022514316
0.022514316
0.058350698
0.232636145
0.25032066
0.302474656
0.048659751
0.140934095
0.186254181
0.107827973
0.167859355
0.098434471
0.098434471
0.050285056
0.050097809
0.240072769
0.037508954
0.086521044
0.035913963
0.184429578
0.198211487
0.04006574
0.198211487
0.003268429
0.080371798
0.167859355
0.262935639
0.098434471
0.059100584
0.059100584
0.001615637
