# Supplementary material for: CH02 peptide promotes ex vivo expansion of umbilical cord blood-derived CD34 + hematopoietic stem/progenitor cells : CH02 peptide promotes CD34 + UCB-HSPC ex vivo expansion
Source: Acta Biochim Biophys Sin (Shanghai). 2023 Jun 28;55(10):1630–9. doi: 10.3724/abbs.2023047 (PMC10577473; doi:10.3724/abbs.2023047)
Supplement: 23018Supplementary_Table_S2 [file 23018Supplementary_Table_S2.pdf]

Supplementary Table S2. Information of Cord blood samples and frequency of CD34+ cells after enrichment

Sample ID	Maternal age	Newborn sex	Volume（mL）	Monocyte numbers (10^8)	CD34+ cell numbers (10^6)	CD34+ cell frequency（%）
HQ-1	34	Male	40	2.50	3.28	42.0
HQ-2	32	Male	45	2.61	1.05	66.7
HQ-3	28	Male	30	1.77	2.43	61.1
HQ-4	35	Female	30	1.65	2.46	58.2
HQ-5	25	Female	45	3.00	3.20	52.2
HQ-6	30	Male	30	1.81	0.70	62.5
HQ-7	34	Male	50	1.28	2.10	80.1
HQ-8	26	Female	55	4.40	3.60	73.7
HQ-9	31	Female	60	2.50	2.40	85.1
HQ-10	28	Male	60	2.75	2.60	74.7
