# Supplementary material for: CH02 peptide promotes ex vivo expansion of umbilical cord blood-derived CD34 + hematopoietic stem/progenitor cells : CH02 peptide promotes CD34 + UCB-HSPC ex vivo expansion
Source: Acta Biochim Biophys Sin (Shanghai). 2023 Jun 28;55(10):1630–9. doi: 10.3724/abbs.2023047 (PMC10577473; doi:10.3724/abbs.2023047)
Supplement: 23018Supplementary_Table_S1-2 [file 23018Supplementary_Table_S1-2.pdf]

Tagln2
Cyba
Nefh
Map1b
Itpr2
Bcas1
Cldn11
Strn
Tspoap1
Rere
Thrap3
Marcks
Akap12
Map1a
Ptgr2
Acy3
Rpl15
Pak1
Ssr3
Gpat3
Trip12
Slc4a4
Glyr1
Rtn4
Snph
Dclk1
Nefm
Trim69
Prpf4b
Hspb1
Pclo
Mapre2
Map2
Cr1l
Plp1
Grasp
Sipa1l2
Phldb1
Tjp1
Lmna
Adgrl2
Ttc41
Stmn1
Stim1
Ppig
Sdc2
Sorbs2
Prpf38b
Znf423
Lsr
Apba1
Mapt
Ccdc8
Rps28
Cybc1
Washc2
Rhbdf1
Nefl

Trpv2
Lrp1
Pex19
Tmem100
Rap1b
Abi1
Recql
Hnrnpa3
Slc9a3r1
Cast
Ip6k1
Slc4a7
Nop58
Plekha4
Mpz
Marcksl1
Zdhhc5
Rtn1
Rgs18
Cacna1b
F11r
Slc20a2
Exoc2
Bscl2
Gja1
Tax1bp1
Pdap1
Cdk5rap2
Cds1
Zbtb38
Mta1
Chgb
Mtfr1l
Mff
Map4
Map3k12
Stx8
Arfgef1
Zbtb7a
Myo1e
Rplp2
Inpp5d
Adra2a
Anxa4
Brca2
Ednra
Arglu1
Pgrmc1
Ina
Sh3bp4
Eif2ak3
Mob1a
Cep162
Samd4a
Synrg
Crlf2
Mlip
Kif1b

Nsf
Cntnap5c
Snx17
Pelp1
Nup98
Pacs1
Smarca4
Mdc1
Mylpf
Rbm5
Ncam1
Necab1
Ncl
Epn3
Pom121
Klc1
Bzw1
Ccnl1
Septin7
Ap2m1
Myo9b
Orc2
Abcc5
Vim
Ubd
Ckm
Synpo
Taok1
Coro7
Eepd1
Pdha1
Raf1
Acaca
Elavl4
Hsph1
Scaf1
Eml1
Efnb1
Cavin1
Phrf1
Cox4i1
Rab10
Piezo1
Bsg
Rtn3
Drp2
Rab8a
Aqp1
Tmem245

Fxyd7
Ubr4
Aak1
Bckdha
Agap2
Cmklr1
Rps26
Ubac1

Dpysl3
Vmp1
Sgip1
Hnrnpu
Pea15
Cend1
Mindy1
Caskin1
Mertk
Brsk2
Map6
Ssbp3
Aldh2
Pex14
Serpina6
Pip4p2
Camsap2
JPT1
Ptdss2
Tra2b
Prkcb
Myl2
Psip1
Prx
Nptn
Stip1
Snn
Tsnax
Dbp
Magi3
Nmrk1
Mief1
Nnat
Ptger3
Ubap1
Ppfia3
Osmr
H1-4
Rpl4
Mast1
Oxr1
Ttgn1
Cap2
Batf3
Thra
Dlg4
Dab2ip
Hnrnpk
Ehd2
Lmnb1
Ptbp2
Tsc2
Srsf2
Itgb4
Ank3
Rpl18a
Ptov1
Ddx1

Rpl13
H1-5
Rab3gap2
Rplp0
Rheb
Sh3kbp1
Pxn
Prkaa1
Cacnb2
Palld
Tmem230
Rmdn2
Mark1
Irs1
Ufl1
Rps8
Dnah12
Pard3
Ndrg1
Rabep2
Mpdz
Micall1
Pi4k2b
Spp1
Mecp2
Fstl1
Slc1a2
Prrt2
Fyttd1
Dpyd
Cxadr
Srsf6
Nifk
Slc9a1
Plcb3
Flnc
Dlgap4
Ssx2ip
Arrb2
Itpr3
Mbp
Phka1
Cblb
Tle3
Sncg
Sirpa
Tubb4b
Nkap
Arx
Eef1d
Ubr5
Pttg1ip
Sirt2
Rps6
Tmpo
Taf1c
Brsk1
Rps3

Snap23
Kcnj10
Mybbp1a
Gphn
Vangl2
Ndrg2
Eif3c
Mapre3
Tp53i11
Enpp1
Afap1
Trmt11
Stxbp5
Nf1
Pkn1
Nfat5
Usp10
Mavs
Chat
Rplp1
Pi4ka
Stk10
Fer
Myh9
Fgf12
Stxbp1
Syt7
Scg2
Wrnip1
Asap1
Arfgef2
Ldlrap1
Tkt
Slc9a4
Rps10
Cntn6
Tas2r16
Bet1
Eef2
Hbb
Klc4
Csdc2
Fxyd1
Sptan1
Add2
Mprip
Tuba1b
Pf4
Abcf1
Cnksr2
Slc4a3
Rnps1
Fgf13
Dctn2
Akap13
Top2a
Usp16
Slc2a1

Mink1
Pex5l
Ptk2
Yap1
Iws1
Ei24
Gsk3a
S1pr2
Myzap
Ccdc86
Neo1
Myo5a
Dmd
Phlpp1
Wrap53
Prkd1
Erbb2
Sox10
Ube2b
Psen2
Nucks1
Atp1a2
Aktip
Rbm10
Lrrfip2
Maoa
Apba2
Lbr
Speg
Slc26a2
Micalcl
Atp1a3
Psma5
Niban1
Gpalpp1
Mical2
