# Supplementary material for: CH02 peptide promotes ex vivo expansion of umbilical cord blood-derived CD34 + hematopoietic stem/progenitor cells : CH02 peptide promotes CD34 + UCB-HSPC ex vivo expansion
Source: Acta Biochim Biophys Sin (Shanghai). 2023 Jun 28;55(10):1630–9. doi: 10.3724/abbs.2023047 (PMC10577473; doi:10.3724/abbs.2023047)
Supplement: 23018Supplementary_Figures [file 23018Supplementary_Figures.pdf]

Supplementary Figure S1

Product Name: CH02 MW: 686.72

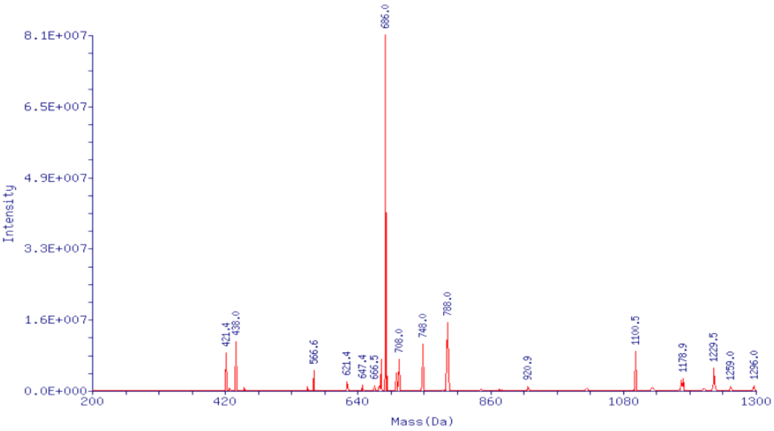

LCQ Deca XP MAX   ESI Source

Spray Voltage (kV): 5.02   Spray Current (μA):0.14   Sheath Gas Flow Rate: 35

Aux/Sweep Gas Flow Rate: 0   Capillary Voltage (V):14.85   Capillary Temp (°C):250.00

Supplementary Figure S1. Mass spectrometry analysis showed the characteristics of CH02 peptide

Supplementary Figure S2

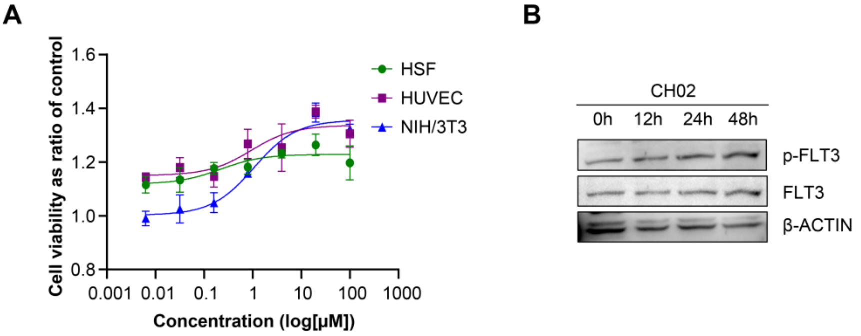

Supplementary Figure S2. CH02 peptide promoted cell proliferation and induced FLT3 pathway (A) CH02 peptide promoted HUVEC, HSF and NIH/3T3 proliferation. (B) Western blot analysis of the phosphorylated FLT3 of NIH/3T3 cell line in response to CH02 treatment at the indicated time points.

Supplementary Figure S3

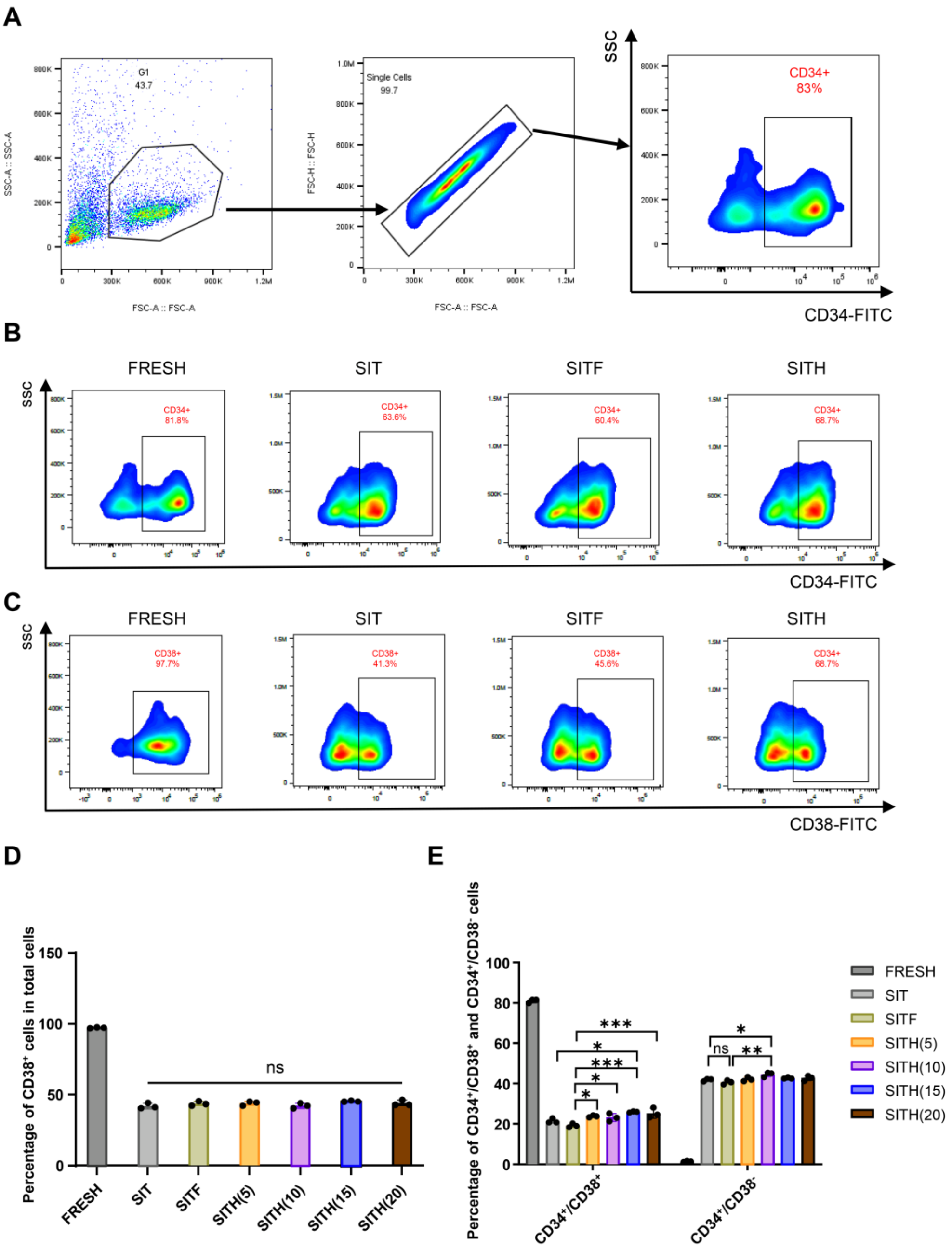

Supplementary Figure S3. Flow cytometric analysis of UCB-CD34$^+$ cells under different culture conditions (A) The frequency of CD34$^+$ cells after enrichment by flow cytometric analysis. (B,C) Flow cytometric analysis of the CD34$^+$ cells (B), CD38$^+$ cells (C), under different culture conditions. (D,E) Frequency of CD38$^+$ cells (D), CD34$^+$/CD38$^+$ cells and CD34$^+$/CD38$^-$ cells (E) under indicated culture conditions on day 0 and 7. FRESH represents CD34$^+$ cells isolated from UCB on day 0, while the SIT, SITF and SITH represent CD34$^+$ cells under indicated culture conditions on day 7 as described above, and the SITH(5), SITH(10), SITH(15) and SITH(20) represent the concentration of the CH02 peptide is 5 ng/mL, 10 ng/mL, 15 ng/mL and 20 ng/mL, respectively. Data are shown as the mean ± SD, n=3. *P<0.05, **P<0.01 and ***P<0.001; one-way ANOVA followed by Dunnett's multiple comparison test.

Supplementary Figure S4

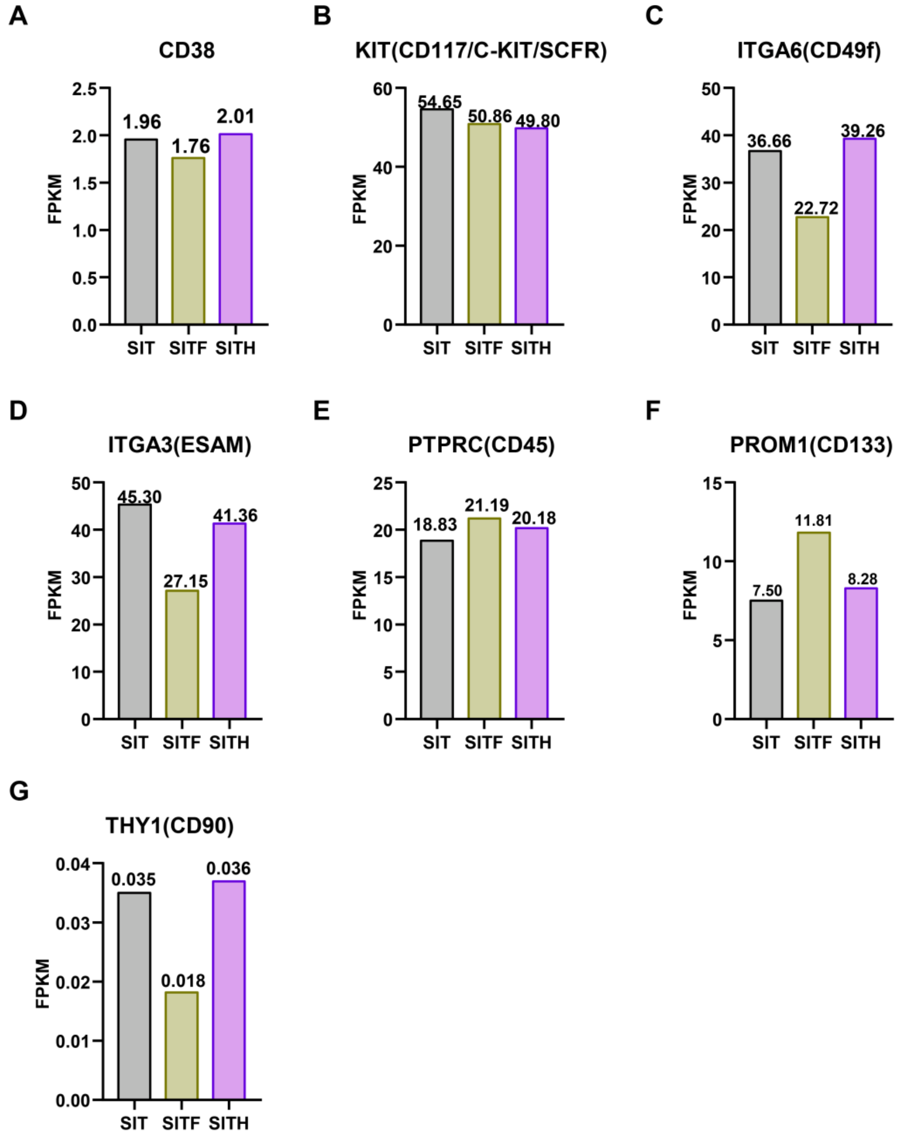

Supplementary Figure S4. RNA-Seq analysis of the stem cell-specific gene expressions

of CD34$^+$ cells under different culture conditions The genes of *CD38* (A), *C-KIT* (B), *CD49f* (C), *ESAM* (D), *CD45* (E), and *CD133* (F) are shown. The abscissa represents the culture conditions, while the ordinate represents FPKM.

Supplementary Figure S5

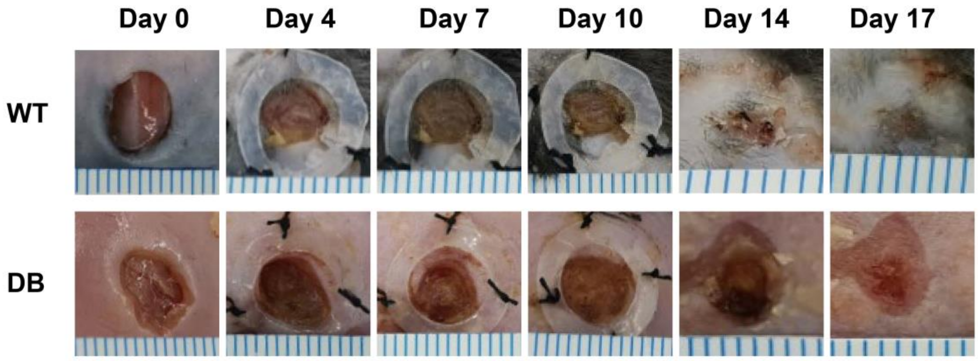

Supplementary Figure S5. The dorsal wound progression of wild-type and diabetic mice within 17 days WT, wild-type mice; DB, diabetic mice.
